# Discovery of Inhibitory Fragments That Selectively Target Spire2–FMN2 Interaction

**DOI:** 10.1021/acs.jmedchem.3c00877

**Published:** 2023-12-01

**Authors:** Radoslaw Kitel, Ewa Surmiak, Jan Borggräfe, Justyna Kalinowska-Tluscik, Przemyslaw Golik, Miroslawa Czub, Wiktor Uzar, Bogdan Musielak, Mariusz Madej, Grzegorz M. Popowicz, Grzegorz Dubin, Tad A. Holak

**Affiliations:** † Faculty of Chemistry, Jagiellonian University, Gronostajowa 2, 30-387 Kracow, Poland; ‡ Institute of Structural Biology, Molecular Targets and Therapeutics Center, Helmholtz Zentrum München, Neuherberg, 85764 München, Germany; § Bavarian NMR Center, School of Natural Sciences, Technical University of Munich Garching, 85748 München, Germany; ∥ Doctoral School of Exact and Natural Sciences, Jagiellonian University, Prof. S. Lojasiewicza 11, 30-348 Krakow, Poland; ⊥ Faculty of Biochemistry, Biophysics and Biotechnology, Jagiellonian University, Gronostajowa 7, 30-387 Cracow, Poland; # Malopolska Centre of Biotechnology, Jagiellonian University, Gronostajowa 7A, 30-387 Krakow, Poland

## Abstract

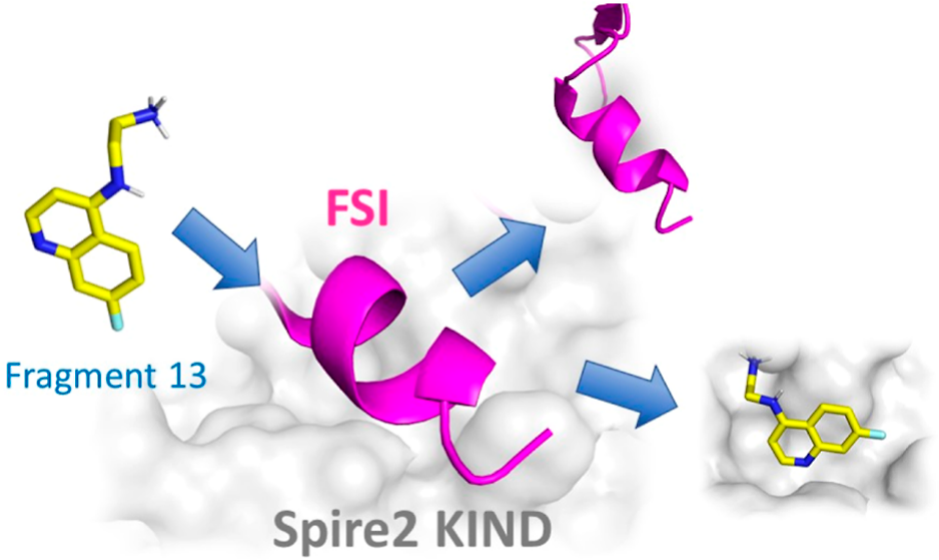

Here, we report the fragment-based drug discovery of potent and selective fragments that disrupt the Spire2–FMN2 but not the Spire1–FMN2 interaction. Hit fragments were identified in a differential scanning fluorimetry-based screen of an in-house library of 755 compounds and subsequently validated in multiple orthogonal biophysical assays, including fluorescence polarization, microscale thermophoresis, and ^1^H–^15^N HSQC nuclear magnetic resonance. Extensive structure–activity relationships combined with molecular docking followed by chemical optimization led to the discovery of compound **13**, which exhibits micromolar potency and high ligand efficiency (LE = 0.38). Therefore, this fragment represents a validated starting point for the future development of selective chemical probes targeting the Spire2–FMN2 interaction.

## 1 Introduction

Actin is the most abundant protein in cells. It has a unique ability to form long structures called actin filaments in the process of adenosine triphosphate (ATP) hydrolysis-driven polymerization. These filaments create a highly complex network that is known as the actin cytoskeleton. There are multiple functions of the actin cytoskeleton that range from cell shape maintenance, cell polarity establishment, cell migration, and motility to the regulation of many cellular processes, including vesicle trafficking, transcription, and apoptosis.^1^

The kinetic barrier of the formation of the dimer and trimer of actin (so-called nucleus or seed) is relatively high and therefore spontaneous actin polymerization is inefficient and does not occur in cells.^2^ To overcome this barrier, a large family of actin-nucleating proteins facilitates the formation of the actin nucleus.^3,4^ The whole family is divided into three main classes: Arp2/3 complex,^5^ formins,^6^ and Wiskott-Aldrich (WH2) syndrome protein domain (WASP) containing actin nucleators.^7^

Spire (p150) belongs to the WH2 domain actin nucleators. Mammalian genomes encode two spire genes, Spire1 and Spire2. Both isoforms are multidomain proteins containing an N-terminal kinase noncatalytic C-lobe domain (KIND) and a FYVE zinc finger domain located at the C-terminus. In the central region, Spire contains a cluster of four WH2 syndrome domains that are responsible for the binding of actin. The KIND of Spire is known to interact with the FMN family of formins. This interaction is mediated by a short and highly conserved formin-spire interaction (FSI) amino acid sequence located at the extreme C-terminus of formins.^8−10^

In a cellular context, Spire1/2 and its interactions with formins are engaged in many processes. It has been reported that Spire1 is involved in intracellular vesicle transport along actin fibers, thereby providing a link between the actin cytoskeleton and intracellular transport.^11^ More recently, Spire1 has been shown to play a significant role in melanosome transport.^12,13^ Additionally, Spire1/2 together with FMN2, were found to promote the assembly of nuclear actin filaments in response to DNA damage.^14^ Moreover, it has been shown that Spire-1 is specifically recruited at invadosomes, where it interacts with Rab3a GTPase, a master regulator of exocytosis. In such a manner, Spire1 affects the structure and function of invadosomes, leading to increased cell-invasion properties.^15^ Although these findings have provided important clues about the physiological function(s) of Spire1/2 in actin nucleation and beyond, it seems that the Spire1/2-formin interaction may play multiple other roles in cells. Yet, the lack of chemical tools that selectively modulate this interaction significantly hampered additional discoveries in this field.

Interestingly, other types of actin nucleators have been successfully targeted with small-molecule inhibitors like CK666^16^ and SMIFH2,^17^ targeting the Arp2/3 complex and formins, respectively. In the case of Spire, small molecules have not been discovered so far. To fill this gap, we embarked here on the discovery of Spire1/2-FMN2 interaction inhibitors. We envisioned that the development of small-molecule modulators of this interaction would provide useful tools for interrogating the complex role of Spire1/2-FMN2 in cell biology. We employed a fragment-based screening strategy to find compounds that could be used as a starting point for further optimization. Our screen identified a set of chemically related compounds that antagonize the Spire2/FMN2 but not the Spire1/FMN2 interaction. The most potent hit, **F408** showed robust activity in a battery of biochemical and biophysical methods, including fluorescence polarization (FP), microscale thermophoresis (MST), and ^1^H–^15^N HSQC nuclear magnetic resonance (NMR). The conducted structure–activity relationship (SAR) studies ultimately led to the discovery of compound **13**, which forms a strong basis for the development of cell-active chemical probes that selectively target the Spire2/FMN2 interaction.

## 2 Results and Discussion

### 2.1 Identification of Hit Fragments and Their Validation

**Figure 1:**
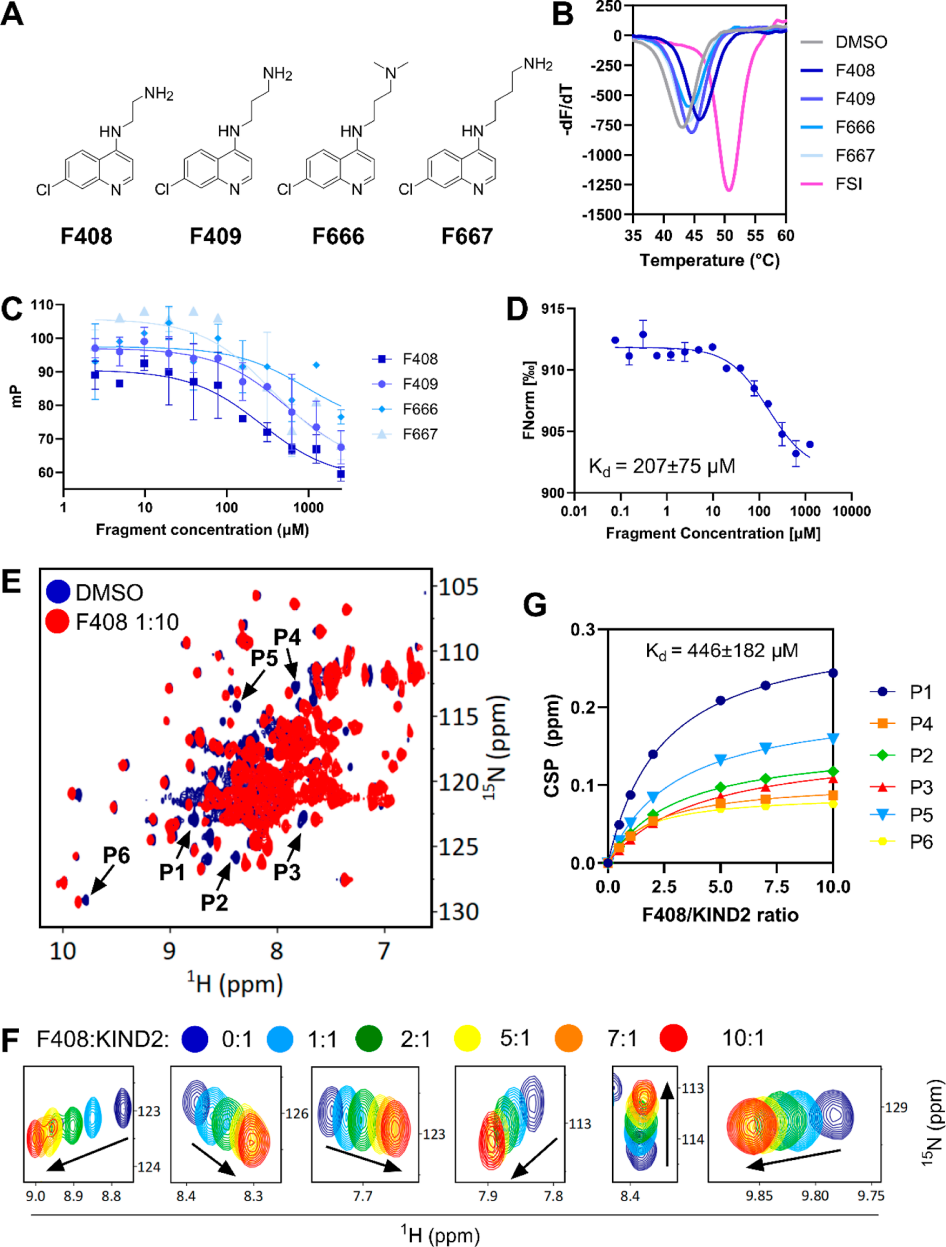
Biophysical characteristics of KIND2 hits; (A) chemical structures of initial hits. (B) Melting curves of the KIND2 in the presence of 1 mM hit fragments and FSI peptide shown as a positive control. (C) Representative FP dose–response curves for fragments **F408**, **F409**, **F666**, and **F667**. (D) Representative MST curve for **F408**. (E) ^1^H–^15^N HSQC spectra of uniformly ^15^N-labeled KIND2 in the presence of 5% DMSO (blue) and 2.8 mM (red) fragment hit **F408**. (F) Insets showing the six most perturbed peaks (P1–P6) with increasing concentrations of fragment **F408** (protein/ligand ratios indicated above). (G) A plot of normalized CSP as a function of protein/ligand ratio was used to estimate the average *K*_d_ of **F408** to KIND2.

To identify binders of the KIND of Spire1/2, we screened an in-house fragment library of 755 compounds against recombinant constructs of KIND1 (36–236 aa) and KIND2 (18–207 aa). Differential scanning fluorimetry (DSF) served as a primary screening technique (Table S1). Fragments were screened as singletons at a final concentration of 1 mM (2% DMSO). All experiments were done using a synthetic FSI peptide comprising 23 amino acid residues (NH_2_-GKSLYKIKPRHDSGIKAKISMKT-OH) from the C-terminus of human FMN2, which serves in the assay as a positive control. As hit criteria, we arbitrarily set values of positive and negative thermal shift for +0.8 and −2.0 °C, respectively. This resulted in 19 and 47 hit fragments for the KIND1 and KIND2, respectively, yielding a hit rate of 2.5% (KIND1) and 6.2% (KIND2). Out of 19 hits for KIND1, only four exhibit thermal shift values greater than Δ*T*_m_ > 1.0 °C. Similarly, for KIND2, most of the fragment hits destabilized the protein and only 5 hits exerted positive Δ*T*_m_ values. Notably, among them, four hits (**F408**, **F409**, **F666**, and **F667**) shared the same quinoline scaffold decorated at the 4-position with alkyl chains containing primary or substituted amine (Figure 1A). These hits induced a thermal stabilization of KIND2 in the range of Δ*T*_m_ +1.0 to +2.9 °C, with fragment **F408** inducing the highest thermal shift (Figure 1B). Interestingly, none of these fragments were flagged as hits for KIND1, and therefore we decided to progress this compound series for further detailed investigation.

To account for the potential ambiguity in the DSF data, the potency of selected KIND2 hits was further assessed by orthogonal methods. To determine whether the hits target the Spire2/FMN2 interface, we developed a FP competitive assay using the FITC-labeled 23-mer FSI peptide (FITC-Ahx-GKSLYKIKPRHDSGIKAKISMKT-OH) as a reporter probe (Figure S2). In this assay, hit fragments were measured in a dose–response (2.44–2500 μM, 5% DMSO) format, yielding the inhibitory activity (IC_50_) toward KIND2 in the range of 268–938 μM, confirming the highest potency of fragment **F408** (Figure 1C). Taking into account the competitive nature of the FP assay, these results suggest that the discovered fragments target the site originally occupied by the FSI tail of FMN2 formin.

Before progressing further, we resynthesized fragment **F408** and measured its affinity toward KIND2. The latter one was done in a MST assay using a fluorescently labeled His6-tagged KIND2 construct. This resulted in a measured affinity of 207 ± 75 μM for **F408** (Figure 1D). Interestingly, **F408** did not bind to the mutant variant of KIND2 Y106A (Supporting Information, Figure S3), supporting previous results that this fragment is located on the KIND2/FSI interface. Finally, using a uniformly ^15^N-labeled KIND2, we carried out a series of protein-observed NMR experiments. We monitored the chemical shift perturbations (CSP)^18^ upon titration of KIND2 with an increasing concentration of fragment **F408** (protein–ligand ratio 1:1–1:10). As seen in Figure 1E,F, **F408** induced prominent chemical perturbations of multiple cross-peaks. The concentrated-dependent gradual chemical shift changes in a series of ^1^H–^15^N HSQC spectra facilitated the quantification of KIND2-F408 binding affinity with a *K*_d_ = 446 ± 182 μM (Figure 1G).

Given the MW equal to 222 and the heavy atom count (HAC) of 15, the ligand efficiency results in LE = 0.32, making compound **F408**, a good starting point for further elaboration.^19^ Additionally, **F408** did not bind to KIND1 and therefore opens an avenue for the development of selective inhibitors of the Spire2/FMN2 interaction. Finally, the quinoline scaffold offers many advantages, especially for further fragment elaboration since 2-, 3-, 5-, 6-, 7-, and 8-carbons can be used for decoration with other substituents.

### 2.2 SAR of Fragments

**Scheme 1:**
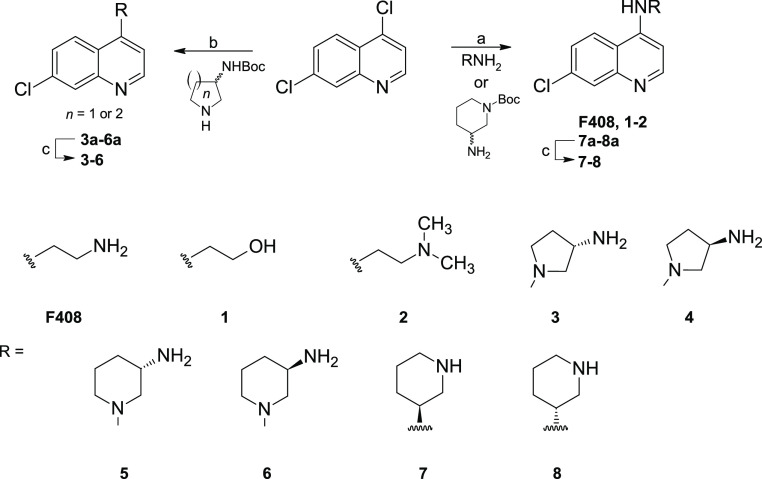
Synthesis of 4-Substituted Fragments Reaction conditions: (a) primary amine component, neat, sealed tube, 110 °C or 95 °C, 8−16 h; (b) cyclic secondary amine component, neat, sealed tube, 95 °C, 16 h; (c) 4M HCl/dioxane in DCM, 0 °C to RT, overnight.

The construction of our fragment library allows us to establish an initial SAR by catalogue (Table 1). Testing the inhibitory activity of near neighbors of F408 using the FP assay revealed additional key structural features of the fragments. For instance, the installation of a terminal hydroxyl group proved detrimental to KIND2/FSI inhibition with a complete loss in activity compared to the compound with an amine substituent (**F410** vs **F409**). Removal of the linker also led to a decreased potency (**F668**). Installation of the bulky morpholine fragment in place of a primary amine in compound **F669** and cyclization of the linker to piperazine (**F671**) have also resulted in a complete drop in potency. To assess the importance of other functional groups at position 4 of the quinoline scaffold, we synthesized additional close analogues of **F408** (Scheme 1). Replacement of primary amine to a hydroxyl group, as previously shown for compounds bearing a C-3 linker, was not tolerated also in compounds with a C-2 linkage (**1**). Interestingly, shortening the linker to C-2 with terminal dimethyl substituted amine in compound **2** also led to a loss of potency. Moreover, compounds **3–8** had no activity toward KIND1 and KIND2. From this study, it is evident that the primary terminal amine is indispensable for the activity and the optimal length of the linker between quinoline nitrogen and the terminal amine is C-2.

**Table 1:**
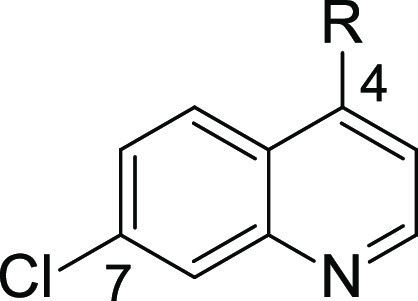

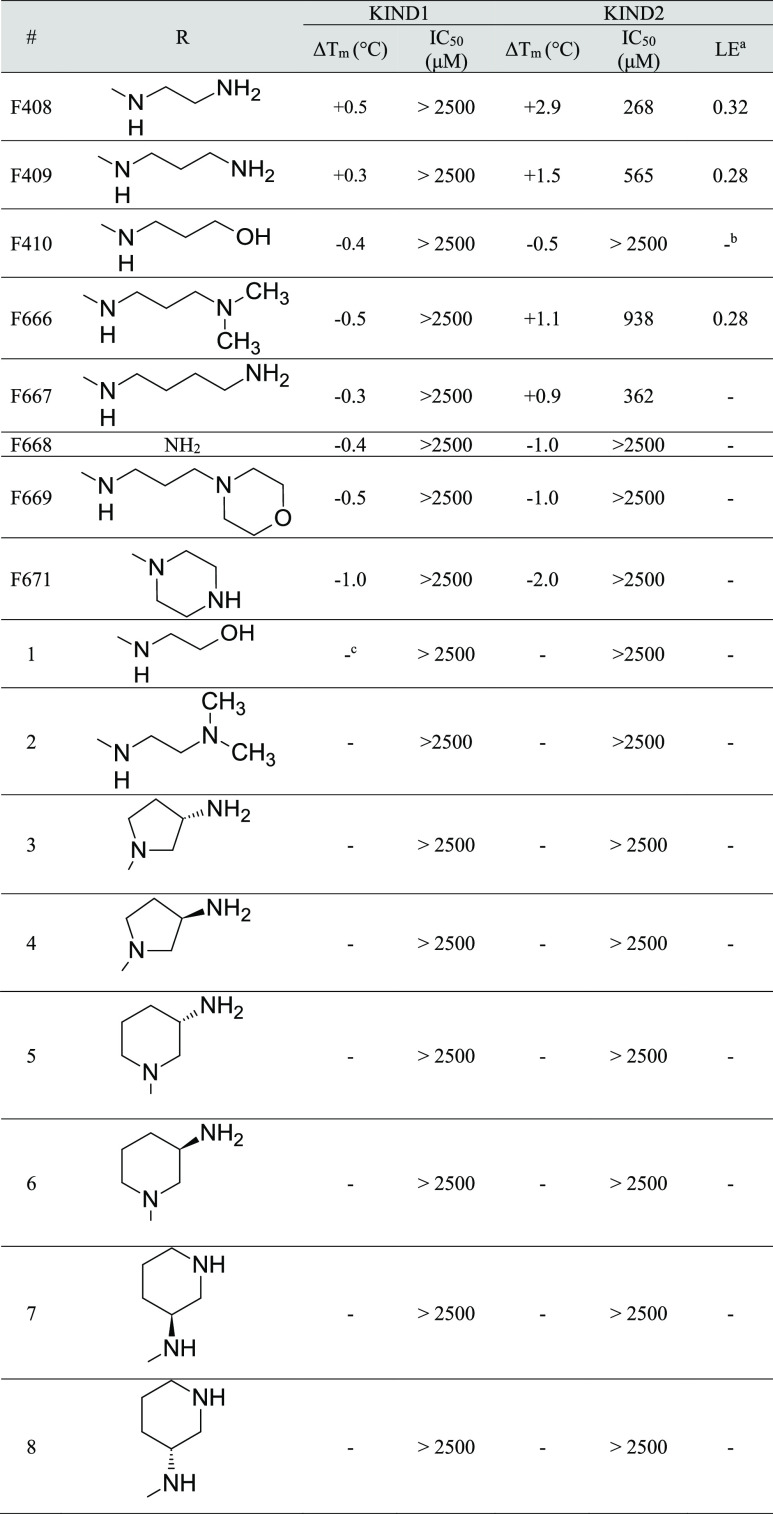
SAR of 4-Substituted Fragments

a LE = −*RT* ln(IC_50_)/HA.

b Not calculated.

c Not determined.

**Scheme 2:**
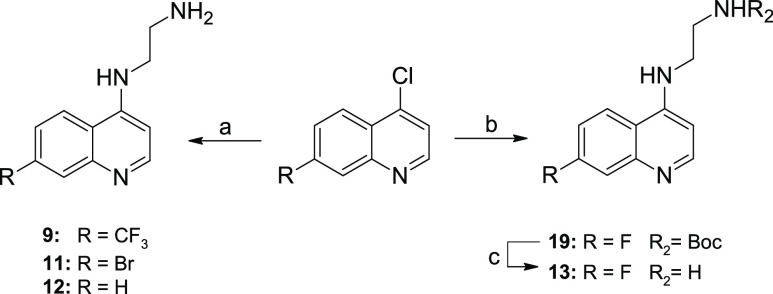
Synthesis of 7-Substituted Fragments from Chloroquinolines Reagents and conditions: (a) 1,2-ethanediamine, neat, sealed tube, 110 °C, 16 h. (b) *N*-Boc-1,2-ethanediamine, neat, sealed tube, 95 °C, 16 h. (c) 4 M HCl in dioxane, DCM, 0 °C-rt, 16 h.

**Scheme 3:**
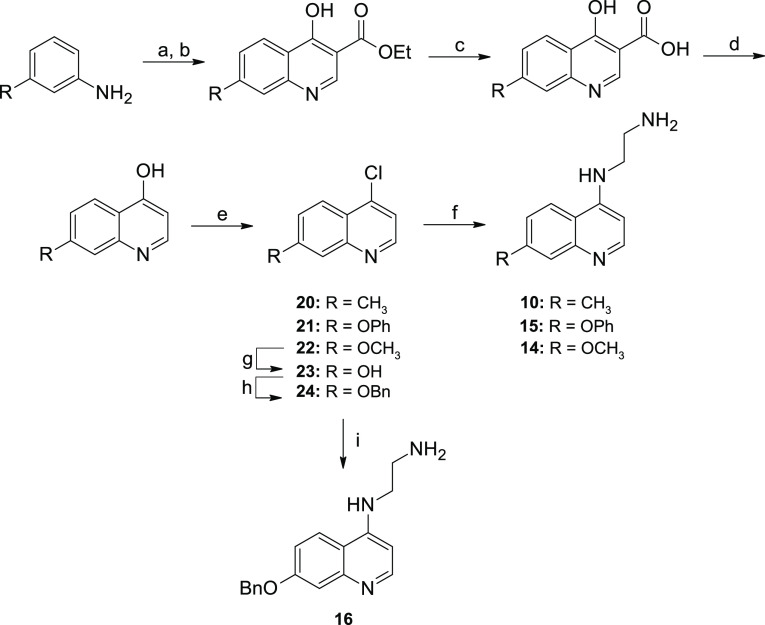
Synthesis of 7-Substituted Fragments from Anilines Reagents and conditions: (a) aniline, diethyl 2-ethoxymethylenemalonate, EtOH, reflux, 2 h. (b) Dowtherm, 260 °C, 15–30 min. (c) 10% NaOH, MeOH, 3 h. (d) Dowtherm, 240 °C, 1 h. (e) POCl_3_, 110 °C, 4 h. (f) Ethylenediamine, neat, sealed tube, 110 °C, 16 h. (g) 48% HBr, AcOH, 24 h. (h) BrBn, K_2_CO_3_, DMF, 16 h. (i) Ethylenediamine, neat, sealed tube, 110 °C, 16 h.

**Scheme 4:**
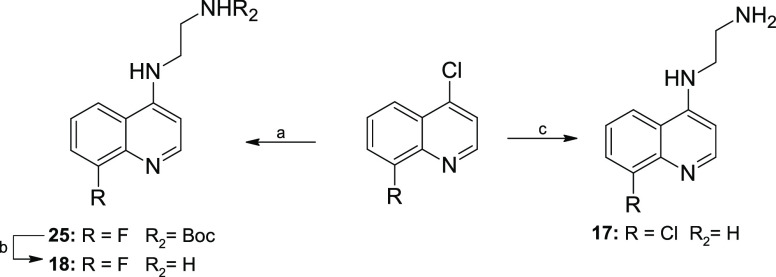
Synthesis of 8-Substituted Fragments Reagents and conditions: (a) *N*-Boc-ethylenediamine, neat, sealed tube, 95 °C, 16 h. (b) 4 M HCl in dioxane, DCM, 0 °C-rt, 16 h. (c) Ethylenediamine, neat, sealed tube, 110 °C, 6 h.

Being unsuccessful in finding more potent analogues of **F408** through structural modification of the 4-position, we put our attention to modifications of position 7. In this series, we decided to keep the C2-linker with the primary amine at the 4-position. To access compounds modified at position 7, we transformed commercially available quinoline chlorides and then reacted them with ethylenediamine. The same synthetic approach was applied for quinoline-4-ols, except that before the S_N_Ar reaction with amine, they were converted into the corresponding chlorides (Scheme 2). Those quinolines that could not be obtained from commercial sources were obtained in Gould–Jacobs synthesis (Scheme 3), transformed into 4-chloro derivatives, and finally reacted with ethylenediamine. In summary, we synthesized 8 new derivatives (**9–16**) and tested them in both DSF and FP competition assay. Table 2 shows the SAR results from installing different substituents at the 7-position of the quinoline ring system. The replacement of chlorine atoms with trifluoromethyl (**9**), methyl (**10**), bromine (**11**), phenoxy (**15**), and benzyl groups (**16**) generally led to a drop in the potency. On the other hand, the installation of the methoxy group provided an approximately 3-fold more active compound **14** in terms of inhibitory potency. More notably, the introduction of fluorine (**13**) resulted in a 4-fold boost in inhibitory activity and an improved LE value of 0.38. In line with this, **13** demonstrated the highest Δ*T*_m_ of all compounds synthesized so far. We also obtained analogues of **F408** and **13** that differ in the position of halogen atoms (**17** and **18**, Scheme 4). Interestingly, changing the position of halogen (Cl or F) from the 7 to 8 position led to a complete loss of activity, as demonstrated for compounds **17** and **18**.

**Table 2:**
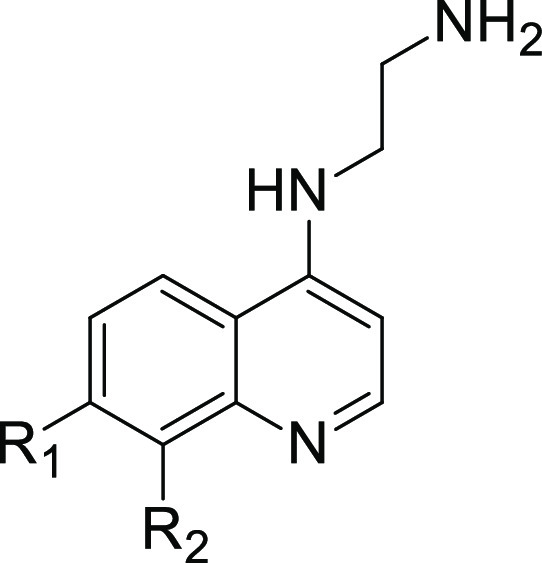
Effect of 7- and 8-Substitutions on the Activity of Fragments

#	R_1_	R_2_	Δ*T*_m_ (°C)	IC_50_ (μM)	LE^a^
9	CF_3_	H	–0.4	>2500	b
10	Me	H	2.0	>2500	
11	Br	H	+1.2	>2500	
F408	Cl	H	+2.9	268	0.32
12	H	H	+1.6	448	0.33
**13**	**F**	**H**	**+5.6**	**62**	**0.38**
14	OMe	H	+3.6	103	0.34
15	OPh	H	–0.1	>2500	
16	OBn	H	0.0	>2500	
17	H	Cl	+0.8	>2500	
18	H	F	+0.8	>2500	

a LE = −*RT* ln(IC_50_)/HA.

b Not calculated.

**Figure 2:**
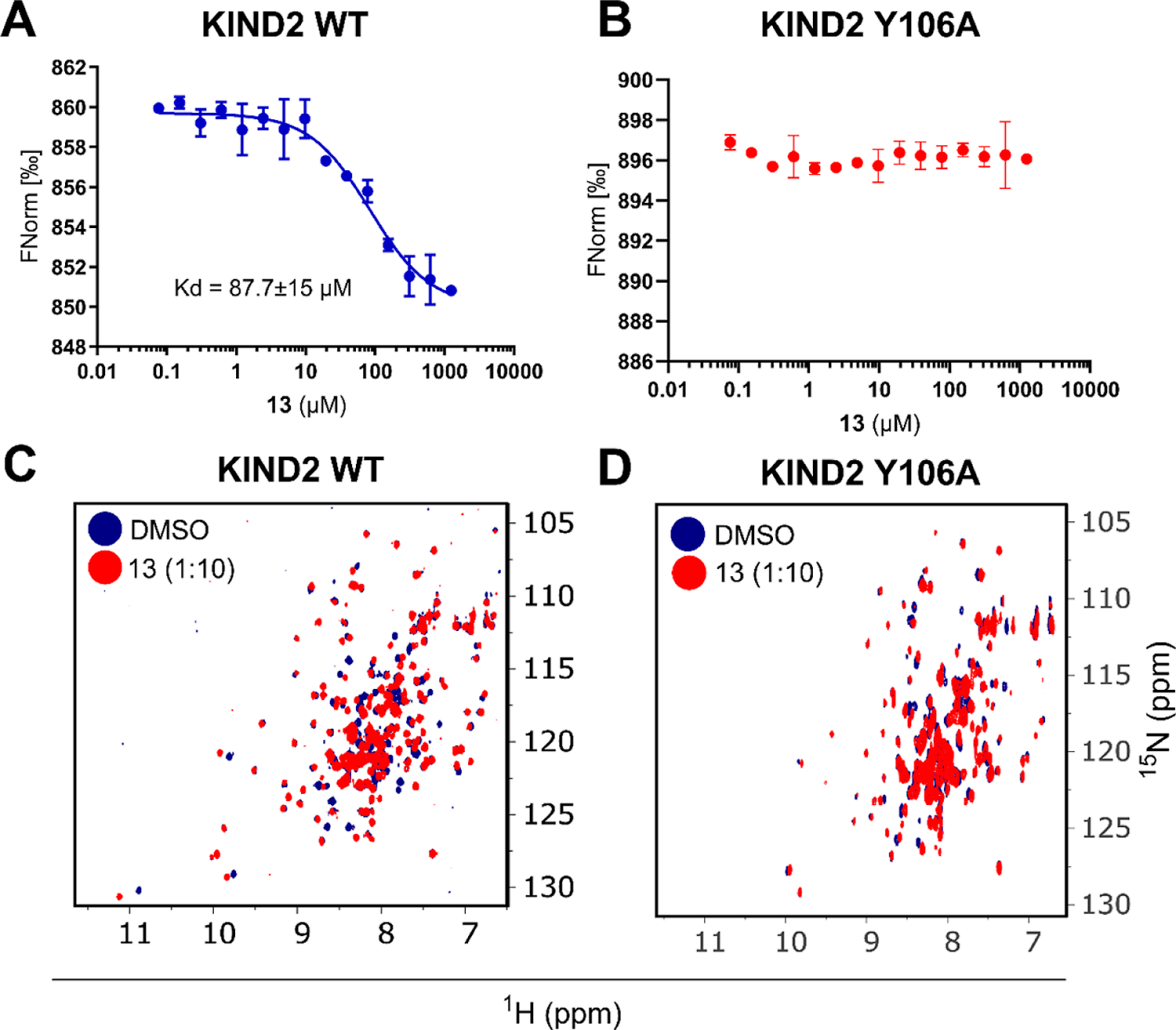
Biophysical characteristics of compound **13**; (A) MST binding dose–response curve for **13** measured for KIND2. (B) ^1^H–^15^N HSQC spectra of uniformly ^15^N-labeled KIND2 in the presence of 5% DMSO (blue) and a 10-fold excess of **13** (red). (C) MST binding dose–response curve for **13** measured for the KIND2 Y106A domain. (D) ^1^H–^15^N HSQC spectra of uniformly ^15^N-labeled KIND2 Y106A in the presence of 5% DMSO (blue) and a 10-fold excess of **13** (red).

Furthermore, to characterize compound **13** in terms of binding, we measured the affinity of this fragment toward KIND2 in a MST assay, which yielded *K*_d_ = 87.7 ± 15 μM (Figure 2A). Strikingly, **13** induced a strong CSP of multiple residues as evidenced by the overlaid spectra of the ^15^N-KIND2 in the absence or presence of the compound (Figures 2B and S4). The affinity derived from this experiment yielded *K*_d_ = 79.8 ± 6 μM, which is in perfect agreement with the one established in the MST assay. In contrast, **13** did not show any binding in the MST assay when tested on a KIND2 Y106A (Figure 2B). In line with this, no significant CSPs were recorded in the NMR titration experiment (Figure 2D).

### 2.3 In Silico Modeling of Binding of Compound **13**

**Figure 3:**
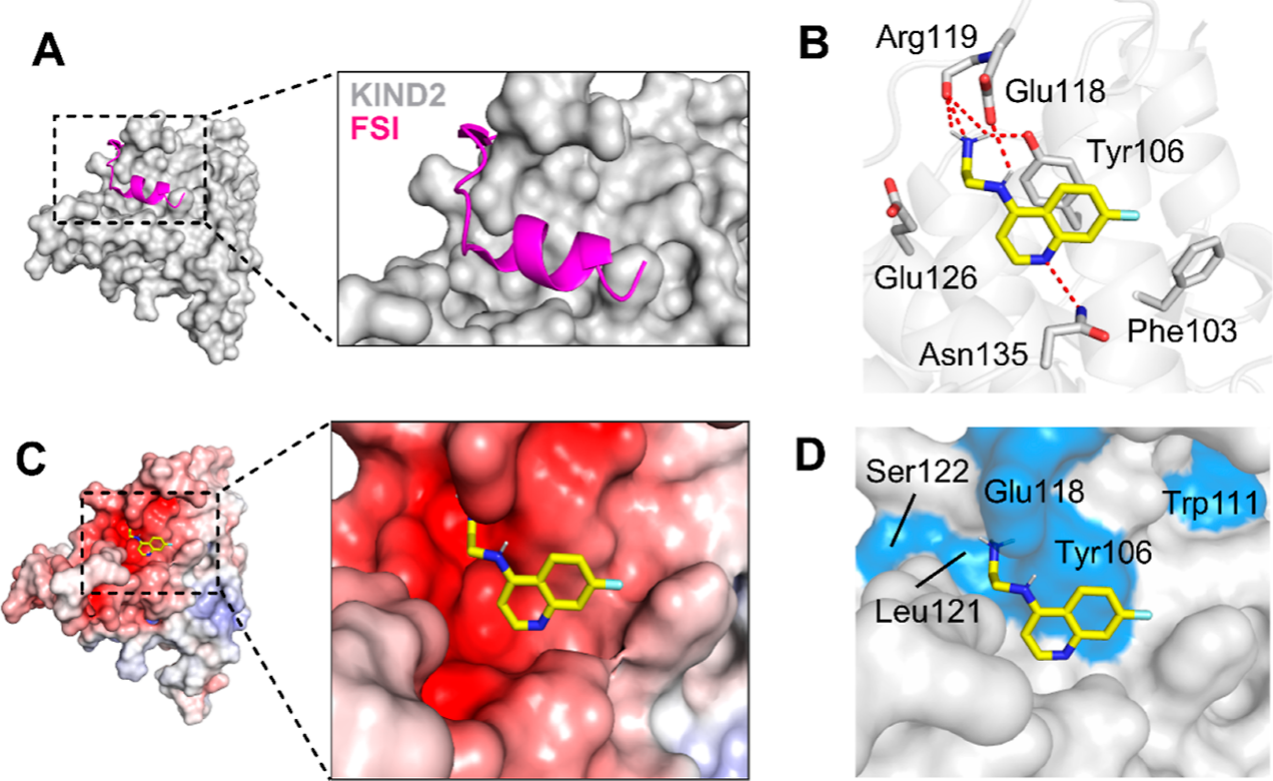
Compound **13** targets the KIND2–FSI interface. (A) Homology model of the KIND2–FSI complex; (B) proposed binding mode of **13** in the FSI binding site of KIND2; and charge interactions and hydrogen bonds are shown as red dashed lines. (C) Electrostatic surface representation of the KIND2 with **13** shown as yellow sticks. Red, negative (−10 kT e^–1^); blue, positive (+10 kT e^–1^). (D) Mapping of substantially perturbed residues (blue) on the model of the KIND2.

Despite many efforts, throughout the project duration, we were unsuccessful in obtaining the X-ray structure of the KIND2, in complex neither with FSI peptide nor with the most potent fragments (**F408** and **13**). Therefore, to gain insights into the possible binding mode of **13**, we carried out molecular docking using the homology model of the KIND2. We used the published high-resolution X-ray structure of a human KIND1 in complex with the FSI peptide (PDB code 2YLE, 1.8 Å) to (1) predict the binding mode of the corresponding peptide to KIND2 (Figure 3A) and (2) dock the most potent fragment **13** into the binding site of the FSI peptide. The calculations were done independently using Vina and GOLD software. The most anticipated binding mode of **13** is shown in Figure 3B. According to this model, the quinoline scaffold of **13** is located deeply in the hydrophobic pocket normally occupied by two Ile residues (Ile1714 and Ile1718) of the FSI peptide and forms a stacking interaction with Tyr106. Additionally, the nitrogen atom of quinoline is located in close proximity (ca. 2.9 Å) to Asn135 and forms a hydrogen bond with its amine group. The aliphatic linker rests between two negatively charged glutamic acid side chains (Glu118 and Glu126). The shallow tunnel formed by these two residues does not allow accommodation of more bulky and rigid aliphatic rings, providing a likely explanation for why fragments bearing such substituents (**3–8**) were completely inactive. The positively charged amine group of **13** forms multiple electrostatic contacts with Glu118 and hydrogen bonds with the hydroxyl group of Tyr106 and the backbone carbonyl group of Arg119. Finally, the 7-fluoro-substituted ring points toward Phe103 and forms weak van der Waals interactions. Overall, analyzing the electrostatic surface of the KIND2 model, **13** docks in a highly negatively charged groove where it is stabilized by electrostatic interactions (Figure 3C).

To support the anticipated binding mode, we have done a series of NMR experiments with ^15^N/^13^C-labeled KIND2 to provide backbone amide resonance assignments (Figure S3). However, we were only able to assign roughly 40% of the residues, and most of the assignable residues lay outside the binding site. The assignment did not provide the resonance of Tyr106 that lay in the central part of the pocket. Yet, the ^15^N–^1^H HSQC spectrum of KIND2 mutant Y106A identified the location of Tyr106. Taking this into account, mapping the perturbed residues clearly indicates that compound **13** as well as its less active predecessor **F408**, binds to the binding cleft that is normally occupied by FSI residues (Figure 3C). The intense CSP of residues (Figure S4) that form a flexible loop, namely, glutamate residues 117 and 118 and, to some extent, Glu115, Gly 112, and Trp111, is a result of the protrusion of the ethylenediamine moiety of **13** into the narrow channel formed between Glu126 and Glu117. Additionally, the perturbations of Leu121 and Ser122 are likely due to the presence of the pyridine ring of the quinoline scaffold that rests in close proximity.

**Figure 4:**
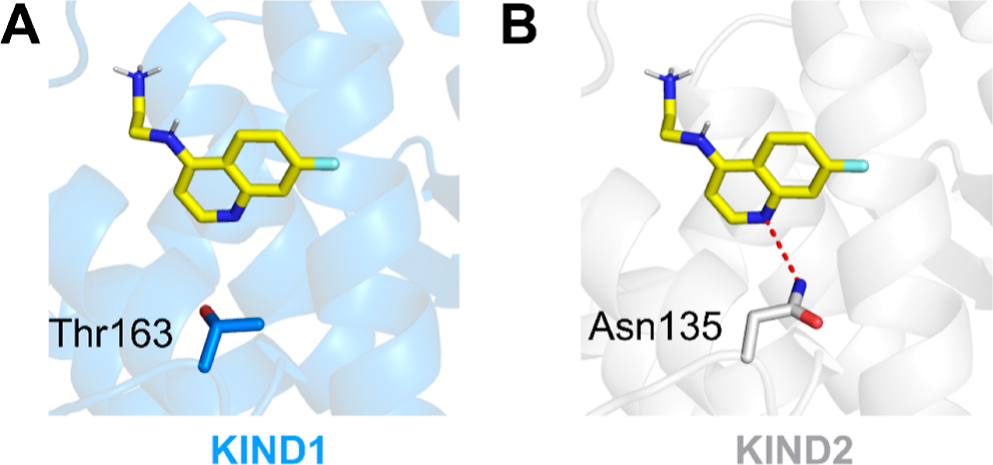
Structural basis for selective binding of **13** to KIND2. (A) Docking of **13** to the FSI binding site of KIND1 (PDB: 3R7G); the hydroxyl group of Thr163 is ca. 4.3 Å away from the quinoline nitrogen atom and therefore could not be involved in hydrogen bonding; (B) binding mode of **13** in the FSI binding site of the KIND2; and hydrogen bond between the nitrogen of quinoline and the amine group of Asn135 is shown as a red dashed line.

The proposed binding mode of **13** is in agreement with the observed SAR established earlier and may serve as a basis for further compound optimization. Additionally, the comparison of a homology model of KIND2 with the KIND1 X-ray structure (PDB: 2YLE) revealed key differences in the FSI binding pocket (Figure 4). The main difference is attributed to the replacement of the KIND2 Asn135 side chain with the corresponding Thr163 in KIND2. This slight structural change prevents the formation of a strong hydrogen bond between KIND1 and the nitrogen atom of the quinoline ring at the bottom of the pocket. These provide a likely explanation for the lack of activity of KIND2 fragments that we observed for the KIND1.

## 3 Conclusions

In summary, here we disclose well-characterized inhibitory fragments that bind with micromolar potency to the KIND2 of Spire2, thereby impairing its interaction with formin, FMN2. The data provided in our work paves the way for the downstream development of more potent molecules with cell activity. Further optimization should focus on addressing the surrounding subpockets that lie nearby the binding site occupied by hit fragments. Additionally, balancing the lipophilicity due to the presence of a primary amine group that seems to be obligatory for affinity should be taken into consideration. Studies are currently underway to optimize analogues of **13**, which will be reported in due course.

## 4 Experimental Section

### 4.1 General Procedures

All chemicals used for the synthesis were obtained from commercial suppliers (Sigma-Aldrich, Idalia, Ambeed, and Apollo Scientific) and used without further purification. ^1^H and ^13^C NMR spectra were recorded by using either a 600 MHz Bruker Avance spectrometer or a 400 MHz Jeol instrument. Samples were dissolved in DMSO-*d*_6_ (Sigma-Aldrich, 99.80% D). Chemical shifts were reported in parts per million (ppm, δ) downfield from tetramethylsilane (TMS). Coupling constants (*J*) were expressed in Hz. The following abbreviations (or a combination, thereof) were used to describe splitting patterns: s, singlet; d, doublet; t, triplet; q, quartet; quint, quintet; sept, septet; m, multiplet; and br, broad. All final compounds were of 95% purity or higher unless otherwise noted, as indicated by analytical LC–MS. The liquid chromatography–mass spectrometry (LC–MS) measurements were performed on a LC triple quadrupole mass spectrometer LCMS-8045 with the electrospray ionization probe (Shimadzu Corporation, Japan). Chromatographic separations were carried out using the ReproSil pHoenix C18 column, 2.0 × 100 mm, and 1.9 μm particle size (Dr. Maisch, Germany), equipped with a Shim-pack Velox UHPLC precolumn filter (Shimadzu Corporation, Japan). The column was maintained at 40 °C and eluted under gradient conditions using 99–5% of eluent A over 12 min at a flow rate of 0.2 mL min^–1^. Eluent A: water/formic acid (0.1%, v/v); eluent B: acetonitrile.

### 4.2 Synthesis

#### 4.2.1 General Procedure A: Alkylation with Ethylenediamine

Appropriate 4-chloroquinoline (0.61–4.32 mmol, 1 equiv) was reacted at 110 °C in a sealed tube with ethylenediamine (4–10 equiv) for 4–16 h. After cooling to rt, the mixture was taken up with a sat. KHCO_3_ solution. The mixture was extracted to DCM (3 × 20 mL), and the combined organic phases were dried over Na_2_SO_4_, filtered, concentrated with silica gel, and directly loaded on the column. Elution was carried out as specified in individual cases.

#### 4.2.2 General Procedure B: Synthesis of Compounds with N-Boc-Protected Amines

Appropriate 4-chloroquinoline (0.5–3.03 mmol, 1 equiv) was reacted at 95 °C in a sealed tube with appropriate N-Boc-protected amine (5 equiv) for 4–16 h. Upon completion of the reaction, the mixture was cooled to rt. The mixture was taken up with a sat. KHCO_3_ solution. The mixture was extracted to DCM (3 × 20 mL), and the combined organic phases were dried over Na_2_SO_4_, filtered, concentrated with silica gel, and directly loaded on the column. Elution was carried out as specified in the individual cases.

#### 4.2.3 General Procedure C: Deprotection of Boc-Protected Compounds

The appropriate Boc-protected compound (0.47–5.05 mmol, 1 equiv) was dissolved in DCM. The reaction mixture was cooled to 0 °C, and 4 M HCl in dioxane (10 equiv) was added dropwise. Upon completion of the reaction (typically 16 h), the reaction mixture was diluted with DCM and washed with 10% NaOH. The organic phase was washed with brine (2×) and water (2×), dried, concentrated with silica gel, and purified using an appropriate elution system.

#### 4.2.4 General Procedure D: Synthesis of 7-Substituted 4-Chloroquinolines

Appropriate aniline (1 equiv) was dissolved in anhydrous EtOH. Next, diethyl 2-ethoxymethylenemalonate (1 equiv) was added, and the reaction mixture was heated to reflux for 2 h. Upon cooling to rt, the precipitated solid was filtered off, washed with ice-cooled EtOH, and used directly in the next step without purification. The crude solid was portion-wise added to Dowtherm and heated to 260 °C. Upon cooling and dilution with Et_2_O, a large amount of solid precipitated. The solid was filtered off, washed with MeOH, and directly used in the next step without purification. The crude ester was added to 10% NaOH (containing 10% v/v of MeOH). The mixture was heated to 110 °C for 3 h. Upon cooling to rt, the reaction mixture was acidified with sat. HCl and a large amount of solid precipitated. The solid was used in the next step without purification. The solid was finally added to Dowtherm and heated to 240 °C for 1 h. The product precipitated as a mixture of two regioisomers of quinoline-4-ol which were successfully separated using column chromatography with an appropriate elution system. The desired product was then suspended in POCl_3_. The resulting mixture was refluxed for 16 h. The mixture was then cooled to rt, quenched with water, and extracted with ethyl acetate. The combined organic extracts were washed with water and brine, dried over sodium sulfate (Na_2_SO_4_), and concentrated to afford the appropriate 4-chloroquinoline. Where indicated, compounds were purified by using an appropriate elution system.

##### 4.2.4.1 *tert*-Butyl [(3*S*)-1-(7-chloroquinolin-4-yl)pyrrolidin-3-yl]carbamate (**3a**)

Obtained following the general procedure B, starting from 4,7-dichloroquinoline (0.5 g, 2.52 mmol, 1 equiv) and (*S*)-3-(Boc-amino)pyrrolidine (2.35 g, 12.62 mmol, 5 equiv) as an orange oil (0.85 g, yield: 97%). Elution of the product with CHCl_3_/MeOH 40:1 (v/v).

^1^H NMR (400 MHz, DMSO-*d*_6_): δ 8.35 (d, *J* = 5.5 Hz, 1H), 8.20 (d, *J* = 9.2 Hz, 1H), 7.77 (d, *J* = 2.3 Hz, 1H), 7.33 (dd, *J* = 9.2, 2.4 Hz, 1H), 7.24 (d, *J* = 6.2 Hz, 1H), 6.47 (d, *J* = 5.6 Hz, 1H), 4.10 (m, 1H), 3.82 (m, 1H), 3.73 (m, 1H), 3.65–3.53 (m, 1H), 3.44 (m, 1H), 2.16–2.03 (m, 1H), 1.98–1.85 (m, 1H), 1.34 (s, 9H). ^13^C NMR (101 MHz, DMSO-*d*_6_): δ 155.8, 152.1, 151.3, 151.1, 133.5, 128.0, 127.9, 123.7, 119.5, 103.7, 79.7, 78.5, 57.6, 50.5, 50.3, 31.1, 28.7.

##### 4.2.4.2 *tert*-Butyl [(3*R*)-1-(7-chloroquinolin-4-yl)pyrrolidin-3-yl]carbamate (**4a**)

Obtained following the general procedure B, starting from 4,7-dichloroquinoline (0.3 g, 1.51 mmol, 1 equiv) and (*R*)-3-(Boc-amino)pyrrolidine (1.13 g, 6.06 mmol, 4 equiv) as a beige foam (0.38 g, yield: 71%). Elution of the product with CHCl_3_/MeOH 40:1 (v/v).

^1^H NMR (400 MHz, DMSO-*d*_6_): δ 8.34 (d, *J* = 5.5 Hz, 1H), 8.20 (d, *J* = 9.2 Hz, 1H), 7.77 (d, *J* = 2.3 Hz, 1H), 7.32 (dd, *J* = 9.2, 2.3 Hz, 1H), 7.24 (d, *J* = 6.1 Hz, 1H), 6.46 (d, *J* = 5.6 Hz, 1H), 4.12 (m, 1H), 3.82 (m, 1H), 3.73 (m, 1H), 3.63–3.54 (m), 3.44 (m, 1H), 2.15–2.04 (m, 1H), 1.96–1.85 (m, 1H), 1.34 (s, 9H). ^13^C NMR (101 MHz, DMSO-*d*_6_): δ 155.8, 152.1, 151.3, 151.0, 133.5, 127.9, 123.7, 119.5, 103.7, 78.5, 57.6, 50.5, 50.3, 31.1, 28.7.

##### 4.2.4.3 *tert*-Butyl (*S*)-(1-(7-chloroquinolin-4-yl)piperidin-3-yl)carbamate (**5a**)

Obtained following the general procedure B, starting from 4,7-dichloroquinoline (0.1 g, 0.5 mmol, 1 equiv) and (*S*)-3-(Boc-amino)piperidine (0.2 g, 1.0 mmol, 2 equiv) as a yellow amorphic solid (0.12 g, yield: 66%). Elution of the product with CHCl_3_/MeOH 50:1 (v/v).

^1^H NMR (400 MHz, DMSO-*d*_6_) NMR (400 MHz, DMSO-*d*_6_): δ 8.62 (d, *J* = 5.0 Hz, 1H), 7.97 (d, *J* = 9.0 Hz, 1H), 7.90 (d, *J* = 1.9 Hz, 1H), 7.45 (d, *J* = 7.8 Hz, 1H), 7.00 (d, *J* = 7.4 Hz, 1H), 6.94 (d, *J* = 5.0 Hz, 1H), 3.62 (m, 1H), 3.40 (d, *J* = 10.9 Hz, 1H), 2.77 (t, *J* = 10.7 Hz, 1H), 2.62 (t, *J* = 10.3 Hz, 1H), 1.83 (m, 1H), 1.70 (m, 1H), 1.46–1.40 (m, 1H), 1.37 (s, 9H). ^13^C NMR (101 MHz, DMSO-*d*_6_): δ 157.1, 155.8, 153.0, 150.8, 135.0, 128.4, 126.6, 126.0, 121.6, 110.8, 79.9, 78.6, 57.9, 52.6, 47.6, 30.4, 28.9, 24.6.

##### 4.2.4.4 *tert*-Butyl (*R*)-(1-(7-chloroquinolin-4-yl)piperidin-3-yl)carbamate (**6a**)

Obtained following the general procedure B, starting from 4,7-dichloroquinoline (0.25 g, 1.26 mmol, 1 equiv) and (*R*)-3-(Boc-amino)piperidine (1.01 g, 5.05 mmol, 4 equiv) as a transparent oil that solidifies upon storage in the fridge (0.39 g, yield: 84%). Elution of the product with CHCl_3_/MeOH 50:1 (v/v).

^1^H NMR (400 MHz, DMSO-*d*_6_) NMR (400 MHz, DMSO-*d*_6_): δ 8.63 (d, *J* = 5.0 Hz, 1H), 7.98 (d, *J* = 9.0 Hz, 1H), 7.92 (d, *J* = 1.9 Hz, 1H), 7.49 (d, *J* = 7.8 Hz, 1H), 7.00 (d, *J* = 7.4 Hz, 1H), 6.94 (d, *J* = 5.0 Hz, 1H), 3.64 (m, 1H), 3.43 (d, *J* = 10.9 Hz, 1H), 2.78 (t, *J* = 10.7 Hz, 1H), 2.62 (t, *J* = 10.3 Hz, 1H), 1.85 (m, 1H), 1.72 (m, 1H), 1.44–1.38 (m, 1H), 1.35 (s, 9H). ^13^C NMR (101 MHz, DMSO-*d*_6_): δ 156.9, 155.6, 152.7, 150.2, 134.0, 128.6, 126.5, 126.1, 122.0, 110.1, 79.7, 78.4, 57.5, 52.8, 47.6, 30.2, 28.8, 24.2.

##### 4.2.4.5 *tert*-Butyl (*S*)-3-((7-chloroquinolin-4-yl)amino)piperidine-1-carboxylate (**7a**)

Obtained following the general procedure B, starting from 4,7-dichloroquinoline (0.6 g, 3.03 mmol, 1 equiv) and (*S*)-1-Boc-3-aminopiperidine (2.43 g, 12.12 mmol, 4 equiv) as a brown oil (0.39 g, yield: 36%). Elution of the product with CHCl_3_/MeOH 50:1 (v/v). ^1^H NMR (400 MHz, DMSO-*d*_6_): δ 8.38 (d, *J* = 5.4 Hz, 1H), 8.31 (d, *J* = 9.1 Hz, 1H), 7.75 (d, *J* = 4.3 Hz, 1H), 7.42 (dd, *J* = 9.0, 2.3 Hz, 1H), 6.92 (d, *J* = 6.7 Hz, 1H), 6.54 (d, *J* = 5.5 Hz), 3.53–3.40 (m, 1H), 3.32 (m, 2H), 2.85 (m, *J* = 6.0 Hz, 2H), 1.99 (m, 1H), 1.79–1.61 (m, 2H), 1.36–1.33 (s, 9H), 1.27 (m, 2H).

##### 4.2.4.6 *tert*-Butyl (*R*)-3-((7-chloroquinolin-4-yl)amino)piperidine-1-carboxylate (**8a**)

Obtained following the general procedure B, starting from 4,7-dichloroquinoline (0.5 g, 2.52 mmol, 1 equiv) and (*R*)-*tert*-butyl 3-aminopiperidine-1-carboxylate (2.02 g, 10.1 mmol, 4 equiv) as a brown oil (0.28 g, yield: 31%). Elution of the product with CHCl_3_/MeOH 50:1 (v/v). ^1^H NMR (600 MHz, DMSO-*d*_6_): δ 8.43 (d, *J* = 5.1 Hz, 1H), 8.35 (d, *J* = 8.9 Hz, 1H), 7.80 (d, *J* = 2.2 Hz, 1H), 7.47 (dd, *J* = 9.0, 2.3 Hz, 1H), 6.97 (s, 1H), 6.59 (d, *J* = 5.2 Hz, 1H), 3.75 (m, 1H), 3.62 (m, 1H), 3.51 (m, 1H), 2.91 (m, 3H), 2.06 (m, 1H), 1.86–1.67 (m, 2H), 1.39 (s, 9H).

##### 4.2.4.7 *tert*-Butyl {2-[(7-fluoroquinolin-4-yl)amino]ethyl}carbamate (**19**)

Obtained following the general procedure B, starting from 4-chloro-7-fluoroquinoline (0.5 g, 2.75 mmol, 1 equiv) and *N*-Boc-ethylenediamine (2.21 g, 13.77 mmol, 2.18 mL, 5 equiv) as an orange oil (0.2 g, yield: 27%). ^1^H NMR (400 MHz, DMSO-*d*_6_): δ 8.35 (d, *J* = 5.4 Hz, 1H), 8.18 (dd, *J* = 9.3, 6.2 Hz, 1H), 7.42 (dd, *J* = 10.8, 2.7 Hz, 1H), 7.34–7.24 (m, 2H), 7.01 (t, *J* = 5.7 Hz, 1H), 6.46 (d, *J* = 5.5 Hz, 1H), 3.28 (dd, *J* = 12.0, 6.0 Hz, 2H), 3.17 (dd, *J* = 12.5, 6.3 Hz, 2H), 1.34 (s, 9H).

##### 4.2.4.8 4-Chloro-7-methylquinoline (**20**)

The product was obtained following the general procedure D. Starting from 3-methylaniline (3.0 g, 28.00 mmol, 1 equiv) and diethyl 2-ethoxymethylenemalonate (6.05 g, 28.00 mmol, 5.98 mL, 1 equiv), a yellow solid was obtained, which was carefully added in portions to Dowtherm (20 mL) and heated to 260 °C. A light orange solid was obtained upon cooling and filtration of the reaction mixture. The solid was then suspended in a 10% NaOH solution (20 mL) and heated to 110 °C for 3 h. Upon cooling to rt, the reaction mixture was acidified with sat. HCl and a large amount of solid precipitated. The solid was finally added to Dowtherm and heated to 240 °C for 1 h. The product precipitated as a mixture of two regioisomers, which were separated using column chromatography on silica gel using hexane–ethyl acetate 8:1–5:1 (v/v) yielding 7-methylquinolin-4-ol (major, 1.07 g) and 6-methylquinoline-4-ol (minor, 0.2 g). The major regioisomer (0.9 g) was then suspended in POCl_3_ and heated to reflux for 16 h. Upon workup and column chromatography with CHCl_3_/MeOH (30:1 v/v), 4-chloro-7-methylquinoline was obtained as a light brown solid (0.68 g, yield over four steps: 13%).

^1^H NMR (400 MHz, DMSO-*d*_6_): δ 8.73 (d, *J* = 4.7 Hz), 8.00 (d, *J* = 8.6 Hz), 7.83 (br s), 7.61 (d, *J* = 4.7 Hz), 7.52 (dd, *J* = 8.6, 1.6 Hz), 2.48 (s). ^13^C NMR (101 MHz, DMSO-*d*_6_): δ 151.0, 149.3, 141.4, 141.3, 130.7, 128.9, 124.1, 123.8, 121.3, 21.7.

##### 4.2.4.9 4-Chloro-7-phenoxyquinoline (**21**)

The product was obtained following the general procedure D. Starting from 3-phenoxyaniline (2.33 g, 12.58 mmol, 1 equiv) and diethyl 2-ethoxymethylenemalonate (2.72 g, 12.58 mmol, 2.69 mL, 1 equiv), a light yellow oil was obtained, which was carefully added in portions to Dowtherm (10 mL) and heated to 260 °C. An off-white solid was obtained upon cooling and filtration of the reaction mixture. The solid was then suspended in a 10% NaOH solution (10 mL) and heated to 110 °C for 3 h. Upon cooling to rt, the reaction mixture was acidified with sat. HCl and a large amount of an off-white solid precipitated. The solid was finally added to Dowtherm and heated to 240 °C for 1 h. The product precipitated as a light yellow powder, yielding 7-phenoxyquinolin-4-ol as a major regioisomer (1.28 g). The product (1.2 g) was then suspended in POCl_3_ and heated to reflux for 16 h. Upon workup and column chromatography with CHCl_3_/MeOH (80:1 v/v), 4-chloro-7-phenoxyquinoline was obtained as a light yellow oil (0.91 g, yield over four steps: 28%).

^1^H NMR (400 MHz, DMSO-*d*_6_): δ 8.72 (d, *J* = 4.8 Hz), 8.18 (d, *J* = 9.2 Hz), 7.62 (d, *J* = 4.7 Hz), 7.50 (dd, *J* = 9.2, 2.5 Hz), 7.48–7.43 (m), 7.29 (d, *J* = 2.5 Hz), 7.25 (t, *J* = 7.4 Hz), 7.19–7.14 (m). ^13^C NMR (101 MHz, DMSO-*d*_6_): δ 159.7, 155.6, 151.9, 150.4, 141.6, 131.0, 126.5, 125.5, 122.3, 121.7, 120.9, 120.7, 114.0.

##### 4.2.4.10 4-Chloro-7-methoxyquinoline (**22**)

7-Methoxy-4-quinolinol (2.0 g, 11.4 mmol, 1 equiv) was added slowly to POCl_3_ (8.86 g, 5.4 mL, 57.8 mmol, 5 equiv). The reaction was stirred at 110 °C for 16 h. Next, the reaction mixture was cooled to rt, and the excess of phosphorus oxychloride was evaporated. The solid was treated with an ice-cold 1 M solution of NaOH and extracted 5× to EtOAc. The organic layers were dried over sodium sulfate. The solvent was evaporated, yielding 1.8 g (yield: 81%) of 4-chloro-7-methoxyquinoline as a light orange solid.

^1^H NMR (400 MHz, DMSO-*d*_6_): δ 8.71 (d, *J* = 4.8 Hz, 1H), 8.03 (d, *J* = 9.2 Hz, 1H), 7.53 (d, *J* = 4.8 Hz, 1H), 7.42 (d, *J* = 2.5 Hz, 1H), 7.35 (dd, *J* = 9.2, 2.6 Hz, 1H), 3.90 (s, 3H). ^13^C NMR (101 MHz, DMSO-*d*_6_): δ 161.5, 151.4, 151.0, 141.4, 125.4, 121.3, 121.0, 119.9, 108.4, 56.3.

##### 4.2.4.11 4-Chloroquinoline-7-ol (**23**)

4-Chloro-7-methoxyquinoline (0.66 g, 3.4 mmol) was suspended in 48% HBr (5.1 mL) and Ac_2_O (3.4 mL). The reaction mixture was heated to 130 °C for 16 h. After cooling to rt, the mixture was neutralized with ice-cold sat. NaHCO_3_. The precipitated solid was filtered off. This gave 4-chloroquinoline-7-ol as a beige solid (0.37 g, yield: 60%).

^1^H NMR (600 MHz, DMSO-*d*_6_): δ 10.51 (s, 1H), 8.69 (d, *J* = 4.7 Hz, 1H), 8.05 (d, *J* = 9.4 Hz, 1H), 7.49 (d, *J* = 4.7 Hz, 1H), 7.35–7.30 (m, 2H). ^13^C NMR (151 MHz, DMSO-*d*_6_): δ 160.0, 151.1, 151.0, 141.4, 125.6, 121.2, 120.1, 119.1, 111.0.

##### 4.2.4.12 7-(Benzyloxy)-4-chloroquinoline (**24**)

4-Chloroquinoline-7-ol (0.36 g, 2.0 mmol, 1 equiv) was suspended in DMF (15 mL). The reaction mixture was cooled to 0 °C. Next, sodium hydride (60% dispersion in mineral oil, 0.2 g, 5.0 mmol, 2.5 equiv) was added portion-wise. The reaction mixture was stirred for 30 min. Then benzyl bromide (0.598 g, 3.5 mmol, 1.75 equiv) was added dropwise. After the completion of the reaction, the mixture was poured into water and extracted with ethyl acetate. The organic phase was dried over sodium sulfate, concentrated, and loaded onto silica gel. Elution with CHCl_3_/MeOH (100:1) yielded 7-(benzyloxy)-4-chloroquinoline as a yellow solid (0.39 g, yield: 72%).

##### 4.2.4.13 *tert*-Butyl {2-[(8-fluoroquinolin-4-yl)amino]ethyl}carbamate (**25**)

Obtained following the general procedure B, starting from 4-chloro-8-fluoroquinoline (0.5 g, 2.75 mmol, 1 equiv) and *N*-Boc-ethanediamine (2.03 g, 11.0 mmol, 1.76 mL, 4 equiv) as a white solid (0.84 mg, yield: 70%). Elution of the product with CHCl_3_/MeOH 40:1 (v/v).

^1^H NMR (600 MHz, DMSO-*d*_6_): δ 8.43 (d, *J* = 5.3 Hz, 1H), 7.95 (d, *J* = 8.3 Hz, 1H), 7.46–7.34 (m, 2H), 7.29 (t, *J* = 5.2 Hz, 1H), 7.06 (t, *J* = 5.8 Hz, 1H), 6.59 (d, *J* = 5.4 Hz, 1H), 3.38–3.32 (m, 3H), 3.26–3.17 (m, 2H), 1.38 (s, 9H).

##### 4.2.4.14 *N*^1^-(7-Chloroquinolin-4-yl)ethane-1,2-diamine (**F408**)

Obtained following the general procedure A, starting from 4,7-dichloroquinoline (0.5 g, 2.52 mmol, 1 equiv) and 1,2-ethanediamine (1.52 g, 25.25 mmol, 1.69 mL, 10 equiv) as an off-white solid (0.18 mg, yield: 63%). Elution of the product with DCM/MeOH 20:1 (v/v) +0.13 N NH_3_.

^1^H NMR (400 MHz, DMSO-*d*_6_): δ 8.39 (d, *J* = 5.3 Hz, 1H), 8.29 (d, *J* = 8.9 Hz, 1H), 7.78 (s, 1H), 7.44 (d, *J* = 8.4 Hz, 1H), 7.30–7.17 (m, 1H), 6.50–6.43 (m, 1H), 3.24 (s, 2H), 2.82 (t, *J* = 6.2 Hz, 2H), 1.62 (br s, 2H). ^13^C NMR (101 MHz, DMSO-*d*_6_): δ 151.9, 150.3, 149.1, 133.4, 127.5, 124.2, 124.0, 117.5, 98.7, 46.2. HRMS ESI–MS: found for [M + H^+^], 222.0790 *m*/*z* calcd, 222.07.

##### 4.2.4.15 2-((7-Chloroquinolin-4-yl)amino)ethan-1-ol (**1**)

Obtained following the general procedure A, starting from 4,7-dichloroquinoline (1.0 g, 5.04 mmol, 1 equiv) and ethanolamine (3.08 g, 50.4 mmol, 3.04 mL, 10 equiv) as an off-white solid (0.37 g, yield: 33%). Elution of the product with DCM/MeOH 15:1 (v/v).

^1^H NMR (600 MHz, DMSO-*d*_6_): δ 8.39 (d, *J* = 5.4 Hz, 1H), 8.26 (d, *J* = 9.0 Hz, 1H), 7.78 (d, *J* = 2.2 Hz, 1H), 7.44 (dd, *J* = 9.0, 2.3 Hz, 1H), 7.26 (t, *J* = 5.3 Hz, 1H), 6.50 (d, *J* = 5.4 Hz, 1H), 4.84 (t, *J* = 5.6 Hz, 1H), 3.66 (q, *J* = 5.9 Hz, 2H), 3.36 (q, *J* = 10.4, 4.6 Hz, 2H). ^13^C NMR (151 MHz, DMSO-*d*_6_): δ 151.9, 150.2, 149.1, 133.4, 127.5, 124.1, 117.5, 98.7, 58.8, 45.1. HRMS ESI–MS: found for [M + H^+^], 223.0630 *m*/*z* calcd, 223.06.

##### 4.2.4.16 *N*^1^-(7-Chloroquinolin-4-yl)-*N*^2^,*N*^2^-dimethylethane-1,2-diamine (**2**)

Obtained following the general procedure A, starting from 4,7-dichloroquinoline (1.0 g, 5.05 mmol, 1 equiv) and *N*,*N*-dimethylethylenediamine (4.45 g, 50.5 mmol, 5.51 mL, 10 equiv) as an orange solid (0.74 g, yield: 58%). Elution of the product with DCM/MeOH 20:1 (v/v).

^1^H NMR (600 MHz, DMSO-*d*_6_): δ 8.40 (d, *J* = 5.4 Hz, 1H), 8.22 (d, *J* = 9.0 Hz, 1H), 7.79 (d, *J* = 2.2 Hz, 1H), 7.45 (dd, *J* = 9.0, 2.2 Hz, 1H), 7.20 (t, *J* = 5.2 Hz, 1H), 6.49 (d, *J* = 5.4 Hz, 1H), 3.35 (q, 2H, partially overlapped with water peak), 2.54 (t, *J* = 6.8 Hz, 2H), 2.22 (s, 6H). ^13^C NMR (101 MHz, DMSO-*d*_6_) NMR (101 MHz): δ 151.9, 150.0, 149.1, 133.4, 127.5, 124.1, 124.0, 117.4, 98.7, 56.9, 45.3, 40.5. HRMS ESI–MS: found for [M + H^+^], 250.1111 *m*/*z* calcd, 250.11.

##### 4.2.4.17 (*S*)-1-(7-Chloroquinolin-4-yl)pyrrolidin-3-amine (**3**)

Obtained following the general procedure C, starting from **3a** (1.2 g, 3.71 mmol, 1 equiv) and 4 M HCl in dioxane (1.85 g, 37.11 mmol, 9.28 mL, 10 equiv) as a white solid (0.21 g, yield: 23%). Elution of the product with DCM/MeOH 15:1 (v/v).

^1^H NMR (400 MHz, DMSO-*d*_6_): δ 8.36 (d, *J* = 5.5 Hz, 1H), 8.27 (d, *J* = 9.2 Hz, 1H), 7.80 (d, *J* = 2.2 Hz, 1H), 7.34 (dd, *J* = 9.2, 2.2 Hz, 1H), 6.47 (d, *J* = 5.6 Hz, 1H), 3.81–3.74 (m, 2H), 3.61–3.55 (m, 2H), 3.35–3.31 (m, 1H), 2.08–2.00 (m, 1H), 1.80–1.65 (m, 2H). ^13^C NMR (101 MHz, DMSO-*d*_6_): δ 151.7, 150.8, 150.7, 132.9, 127.6, 127.5, 122.9, 118.9, 102.8, 60.3, 50.89, 50.0, 33.9. HRMS ESI–MS: found for [M + H^+^], 248.0950 *m*/*z* calcd, 248.09.

##### 4.2.4.18 (*R*)-1-(7-Chloroquinolin-4-yl)pyrrolidin-3-amine (**4**)

Obtained following the general procedure C, starting from **4a** (0.34 g, 0.98 mmol, 1 equiv) and 4 M HCl in dioxane (0.36 g, 9.77 mmol, 2.42 mL, 10 equiv) as an off-white solid (0.14 g, yield: 58%). Elution of the product with DCM/MeOH 15:1 (v/v).

^1^H NMR (400 MHz, DMSO-*d*_6_): δ 8.32 (d, *J* = 5.5 Hz, 1H), 8.23 (d, *J* = 9.2 Hz, 1H), 7.76 (d, *J* = 2.3 Hz, 1H), 7.30 (dd, *J* = 9.2, 2.4 Hz, 1H), 6.43 (d, *J* = 5.6 Hz, 1H), 3.78–3.65 (m, 2H), 3.60–3.49 (m, 2H), 3.35–3.24 (m, 2H), 2.08–1.91 (m, 1H), 1.78–1.63 (m, 2H). ^13^C NMR (101 MHz, DMSO-*d*_6_): δ 152.2, 151.3, 151.2, 133.4, 128.1, 128.0, 123.5, 119.5, 103.3, 60.8, 51.4, 50.5, 34.4. HRMS ESI–MS: found for [M + H^+^], 248.0949 *m*/*z* calcd, 248.09.

##### 4.2.4.19 (3*S*)-1-(7-Chloroquinolin-4-yl)piperidin-3-amine (**5**)

Obtained following the general procedure C, starting from **5a** (0.17 g, 0.47 mmol, 1 equiv) and 4 M HCl in dioxane (0.317 g, 4.70 mmol, 1.17 mL, 10 equiv) as a transparent oil (0.07 g, yield: 56%). Elution of the product with DCM/MeOH 20:1 (v/v) +0.13 N NH_3_.

^1^H NMR (400 MHz, DMSO-*d*_6_): δ 8.62 (d, *J* = 5.0 Hz, 1H), 7.99 (d, *J* = 9.0 Hz, 1H), 7.91 (d, *J* = 2.2 Hz, 1H), 7.49 (dd, *J* = 9.0, 2.2 Hz, 1H), 6.90 (d, *J* = 5.1 Hz, 1H), 3.42–3.34 (m, 1H), 3.29–3.26 (m, 1H), 2.98–2.92 (m, 1H), 2.80–2.71 (m, 3H), 2.55–2.50 (m, 1H), 1.93–1.84 (m, 1H), 1.84–1.76 (m, 1H), 1.74–1.64 (m, 1H), 1.24–1.15 (m, 1H). ^13^C NMR (101 MHz, DMSO-*d*_6_): δ 157.3, 152.7, 150.2, 134.0, 128.5, 126.7, 126.0, 122.1, 109.9, 61.3, 52.6, 48.3, 33.8, 24.1.

##### 4.2.4.20 (3*R*)-1-(7-Chloroquinolin-4-yl)piperidin-3-amine (**6**)

Obtained following the general procedure C, starting from **6a** (0.39 g, 1.07 mmol, 1 equiv) and 4 M HCl in dioxane (0.39 g, 10.69 mmol, 2.67 mL, 10 equiv) as a transparent oil (0.14 g, yield: 58%). Elution of the product with DCM/MeOH 20:1 (v/v) +0.13 N NH_3_.

^1^H NMR (400 MHz, DMSO-*d*_6_): δ 8.66 (d, *J* = 5.0 Hz, 1H), 8.03 (d, *J* = 9.0 Hz, 1H), 7.95 (d, *J* = 2.2 Hz, 1H), 7.53 (dd, *J* = 9.0, 2.2 Hz, 1H), 6.95 (s, 1H), 3.44–3.39 (m, 1H), 3.34–3.30 (m, 1H), 2.99–2.94 (m, 1H), 2.82–2.76 (m, 1H), 2.58–2.52 (m, 1H), 1.94–1.88 (m, 1H), 1.86–1.80 (m, 1H), 1.77–1.69 (m, 1H), 1.27–1.18 (m, 1H). ^13^C NMR: δ 156.8, 152.2, 149.7, 133.5, 128.0, 126.2, 125.5, 121.6, 109.4, 61.1, 52.1, 47.9, 33.5, 23.7. HRMS ESI–MS: found for [M + H^+^], 262.1110 *m*/*z* calcd, 262.1105.

##### 4.2.4.21 (*S*)-7-Chloro-*N*-(piperidin-3-yl)quinolin-4-amine (**7**)

Obtained following the general procedure C, starting from **7a** (0.32 g, 0.9 mmol, 1 equiv) and 4 M HCl in dioxane (0.33 g, 8.95 mmol, 2.24 mL, 10 equiv) as an off-white solid (0.1 g, yield: 43%). Elution of the product with DCM/MeOH 20:1 (v/v) +0.13 N NH_3_.

^1^H NMR (600 MHz, DMSO-*d*_6_): δ 8.39 (d, *J* = 5.4 Hz, 1H), 8.34 (d, *J* = 9.0 Hz, 1H), 7.78 (d, *J* = 2.2 Hz, 1H), 7.43 (dd, *J* = 9.0, 2.3 Hz, 1H), 6.86 (d, *J* = 7.7 Hz, 1H), 6.53 (t, *J* = 6.5 Hz, 1H), 3.56–3.49 (m, 1H), 3.13 (dd, *J* = 12.0, 2.2 Hz, 1H), 2.86 (dd, *J* = 8.9, 3.5 Hz, 1H), 2.48–2.44 (m, 2H), 1.99 (d, *J* = 9.1 Hz, 1H), 1.70–1.65 (m, 1H), 1.61–1.55 (m, 1H), 1.52–1.45 (m, 1H). ^13^C NMR (101 MHz, DMSO-*d*_6_): δ 152.0, 149.3, 149.2, 133.4, 127.5, 124.4, 123.9, 117.5, 99.0, 50.5, 49.5, 45.8, 30.1, 25.3. HRMS ESI–MS: found for [M + H^+^], 262.1110 *m*/*z* calcd, 262.11.

##### 4.2.4.22 (*R*)-7-Chloro-*N*-(piperidin-3-yl)quinolin-4-amine (**8**)

Obtained following the general procedure C, starting from **8a** (0.27 g, 0.75 mmol, 1 equiv) and 4 M HCl in dioxane (0.27 g, 7.46 mmol, 1.87 mL, 10 equiv) as an off-white solid (0.1 g, yield: 51%). Elution of the product with DCM/MeOH 20:1 (v/v) +0.13 N NH_3_. ^1^H NMR (400 MHz, DMSO-*d*_6_): δ 8.39 (d, *J* = 5.4 Hz, 1H), 8.35 (d, *J* = 9.1 Hz, 1H), 7.78 (d, *J* = 2.2 Hz, 1H), 7.44 (dd, *J* = 9.0, 2.2 Hz, 1H), 6.88 (d, *J* = 7.7 Hz, 1H), 6.54 (d, *J* = 5.6 Hz, 1H), 3.58–3.51 (m, 1H), 3.17–3.10 (m, 1H), 2.89–2.82 (m, 1H), 2.50–2.41 (m, 1H), 2.02–1.97 (m, 1H), 1.71–1.65 (m, 1H), 1.63–1.54 (m, 1H), 1.54–1.47 (m, 1H). ^13^NMR (101 MHz, DMSO-*d*_6_): δ 152.0, 149.3, 149.2, 133.4, 127.5, 124.4, 123.9, 117.5, 99.0, 50.4, 49.4, 45.7, 30.1, 25.2. HRMS ESI–MS: found for [M + H^+^], 262.1109 *m*/*z* calcd, 262.1105.

##### 4.2.4.23 *N*^1^-(7-(Trifluoromethyl)quinolin-4-yl)ethane-1,2-diamine (**9**)

Obtained following the general procedure A, starting from 4-chloro-7-(trifluoromethyl)quinoline (1.0 g, 4.32 mmol, 1 equiv) and 1,2-ethanediamine (1.3 g, 21.6 mmol, 1.5 mL, 5 equiv) as an off-white solid (0.46 g, yield: 56%). Elution of the product with DCM/MeOH 25:1 (v/v).

^1^H NMR (400 MHz, DMSO-*d*_6_): δ 8.50 (d, *J* = 5.6 Hz, 2H), 8.08 (s, 1H), 7.70–7.65 (m, 1H), 7.42 (s, 1H), 6.61 (d, *J* = 5.4 Hz, 1H), 3.30 (dd, *J* = 9.9, 5.3 Hz, 2H), 2.86 (t, *J* = 6.4 Hz, 2H), 2.30 (br s, 2H). ^13^C NMR (101 MHz, DMSO-*d*_6_): δ 152.3, 150.2, 147.5, 129.5, 129.2, 128.9, 128.6, 126.4 (q, *J* = 4.2 Hz), 125.6, 124.0, 122.9, 120.93, 118.8, 99.8, 46.0. HRMS ESI–MS: found for [M + H^+^], 256.1054 *m*/*z* calcd, 256.10.

##### 4.2.4.24 *N*^1^-(7-Methylquinolin-4-yl)ethane-1,2-diamine (**10**)

Obtained following the general procedure A, starting from **20** (0.5 g, 2.81 mmol, 1 equiv) and 1,2-ethanediamine (1.69 g, 28.15 mmol, 1.67 mL, 10 equiv) as an off-white solid (0.19 g, yield: 34%). Elution of the product with DCM/MeOH 25:1 (v/v) +0.13 N NH_3_.

^1^H NMR (400 MHz, DMSO-*d*_6_): δ 8.33 (d, *J* = 5.3 Hz, 1H), 8.11 (d, *J* = 8.5 Hz, 1H), 7.56 (s, 1H), 7.24 (d, *J* = 8.5 Hz, 1H), 7.01 (s, 1H), 6.39 (d, *J* = 5.4 Hz, 1H), 3.26–3.21 (m, 2H), 2.82 (t, *J* = 6.5 Hz, 2H), 2.44 (s, 3H). ^13^C NMR (101 MHz, DMSO-*d*_6_): δ 150.7, 150.0, 148.6, 138.2, 128.1, 125.7, 121.5, 116.8, 97.8, 46.1, 40.2, 21.1. HRMS ESI–MS: found for [M + H^+^], 202.1340 *m*/*z* calcd, 202.13.

##### 4.2.4.25 *N*^1^-(7-Bromoquinolin-4-yl)ethane-1,2-diamine (**11**)

Obtained following the general procedure A, starting from 7-bromo-4-chloroquinoline (0.5 g, 2.06 mmol, 1 equiv) and 1,2-ethanediamine (1.38 g, 20.62 mmol, 1.38 mL, 10 equiv) as an orange solid (0.51 g, yield: 93%). Elution of the product with DCM/MeOH 30:1 (v/v).

^1^H NMR (400 MHz, DMSO-*d*_6_): δ 8.34 (d, *J* = 5.4 Hz, 1H), 8.17 (d, *J* = 9.0 Hz, 1H), 7.90 (d, *J* = 2.1 Hz, 1H), 7.51 (dd, *J* = 9.0, 2.1 Hz, 1H), 7.21 (t, *J* = 4.6 Hz, 1H), 6.45 (d, *J* = 5.5 Hz, 1H), 3.21 (q, *J* = 11.6, 6.2 Hz, 2H), 2.78 (t, *J* = 6.5 Hz, 2H), 1.63 (br s, 2H). ^13^C NMR (101 MHz, DMSO-*d*_6_): δ 152.4, 150.9, 149.9, 131.3, 127.0, 124.7, 122.6, 118.2, 99.3, 46.7. HRMS ESI–MS: found for [M + H^+^], 266.0289 *m*/*z* calcd, 266.0287.

##### 4.2.4.26 *N*^1^-(Quinolin-4-yl)ethane-1,2-diamine (**12**)

Obtained following the general procedure A, starting from 4-chloroquinoline (0.1 g, 0.61 mmol, 1 equiv) and 1,2-ethanediamine (0.37 g, 6.11 mmol, 0.41 mL, 10 equiv) as a yellow solid (0.082 g, yield: 50%). Elution of the product with DCM/MeOH 20:1 (v/v) +0.13 N NH_3_.

^1^H NMR (400 MHz, DMSO-*d*_6_): δ 8.38 (d, *J* = 5.3 Hz, 1H), 8.23 (d, *J* = 8.3 Hz, 1H), 7.77 (d, *J* = 8.3 Hz, 1H), 7.59 (t, *J* = 7.3 Hz, 1H), 7.40 (t, *J* = 7.4 Hz, 1H), 7.15 (s, 1H), 6.47 (d, *J* = 5.4 Hz, 1H), 3.29 (t, *J* = 6.3 Hz, 2H), 2.86 (t, *J* = 6.4 Hz, 2H). ^13^C NMR (101 MHz, DMSO-*d*_6_): δ 150.7, 150.1, 148.3, 129.0, 128.7, 123.8, 121.8, 118.9, 98.3, 45.5. HRMS ESI–MS: found for [M + H^+^], 188.1179 *m*/*z* calcd, 188.1182.

##### 4.2.4.27 *N*^1^-(7-Fluoroquinolin-4-yl)ethane-1,2-diamine (**13**)

Obtained following the general procedure B, starting from **19** (0.2 g, 0.65 mmol, 1 equiv) and 4 M HCl in dioxane (0.24 g, 6.55 mmol, 1.64 mL, 10 equiv) as a yellow solid (0.053 g, yield: 39%). Elution of the product with DCM/MeOH 20:1 (v/v) +0.13 N NH_3_.

^1^H NMR (600 MHz, DMSO-*d*_6_): δ 8.42 (d, *J* = 5.4 Hz, 1H), 8.38 (dd, *J* = 9.2, 6.3 Hz, 1H), 7.50 (dd, *J* = 10.8, 2.7 Hz, 1H), 7.37 (td, *J* = 9.0, 2.7 Hz, 1H), 7.32 (s, 1H), 6.51 (d, *J* = 5.4 Hz, 1H), 3.32 (m, 2H), 2.88 (t, *J* = 6.4 Hz, 2H). ^13^C NMR (151 MHz, DMSO-*d*_6_): δ 163.4, 161.8, 152.4, 150.8, 150.2, 125.1, 116.4, 113.8, 113.6, 112.6, 112.5, 98.6, 46.3. HRMS ESI–MS: found for [M + H^+^], 222.0795.1054 *m*/*z* calcd, 222.08.

##### 4.2.4.28 *N*^1^-(7-Methoxyquinolin-4-yl)ethane-1,2-diamine (**14**)

Obtained following the general procedure A, starting from **22** (0.7 g, 3.62 mmol, 1 equiv) and 1,2-ethanediamine (2.17 g, 36.15 mmol, 2.15 mL, 10 equiv) as an off-white solid (0.374 g, yield: 48%). Elution of the product with DCM/MeOH 20:1 (v/v) +0.13 N NH_3_.

^1^H NMR (600 MHz, DMSO-*d*_6_): δ 8.31 (d, *J* = 5.4 Hz, 1H), 8.14 (d, *J* = 9.2 Hz, 1H), 7.16 (d, *J* = 2.6 Hz, 1H), 7.07–6.98 (m, 2H), 6.36 (d, *J* = 5.4 Hz, 1H), 3.87 (s, 3H), 3.24 (m, 2H), 2.83 (t, *J* = 6.5 Hz, 2H). ^13^C NMR (151 MHz, DMSO-*d*_6_): δ 160.0, 151.5, 150.7, 123.6, 115.9, 113.8, 108.3, 97.7, 55.6, 49.1, 46.4, 40.7. HRMS ESI–MS: found for [M + H^+^], 218.1292 *m*/*z* calcd, 218.13.

##### 4.2.4.29 *N*^1^-(7-Phenoxyquinolin-4-yl)ethane-1,2-diamine (**15**)

Obtained following the general procedure A, starting from **21** (0.6 g, 2.35 mmol, 1 equiv) and 1,2-ethanediamine (1.41 g, 23.47 mmol, 1.57 mL, 10 equiv) as a yellow powder (0.25 g, yield: 38%). Elution of the product with DCM/MeOH 40:1 (v/v).

^1^H NMR (400 MHz, DMSO-*d*_6_): δ 8.32–8.25 (m, 2H), 7.47–7.42 (m, 2H), 7.23–7.08 (m, 6H), 6.40 (d, *J* = 5.5 Hz, 1H), 3.28–3.22 (m, 2H), 2.82 (t, *J* = 6.5 Hz, 2H). ^13^C NMR (101 MHz, DMSO-*d*_6_): δ 157.5, 156.0, 151.5, 150.3, 149.8, 130.2, 124.1, 124.1, 119.52, 116.5, 115.1, 114.2, 97.8, 45.9. HRMS ESI–MS: found for [M + H^+^], 280.1445 *m*/*z* calcd, 280.1444.

##### 4.2.4.30 *N*^1^-[7-(Benzyloxy)quinolin-4-yl]ethane-1,2-diamine (**16**)

Obtained following the general procedure A, starting from 4,8-dichloroquinoline (0.54 g, 2.73 mmol, 1 equiv) and 1,2-ethanediamine (0.82 g, 13.63 mmol, 0.85 mL, 5 equiv) as an off-white solid (0.31 g, yield: 51%). Elution of the product with DCM/MeOH 40:1 (v/v).

^1^H NMR (600 MHz, DMSO-*d*_6_): δ 8.26 (d, *J* = 5.4 Hz, 1H), 8.12 (d, *J* = 9.2 Hz, 1H), 7.48–7.44 (m, 2H), 7.37 (dd, *J* = 11.4, 4.3 Hz, 2H), 7.32–7.26 (m, 1H), 7.21 (d, *J* = 2.6 Hz, 1H), 7.08 (dd, *J* = 9.2, 2.6 Hz, 1H), 6.32 (d, *J* = 5.5 Hz, 1H), 5.18 (s, 2H), 3.22 (t, *J* = 6.4 Hz, 2H), 2.80 (t, *J* = 6.4 Hz, 2H). ^13^C NMR (101 MHz, DMSO-*d*_6_): δ 159.1, 151.5, 150.7, 150.6, 137.5, 129.0, 128.4, 128.3, 123.7, 116.4, 114.0, 109.6, 97.8, 69.7, 46.0. HRMS ESI–MS: found for [M + H^+^], 280.1445 *m*/*z* calcd, 280.1444.

##### 4.2.4.31 *N*^1^-(8-Chloroquinolin-4-yl)ethane-1,2-diamine (**17**)

Obtained following the general procedure A, starting from 4,8-dichloroquinoline (0.54 g, 2.73 mmol, 1 equiv) and 1,2-ethanediamine (0.82 g, 13.63 mmol, 0.85 mL, 5 equiv) as an off-white solid (0.31 g, yield: 51%). Elution of the product with DCM/MeOH 20:1 (v/v) +0.13 N NH_3_.

^1^H NMR (600 MHz, DMSO-*d*_6_): δ 8.46 (d, *J* = 5.4 Hz, 1H), 8.23 (dd, *J* = 8.5, 1.1 Hz, 1H), 7.78 (dd, *J* = 7.4, 1.1 Hz, 1H), 7.38–7.34 (m, 1H), 7.26 (t, *J* = 4.9 Hz, 1H), 6.56 (d, *J* = 5.4 Hz, 1H), 3.27 (dd, *J* = 11.9, 6.3 Hz, 2H), 2.82 (t, *J* = 6.5 Hz, 2H), 1.49 (br s, 2H). ^13^C NMR (151 MHz, DMSO-*d*_6_): δ 151.2, 150.6, 144.4, 132.55, 129.04, 123.6, 121.3, 120.2, 99.1, 45.6. HRMS ESI–MS: found for [M + H^+^], 222.0792 *m*/*z* calcd, 222.08.

##### 4.2.4.32 *N*^1^-(8-Fluoroquinolin-4-yl)ethane-1,2-diamine (**18**)

Obtained following the general procedure B, starting from **25** (0.2 g, 0.65 mmol, 1 equiv) and 4 M HCl in dioxane (0.24 g, 6.55 mmol, 1.64 mL, 10 equiv) as a white solid (0.085 g, yield: 63%). Elution of the product with DCM/MeOH 20:1 (v/v) +0.13 N NH_3_.

^1^H NMR (400 MHz, DMSO-*d*_6_): δ 8.40 (d, *J* = 5.3 Hz, 1H), 8.05 (d, *J* = 8.2 Hz, 1H), 7.44–7.34 (m, 2H), 7.22 (t, *J* = 4.0 Hz, 1H), 6.54 (d, *J* = 5.4 Hz, 1H), 3.26 (dd, *J* = 11.9, 6.2 Hz, 2H), 2.82 (t, *J* = 6.5 Hz, 2H), 1.57 (br s, 2H). ^13^C NMR (151 MHz, DMSO-*d*_6_): δ 158.5, 156.9, 150.8, 149.9, 138.5 (d, *J* = 11.5 Hz), 123.2 (d, *J* = 8.2 Hz), 120.8, 117.9, 113.06 (d, *J* = 18.8 Hz), 99.1, 44.2. HRMS ESI–MS: found for [M + H^+^], 206.1085.1054 *m*/*z* calcd, 206.11.

### 4.3 In Silico Methods

#### 4.3.1 Homology Modeling—Construction of the KIND2/FSI Complex Homology Model

A homology model for KIND2 bound to the FSI tail of FMN2 was constructed in Swiss-Model^20^ using a complex of KIND1/FSI as a template (PDB: 2YLE). The KIND2 sequence (22-203) was taken from the UniProt database (UniProt ID: Q8WWL2). The resulting homology model of the KIND2 was aligned with the FSI peptide in PyMol and saved as a PDB file. Both homologues of the KIND share 46% sequence identity (Figure S5).

#### 4.3.2 Protein Preparation for Molecular Docking

Before the molecular docking simulations, both KIND1 and KIND2 were investigated and preprocessed using Maestro (ver. 12.5.139; Schrödinger Release 2020-3: Maestro, Schrödinger, LLC, New York). The hydrogen atoms were added in an idealized position. The FSI peptide originally bound to KIND1 (PDB ID: 2YLE) and water molecules were removed for the molecular docking experiment.

#### 4.3.3 Molecular Docking in GOLD

The experiments were performed using GOLD 2021.3.0 (genetic optimization for ligand docking) software.^21^ The binding region was defined in the area corresponding to the location of the native FSI peptide ligand and set at the position of Asp158 (amino acid numerical label according to the original data for the crystal structure 2yle; in the KIND1 renumbered structure and KIND2 model, the docking center position was assigned at Asp119), including all atoms within 15 Å. During the semiflexible docking process, ligands were allowed flexibility to find the most probable binding pose using a genetic algorithm (GA) implemented in GOLD. The empirical ChemPLP scoring function was applied to evaluate the obtained results. Ten top-scored results were recorded for each docked molecule.

#### 4.3.4 Ligand Preparation

Ligand structures were prepared and minimized in the VEGA ZZ software.^22^ The Ammp conformational search was done by using the Boltzmann Jump method with 5000 steps. Flexible torsions were selected. The lowest energy conformation of the ligand was saved in.pdb format. Each fragment was then prepared in AutoDock Tools^23^ and saved as a .pdbqt file.

#### 4.3.5 Molecular Docking with Vina

The docking of fragments was performed in Vina.^24,25^ The center of the grid box (20 × 20 × 20) was placed using the following coordinates: center_*x* = 13.861, center_*y* = 42.405, and center_*z* = 19.361. All graphics were produced using PyMol visualization software.^26^

#### 4.3.6 Electrostatic Surface Representation

The electrostatic surface representation of the KIND2 was calculated using the APBS (Adaptive Poisson–Boltzmann Solver)^27^ plugin in PyMOL.

### 4.4 Protein Expression and Purification

Constructs encoding His6-KIND1, His6-KIND2, and His6-KIND2 Y106A were transformed in *E. coli* BL21 (DE3) and cultured in either LB or ^15^N-labeled M9 medium with ampicillin selection (100 μg/mL). ^15^N/^13^C-labeled construct of KIND2 (for backbone assignment) was cultured in M9 medium containing ^15^NH_4_Cl (5 g/L) and ^13^C-glucose (2 g/L) as sole sources of nitrogen and carbon, respectively. For all constructs, protein expression was induced with 0.3 mM isopropyl-β-d-thiogalactopyranoside (IPTG) when OD600 reached a 0.4–0.6 value. Proteins were expressed for 16–20 h at 20 °C. Cells were harvested by centrifugation (30 min, 2000 rpm, 4 °C), and bacterial pellets were frozen at −20 °C. Pellets were handled on ice using ice-cold buffers. Before protein isolation, pellets were thawed on ice and resuspended in ca. 40 mL of lysis buffer (25 mM Tris, pH 8.0, 200 mM NaCl, 25 mM imidazole, and 10 mM β-mercaptoethanol). Cells were lysed by ultrasonication, and the whole lysate was centrifuged at 20,000 rpm for 30 min at 4 °C. The resulting supernatant containing soluble proteins was loaded at a flow rate of 0.75 mL/min on the HiPrep IMAC FF 16/10 column equilibrated with a buffer containing 25 mM Tris, pH 8.0, 200 mM NaCl, 25 mM imidazole, and 5 mM β-mercaptoethanol. The column was then washed with buffer containing 25 mM Tris, pH 8.0, 300 mM NaCl, 25 mM imidazole, and 5 mM β-mercaptoethanol. Proteins (His6-KIND1, His6-KIND2, or His6-KIND2 Y106A) were eluted from the column with the elution buffer (25 mM Tris, pH 8.0, 300 mM NaCl, 200 mM imidazole, and 5 mM β-mercaptoethanol). For His6 tag removal, proteins were then cleaved with 10 U of TEV protease (Sigma-Aldrich) for 2 days. Proteins without a His6 tag were separated from their tagged counterparts by passing the solution through the IMAC column. Proteins were then dialyzed overnight against 20 mM HEPES, pH 7.5 buffer, and purified using a strong anion exchange column (Q HiTrap, GE Healthcare). KIND1 was eluted using a 0–60% gradient of buffer containing 1 M NaCl over 10 CV, whereas KIND2 was eluted using a 0–70% gradient over 10 CV of the same buffer. Finally, proteins were purified by a gel filtration method using an S75 Superdex column (GE Healthcare) equilibrated with storage buffer A (25 mM Tris–HCl, pH 7.8, 100 mM NaCl, 2.0 mM DTT). His6-tagged proteins were purified by a gel filtartion method using S75 Superdex column (GE Healthcare) equilibrated with storage buffer B (20 mM HEPES buffer pH 7.8, 100 mM NaCl, 2 mM DTT).

### 4.5 Differential Scanning Fluorimetry

Thermal melting experiments were carried out using a CFX96TM real-time PCR machine (BioRad). Protein thermal unfolding was monitored by the increase in the fluorescence of the SYPRO Orange dye (Sigma-Aldrich). Excitation and emission filters for the SYPRO Orange dye were set to 465 and 590 nm, respectively. The KIND1 or KIND2 were prepared in the storage buffer as 50 μM stock solutions. Ten μL of protein was then mixed with 2 μL of SYPRO Orange 45× and 10 μL of the 4 mM solution of tested compounds or 10 μL of the 8% DMSO solution in the protein storage buffer. The resulting mixture was finally buffered with 18 μL of protein storage buffer. A total reaction volume of 40 μL contained 12.5 μM protein, 2.25× Sypro Orange, and 1 mM tested compound. The heating gradient started at 25 °C, and the temperature was increased by 0.2 °C/10 s until 95 °C, and fluorescence readings of SYPRO Orange were taken at each interval. Raw data files with temperatures and their corresponding fluorescence values (or first derivative of the fluorescence – d*F*/d*T*) were exported to Microsoft Office Excel, where melting temperatures *T*_m_ were calculated from the lowest point of the first derivative plot. The visualization of melting curves was performed with GraphPad Prism software. Thermal shift values (Δ*T*_m_) were obtained through subtraction of the unfolding temperature of the KIND1 or KIND2 in the presence of 2% (vol/vol) DMSO (*T*_mDMSO_) from the unfolding temperatures of the KIND1 or KIND2 in the presence of fragment (*T*_mfrag_)
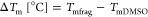


### 4.6 FP Assay

The FP competition assays were carried out in flat black-bottom 96-well plates using FITC-Ahx-GKSLYKIKPRHDSGIKAKISMKT-OH (Biomatik) as a reporter probe. The probe was diluted in the buffer assay (25 mM Tris, pH 7.8, 100 mM NaCl, 2 mM DTT) and added to the preplated mixtures of protein/fragment solutions. The protein concentration in the competition assays was fixed (1.17 and 2.49 μM for KIND1 and KIND2, respectively). Fragments were tested in serial dilutions ranging from 2.44 to 2500 μM. The change in mP values was measured and used to calculate an IC_50_ (the inhibitor concentration at which 50% of the bound probe is displaced) by fitting the mP data using GraphPad Prism software to a three-parameter dose–response equation. Each experiment was performed in triplicate with four technical replications. IC_50_ values obtained in the competition assays were used to calculate ligand efficiency (LE) according to the following equation for a temperature of 298 K
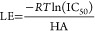
with the temperature *T* [K], the gas constant *R* [kcal mol^–1^ K^–1^], and the number of non-hydrogen atoms HA.^28^

### 4.7 Microscale Thermophoresis

His6-KIND1, His6-KIND2, or His6-KIND2 Y106A were labeled using the His-Tag Labeling Kit RED-tris-NTA second generation according to the manufacturer’s protocol.^29^ Proteins were buffered in 20 mM HEPES buffer pH 7.8, 100 mM NaCl, 2 mM DTT, and 0.05% Tween-20. Each fragment was 2× fold diluted, starting from 1.25 mM as the highest concentration; the final protein concentration was 37.5 nM. Samples were centrifuged at 10,000 rpm (Eppendorf Centrifuge 5415R) before loading into standard capillaries (Nanotemper Technologies, GmbH). Experiments were run on a Nanotemper MonolithNT.115 instrument (Nanotemper Technologies, GmbH) in triplicate at 40% LED intensity and 40% MST power. Quantification of fragment–protein interactions was done in GraphPad Prism software.

### 4.8 NMR Experiments

All NMR acquisition was performed in 3 mm tubes at 298 K with proteins buffered in 25 mM Na-phosphate buffer, pH 7.8, 2 mM DTT, and 10% (v/v) D_2_O for deuterium lock. Titration NMR experiments were performed on Bruker Avance III spectrometers operating at 600 MHz 1H frequency using a H/N/C/F quadruple-resonance cryogenic probe. Water suppression was carried out using the WATERGATE sequence. ^1^H–^15^N heteronuclear correlations were obtained by using the fast HSQC pulse sequence. Spectra were processed and analyzed using Mnova Binding 14.0 software. Titration experiments were carried out using protein concentrations of 0.3 mM (KIND1), 0.28 or 0.05 mM (KIND2), and 0.2 mM (KIND2 Y106A), and were used to quantify the binding affinity of hit **F408** and compound **13**. All samples were buffered in 25 mM Na-phosphate buffer, pH 7.8, and 2 mM DTT containing 10% (v/v) D_2_O for deuterium lock. The change in chemical shifts of backbone resonances upon stepwise addition of the fragment was automatically calculated using the Mnova Binding plug-in.

For backbone assignment, 0.2 mM ^15^N,^13^C-labeled KIND2 was measured on Bruker Avance III spectrometers operating at 600 MHz or 1.2 GHz ^1^H frequencies using the H/N/C/F quadruple-resonance cryogenic probe or the H/N/C triple-resonance rt probe, respectively. Three-dimensional HNCO, HNCACO, HNCACB, and CBCACONH experiments were acquired with nonuniform sampling. Spectra were processed using Topspin 3.5 (Bruker) or NMRpipe^30^ and analyzed with CCPN 3.1.0. About 40% of the backbone resonance frequencies could be assigned.

## Supporting Information Available

Fragment-based screening data, establishment of fluorescence polarization assay, evaluation of binding of F408 to KIND2 Y106A domain, spectrum of KIND2 with assigned backbone resonances, titrations data of compound 13, comparison of KIND1 and KIND2 sequences, ^1^H and ^13^C spectra of tested fragments, and LC–MS chromatograms of tested fragments (PDF)
Molecular formula strings of compounds (CSV)
Homology model of KIND2/FSI complex (PDB)
Binding mode of 13 to KIND2 model (PDB)

## Supplementary Material

jm3c00877_si_002.csv

jm3c00877_si_003.pdb

jm3c00877_si_004.pdb

## Abbreviations

ATP: adenosine triphosphate
CSP: chemical shift perturbation
DCM: dichloromethane
DIPEA: diisopropylethylamine
DSF: differential scanning fluorimetry
FITC: fluorescein isothiocyanate
FP: fluorescence polarization
HAC: heavy atom count
KIND: kinase noncatalytic C-lobe domain
LE: ligand efficiency
MW: molecular weight
PDB: protein data bank
SAR: structure–activity relationship
