# Supplementary material for: Discovery of Inhibitory Fragments That Selectively Target Spire2–FMN2 Interaction
Source: J Med Chem. 2023 Dec 1;66(23):15715–27. doi: 10.1021/acs.jmedchem.3c00877 (PMC10726347; doi:10.1021/acs.jmedchem.3c00877)

Supporting Information

Discovery of inhibitory fragments that selectively target Spire2-FMN2 interaction

Radoslaw Kitel[1,*], Ewa Surmiak[1], Jan Borggräfe[2,3], Justyna Kalinowska-Tluscik[1], Przemyslaw Golik[1], Miroslawa Czub[1], Wiktor Uzar[1,4], Bogdan Musielak[1], Mariusz Madej[5], Grzegorz M. Popowicz[2,3], Grzegorz Dubin[6], Tad A. Holak[1]

1 - Jagiellonian University, Faculty of Chemistry, Gronostajowa 2, 30-387 Kracow, Poland,

2 – Institute of Structural Biology, Molecular Targets and Therapeutics Center, Helmholtz Zentrum München, Neuherberg, 85764 München, Germany

3 – Bavarian NMR Center, School of Natural Sciences, Technical University of Munich, Garching, 85748 München, Germany

4 - Jagiellonian University, Doctoral School of Exact and Natural Sciences, prof. S. Lojasiewicza 11, 30-348 Krakow, Poland

5 - Jagiellonian University, Faculty of Biochemistry, Biophysics and Biotechnology, Gronostajowa 7, 30-387 Cracow, Poland

6 - Malopolska Centre of Biotechnology, Jagiellonian University, Gronostajowa 7A, 30-387 Krakow, Poland

***Radoslaw Kitel. Email: radoslaw.kitel@uj.edu.pl**

Supplementary Figures, Tables and Methods

Table S1: Fragment-based screening data ……………………………………….……..………S2
Figure S1: Establishment of fluorescence polarization assay …………………….……………….S3
Figure S2: Evaluation of binding of F408 to KIND2 Y106A domain ……………………….…S4
Figure S3: The spectrum of KIND2 domain with assigned backbone resonances …………..S5
Figure S4: Titrations data of compound **13** …………………………………..……………S6
Figure S5: Comparison of KIND1 and KIND2 domain sequences …………………………..S7
^{1}H and ^{13}C spectra of tested fragments ……………………………………………………S8-S26
LC-MS chromatograms of tested fragments …………………………………………..….S27-S43

Table S1: Fragment-based Screening Data

Category	Parameter	Description
**Assay**	Type of Assay	Thermal Shift Assay (DSF)
	Target	KIND1 and KIND2
	Primary Measurement	Melting curves of the target proteins in the presence of fragments
	Key Reagents	SYPRO-ORANGE
	Assay Protocol	Please refer to the materials and methods section.
**Library**	Library size	755 compounds
	Library composition	In-house library of Ro3-compliant fragments. Compounds stored at -20 °C as 50 mM stock solutions in 100% DMSO-*d6*.
	Source	In-house, Sigma Aldrich,
**Screen**	Format	96-well plates
	Concentration(s) of fragments	1 mM fragment/2% DMSO
	Plate controls	Positive control: FSI peptide (2 replicates per plate) negative control: 2% DMSO (2 replicates per plate)
	Reagent/compound dispensing system	Multichannel pipette
	Detection instrument and software	CFX96TM (BioRad)
	Normalization	ΔTm (°C)
**Post-screening analysis**	Hit criteria	Positive and negative thermal shift between +0.8 and -2.0 °C
	Hit rate	2.5% (KIND1), 6,2% (KIND2).
	Additional assay(s)	MST, FP, ^{1}H-^{15}N-HSQC NMR
	Confirmation of hit purity and structure	Resynthesis of fragment F408, ^{1}H and ^{13}C NMR, LC-MS, HRMS

Establishment of fluorescence polarization assay

The establishment of the fluorescence polarization assay was done according to protocols described previously.

In the first step, the affinity of the fluorescently labelled reporter peptide (FSI-peptide, FITC-Ahx-GKSLYKIKPRHDSGIKAKISMKT-OH) was obtained. This was done by taking measurements with constant concentration of the reporter peptide (1 µM) and varying concentrations of KIND1 or KIND2 domains. This yielded dose-response curves that allow us to calculate the Kd of FSI-peptide. The affinity was determined to be 0.39±0.06 µM and 0.83±0.2 µM, for the KIND1 and KIND2 domains respectively.

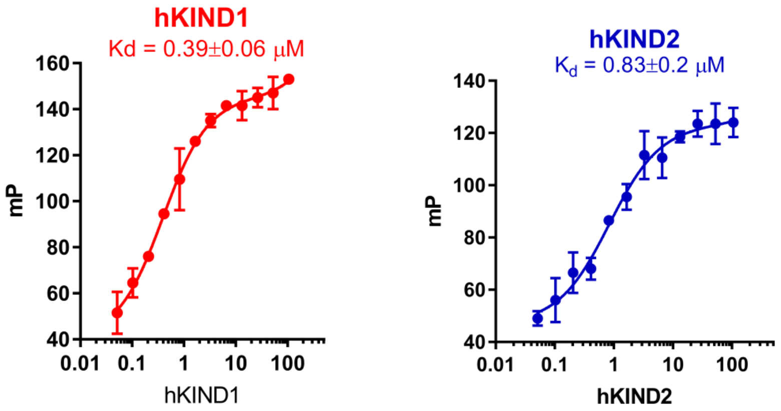

Figure S1. Calculation of K_d values of FSI reporter peptide to KIND1 (red curve) and KIND2 (blue curve).

In the next step, the working concentration of the proteins was calculated. Typically, the concentration should be in a dynamic range (0.5-0.8). For calculations, the following equation was used:

$$f_0 = (P - P_{min})/(P_{max} - P_{min})$$

Where:

P – fluorescence polarization in the dynamic range (0.5-0.8),

P_{min} – average minimal fluorescence polarization,

P_{max} – average maximal fluorescence polarization,

Assuming f_0 = 0.75, P values were calculated to be 128.25 and 102.50 for KIND1 and KIND2, respectively.

Taking into account K_d values for the reporter peptide, the working concentration of the proteins were calculated as follows:

$$c = K_d(P - P_{min})/(P_{max} - P)$$

c_{KIND1} = 1.17 µM c_{KIND2} = 2.49 µM

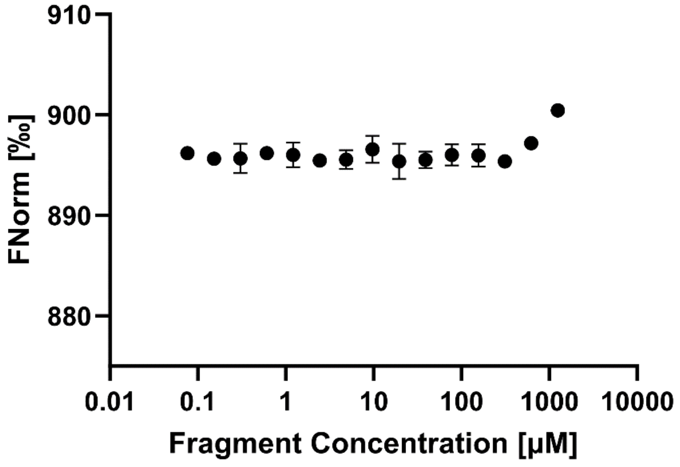

Figure S2: Evaluation of binding of F408 to KIND2 Y106A domain using microscale thermophoresis – no binding detected up to the concentration of 2500 μM

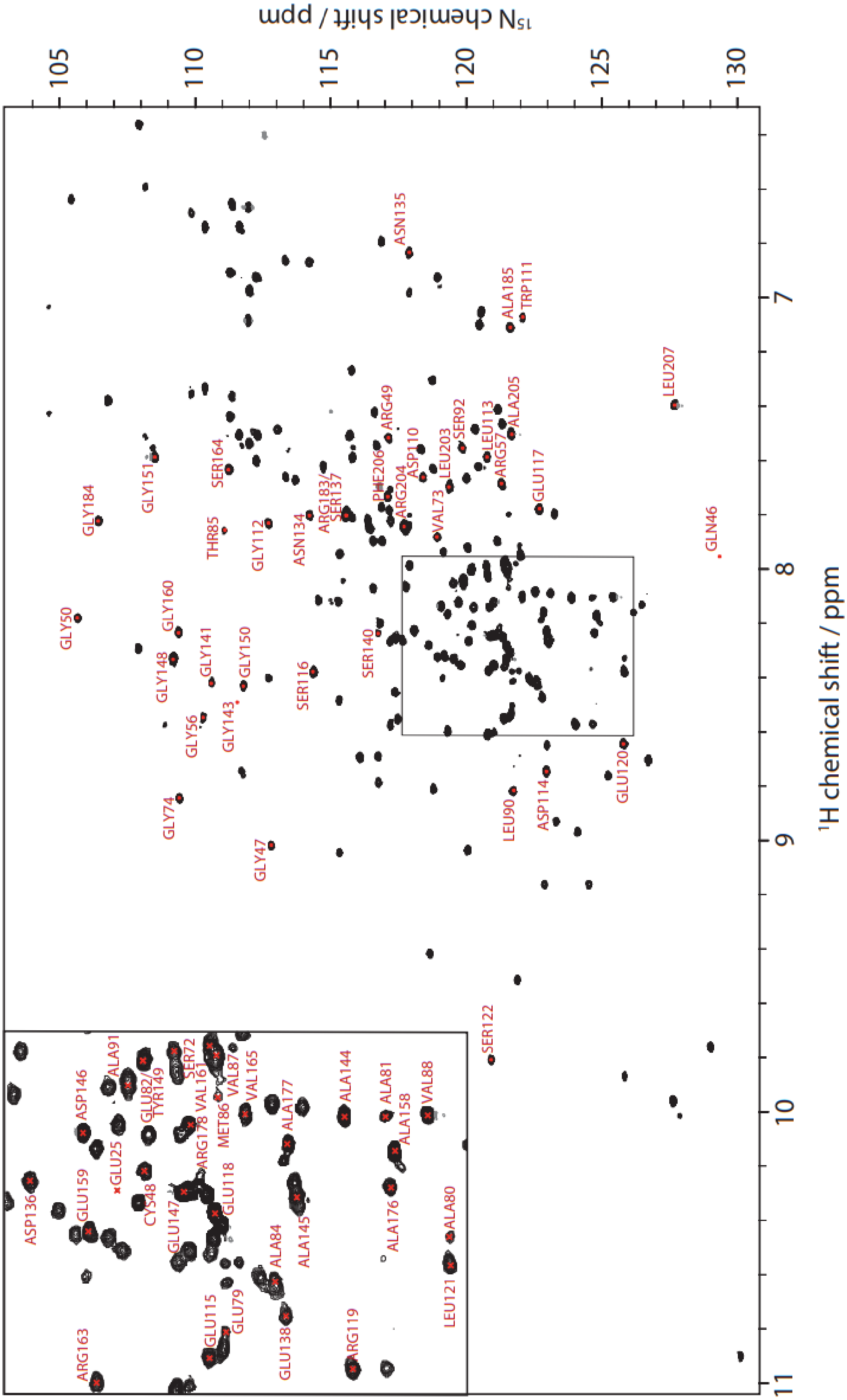

Figure S3: The spectrum of the KIND2 domain with assigned backbone resonances

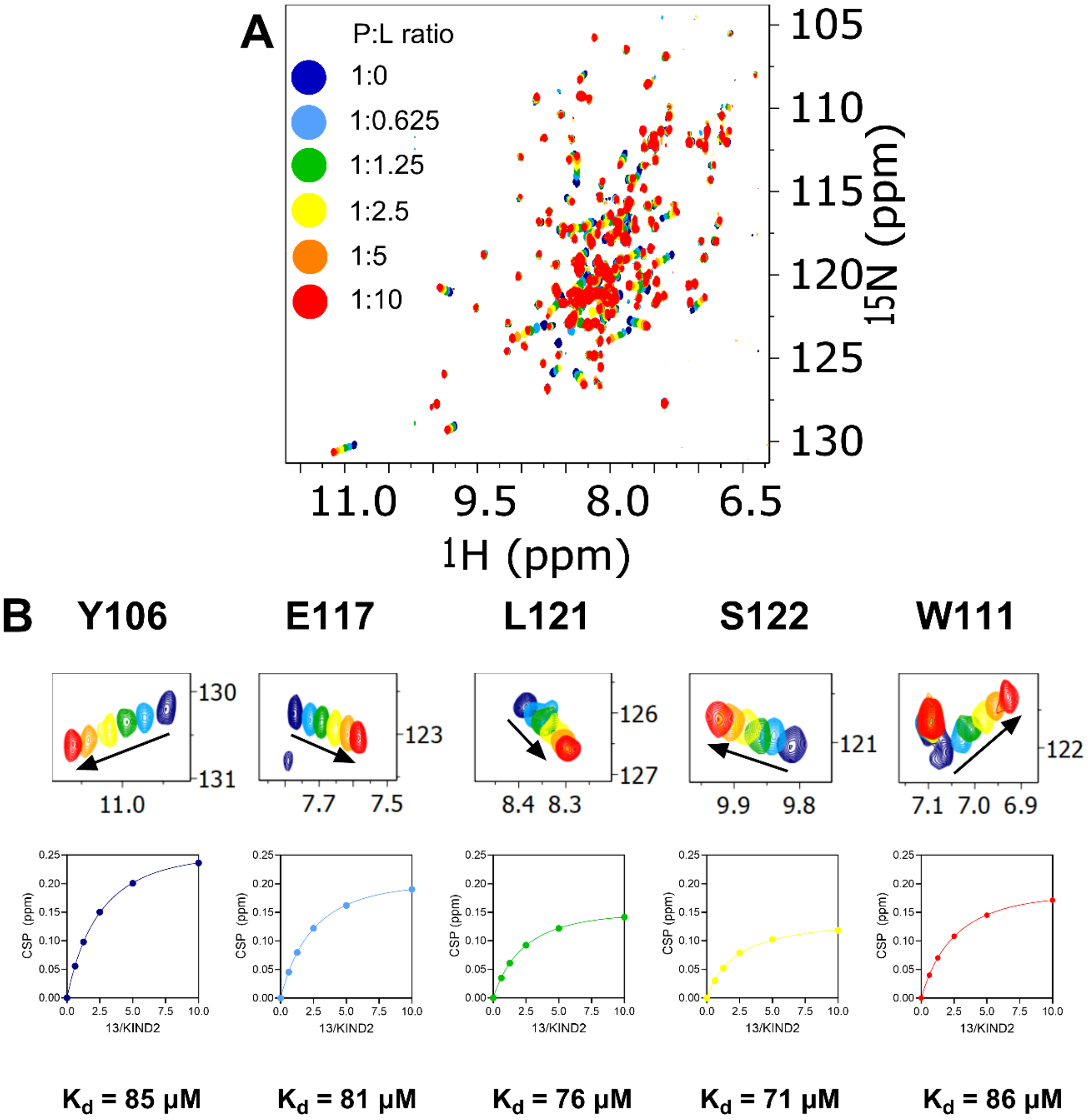

Figure S4: (A) $^{1}H,^{15}N$ HSQC overlays of the KIND2 domain titrated with increasing concentrations of **13.** (B) Calculation of K_d for compound 13; Upper panel: Insets showing the five most perturbed peaks with increasing concentration of fragment **13**, protein: ligand ratios (P:L) as follows: 1:0 (navy), 1:0.625 (blue), 1:1.25 (green), 1:2.5 (yellow), 1:5 (orange) and 1:10 (red); lower panel: Plots of normalized chemical shift perturbation as a function of protein: ligand ratio was used to estimate the average K_d of **13** to KIND2 (79.8±6 μM).

```
KIND1-HS    40    LSLEEILRLYNQPINEEQAWAVCYQCCGSLRAAARRRQPRHRVRSAAQIRVWRDGAVTLA    99
KIND2-HS    22    LSLEEVLKAYEQPLNEEQAWAVCFQGCRGLRGS-----PGRRLRDTGDLLLRGDGSV--G    74
KIND1-HS    100   PAADDAGEPPPVAGKLGYSQCMETEVIESLGIIIYKALDYGLKENEERELSPPLEQLIDH   159
KIND2-HS    75    AREPEAAEPATMVVPLASS---EAQTVQSLGFAIYRALDWGLDESEERELSPQLERLIDL   131
KIND1-HS    160   MANTVEADGSNDEGYEAAEEGLGDEDEKRKIS----AIRSYRDVMKLCAAHLPTESDAPN   215
KIND2-HS    132   MAN----NDSEDSGCGAADEGYGGPEEEEEAEGVPRSVRTFAQAMRLCAARLTDPRGAQA   187
KIND1-HS    216   HYQAVCRALFAETMEL                                               231
KIND2-HS    188   HYQAVCRALFVETLEL                                               203
```

Figure S5. Comparison of aminoacid sequences of human KIND1 (40 – 231, Uniprot Q08AE8) and KIND2 (20 - 203, Uniprot Q8WWL2) domains.

N^1-(7-chloroquinolin-4-yl)ethane-1,2-diamine (F408)

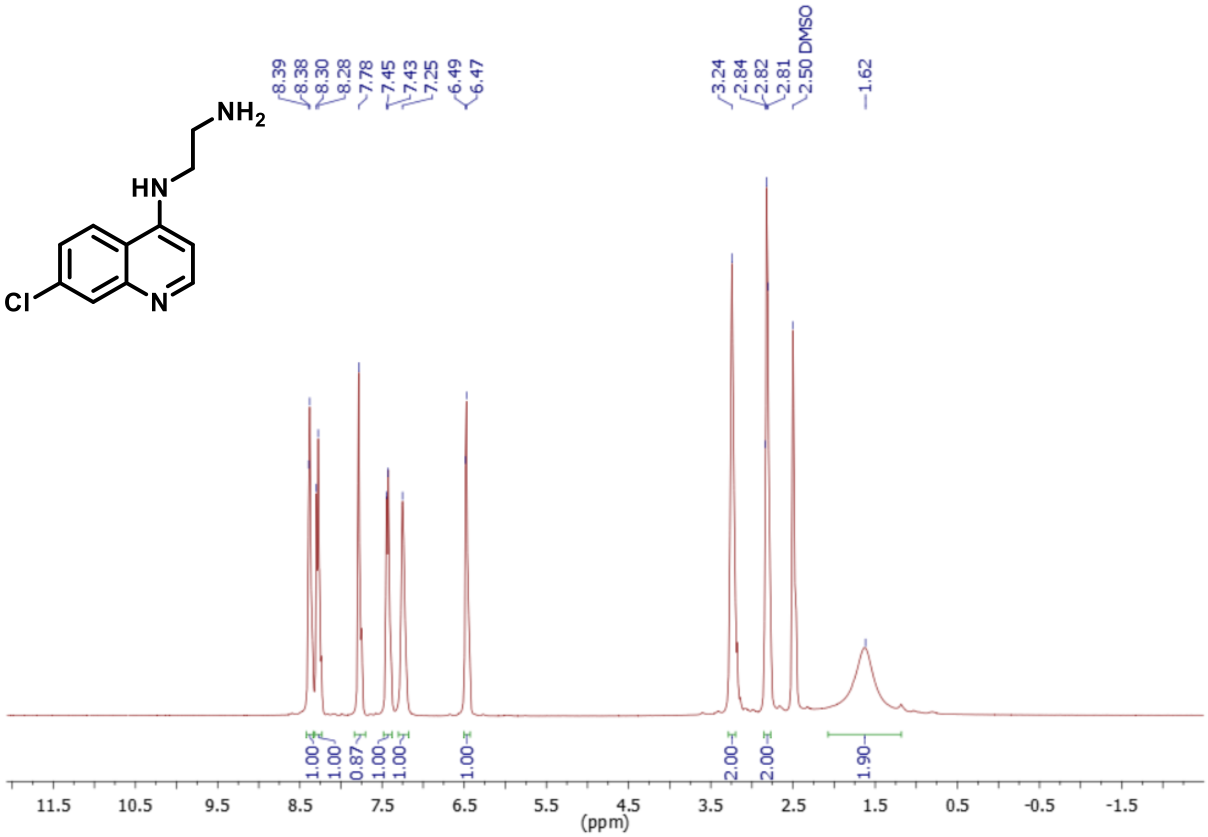

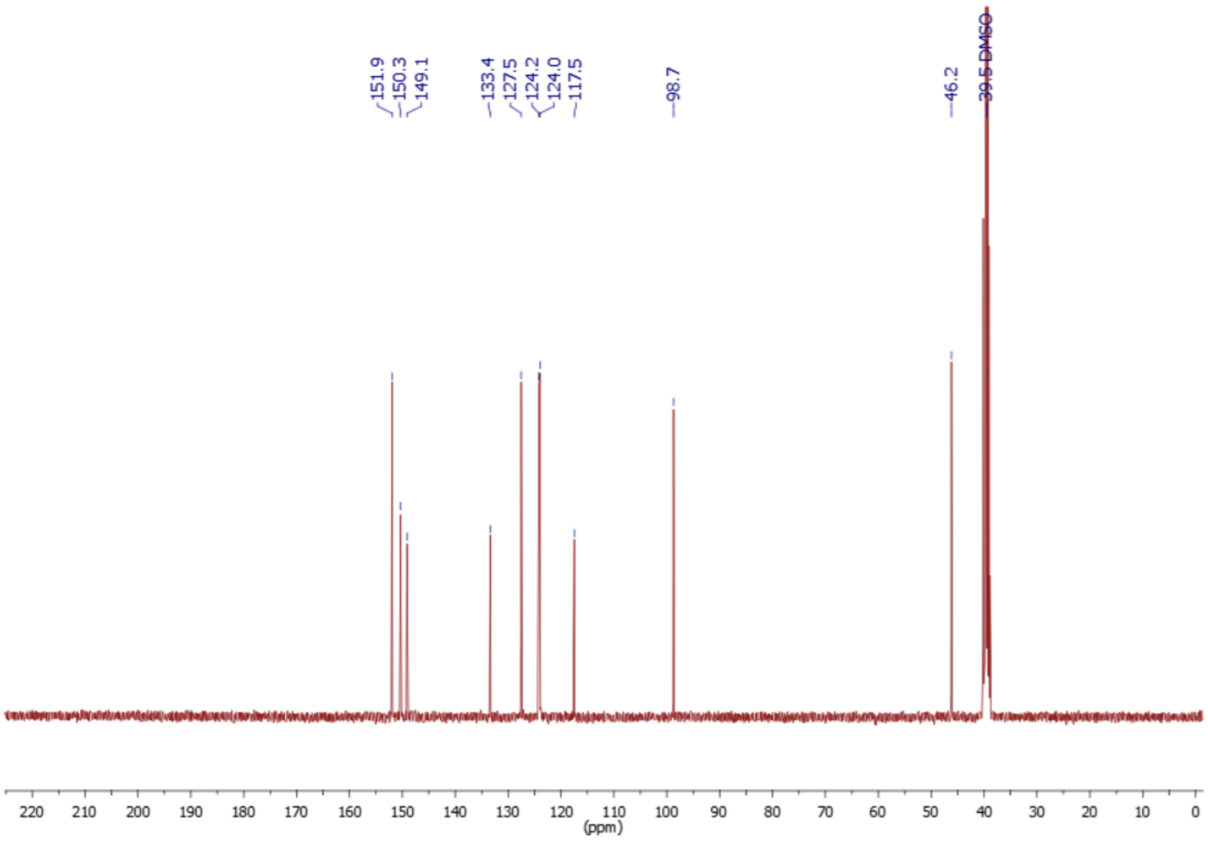

2-((7-chloroquinolin-4-yl)amino)ethan-1-ol (1)

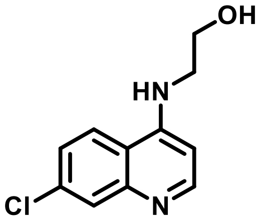

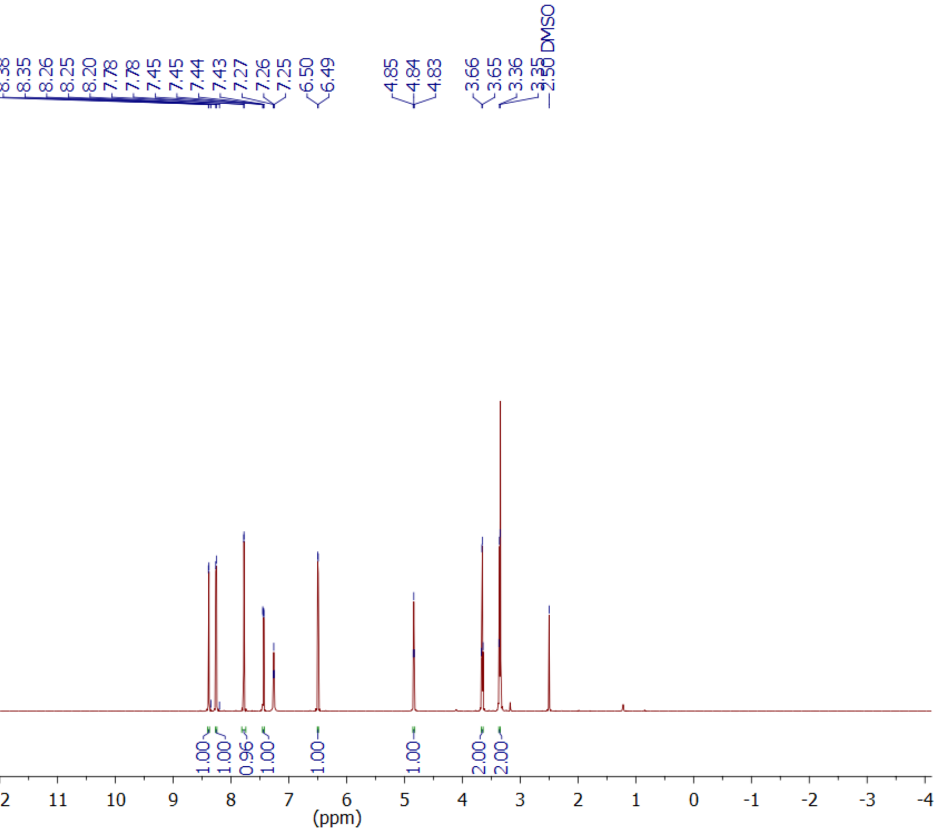

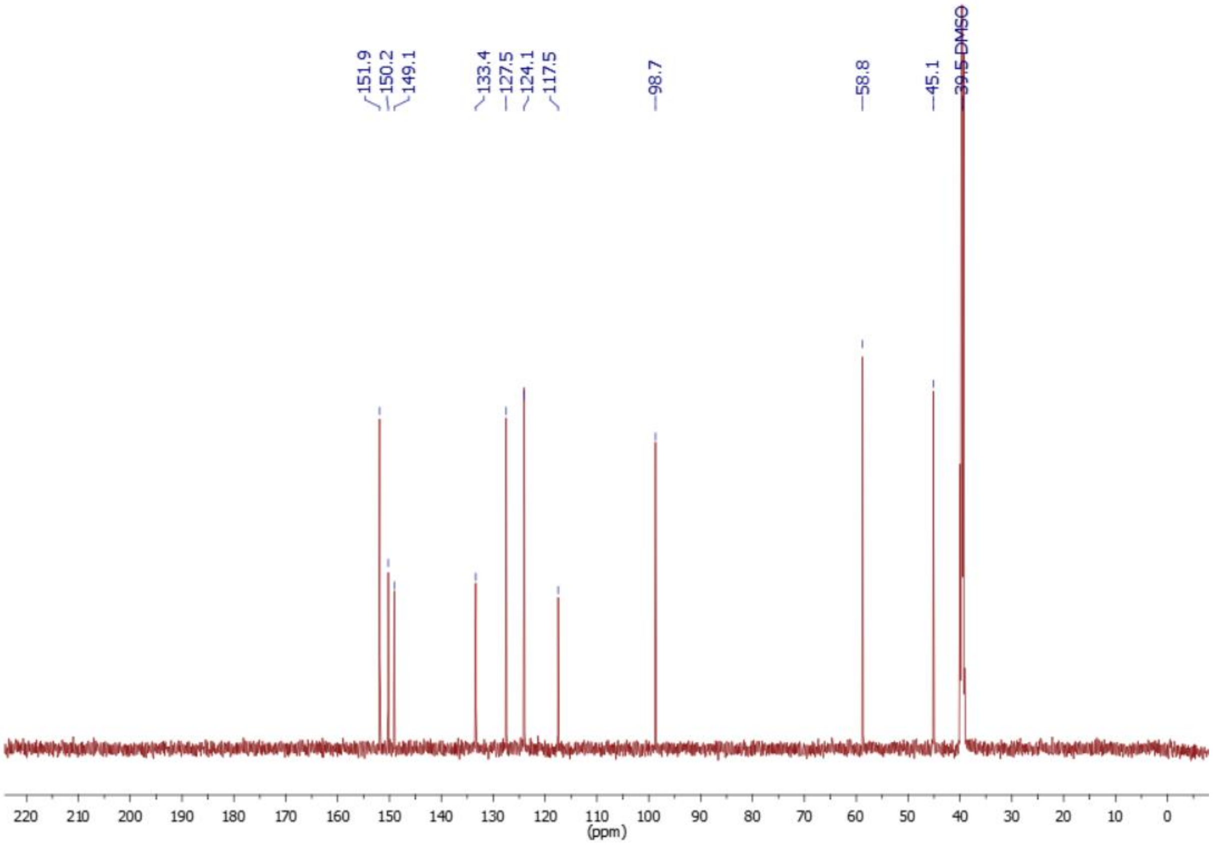

N[1]-(7-chloroquinolin-4-yl)-*N*[2],*N*[2]-dimethylethane-1,2-diamine (2)

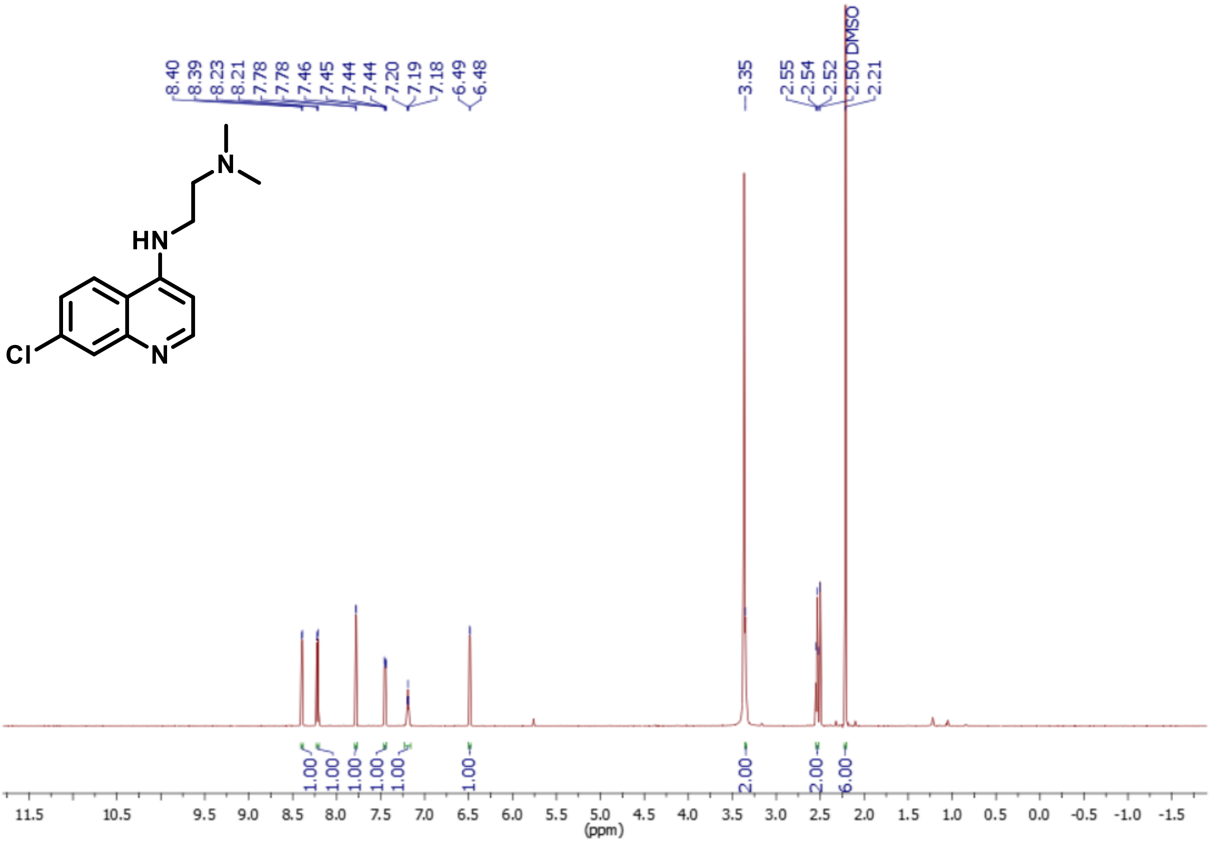

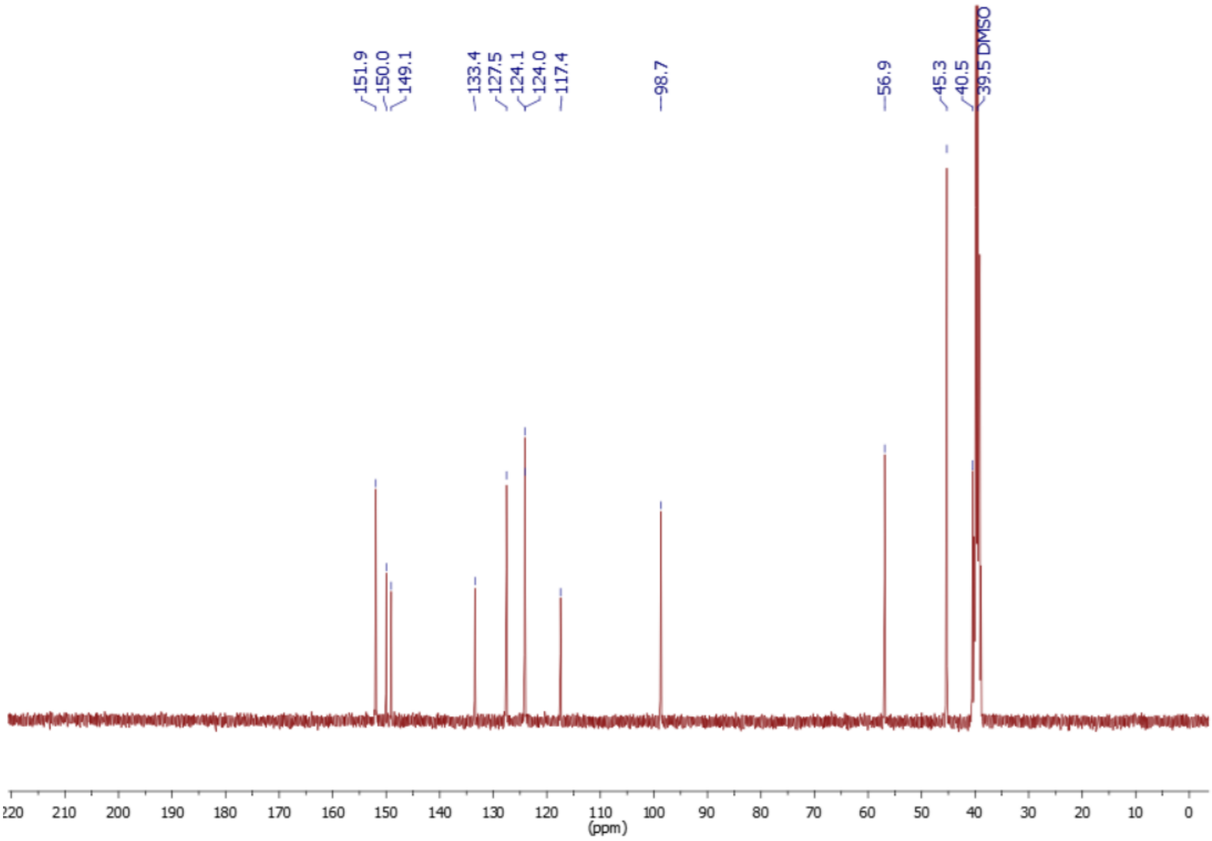

(*S*)-1-(7-chloroquinolin-4-yl)pyrrolidin-3-amine (3)

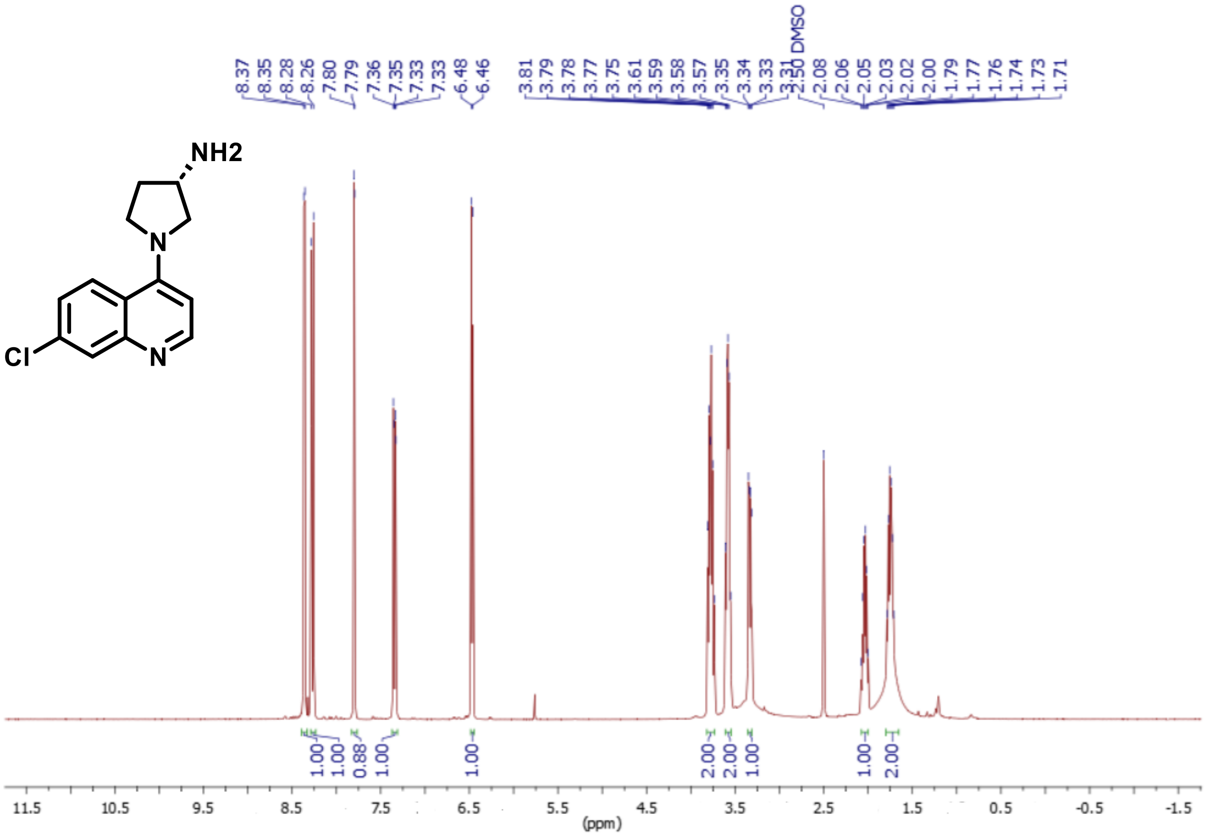

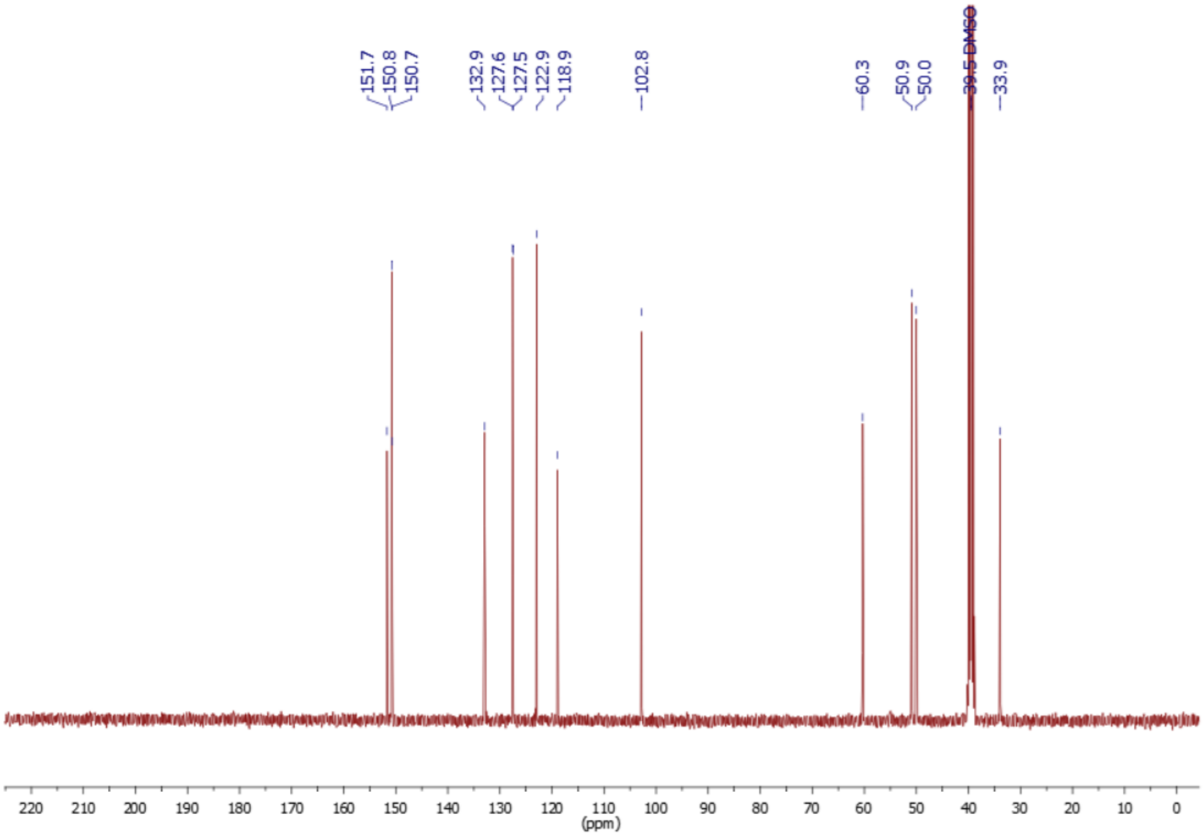

(*R*)-1-(7-chloroquinolin-4-yl)pyrrolidin-3-amine (4)

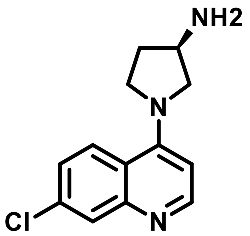

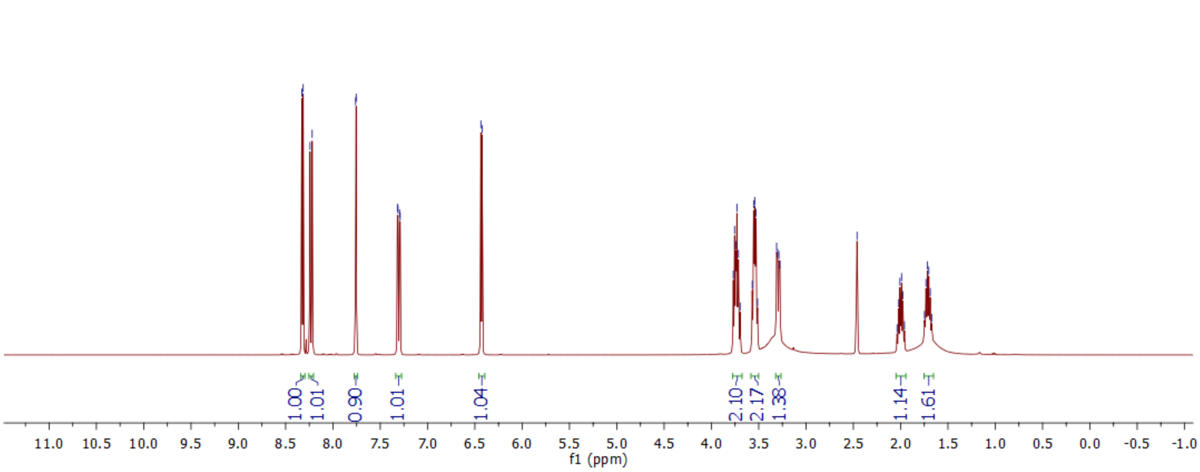

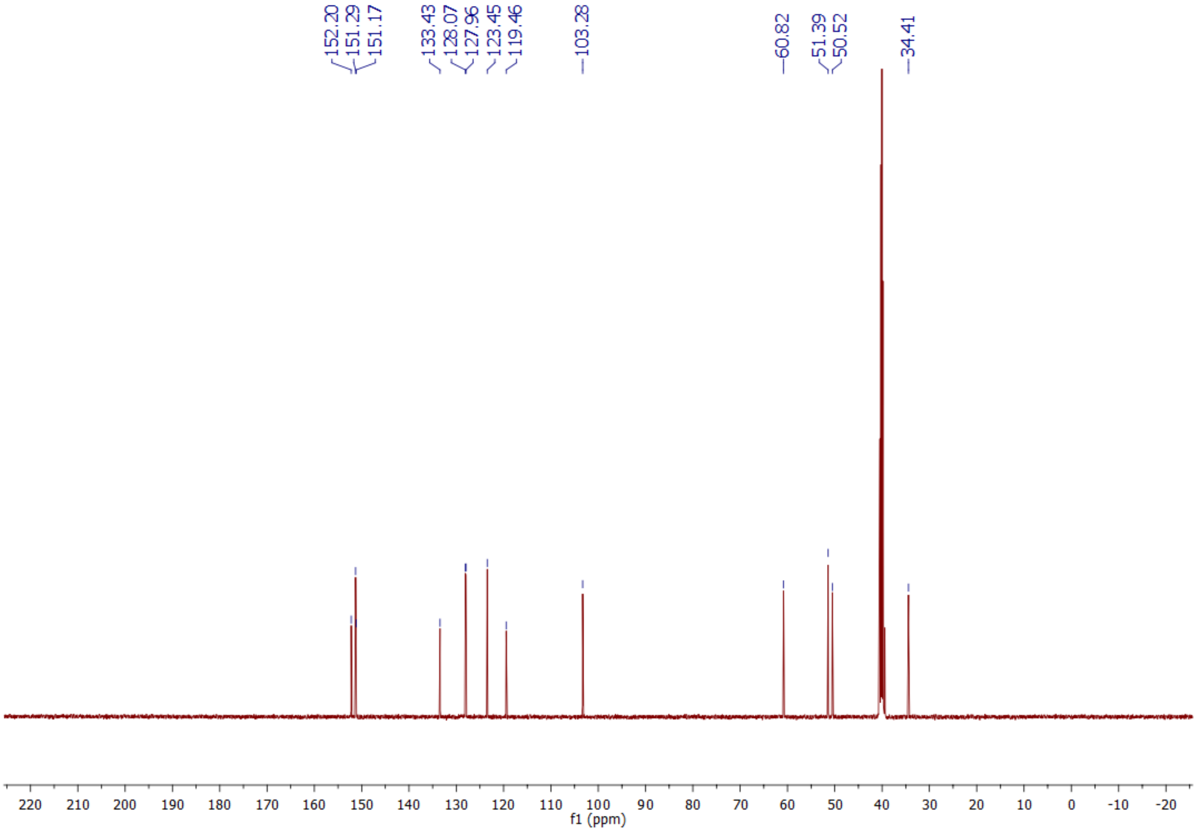

(3*S*)-1-(7-chloroquinolin-4-yl)piperidin-3-amine (5)

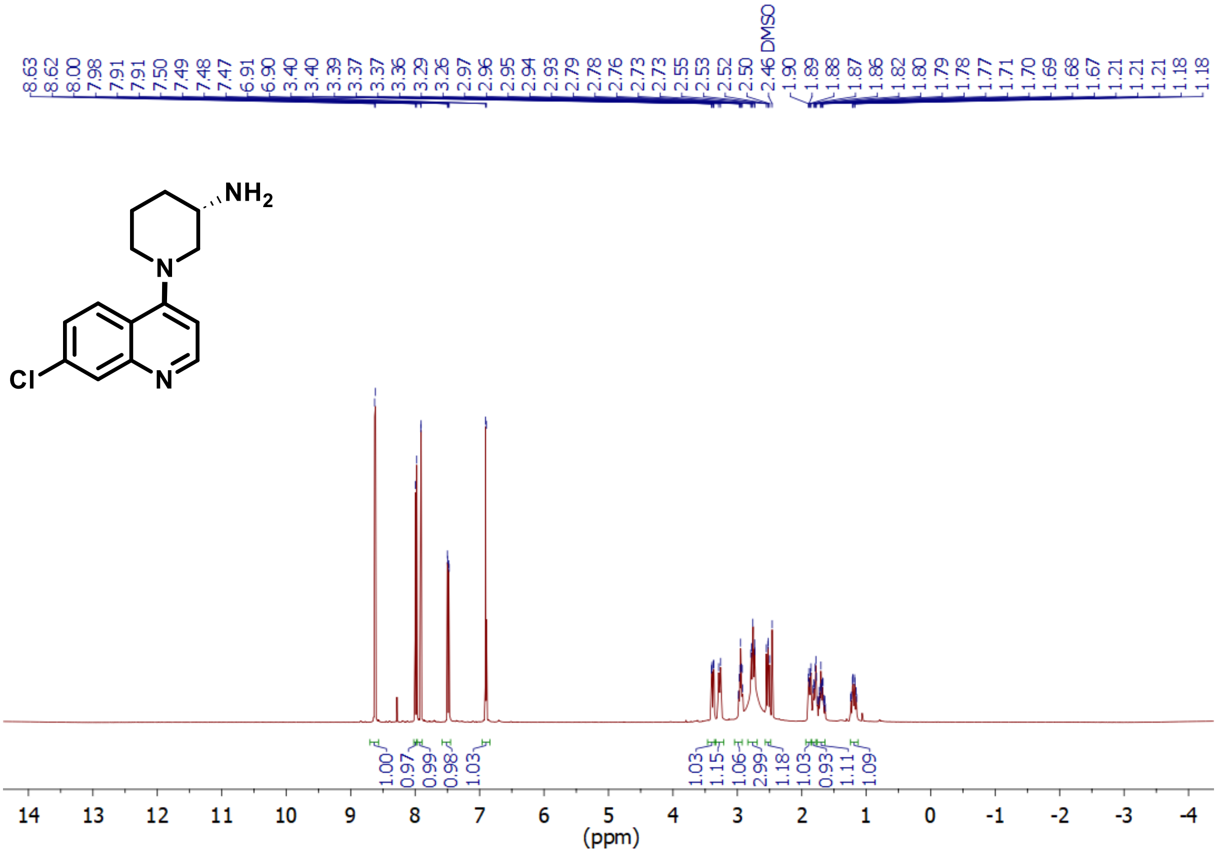

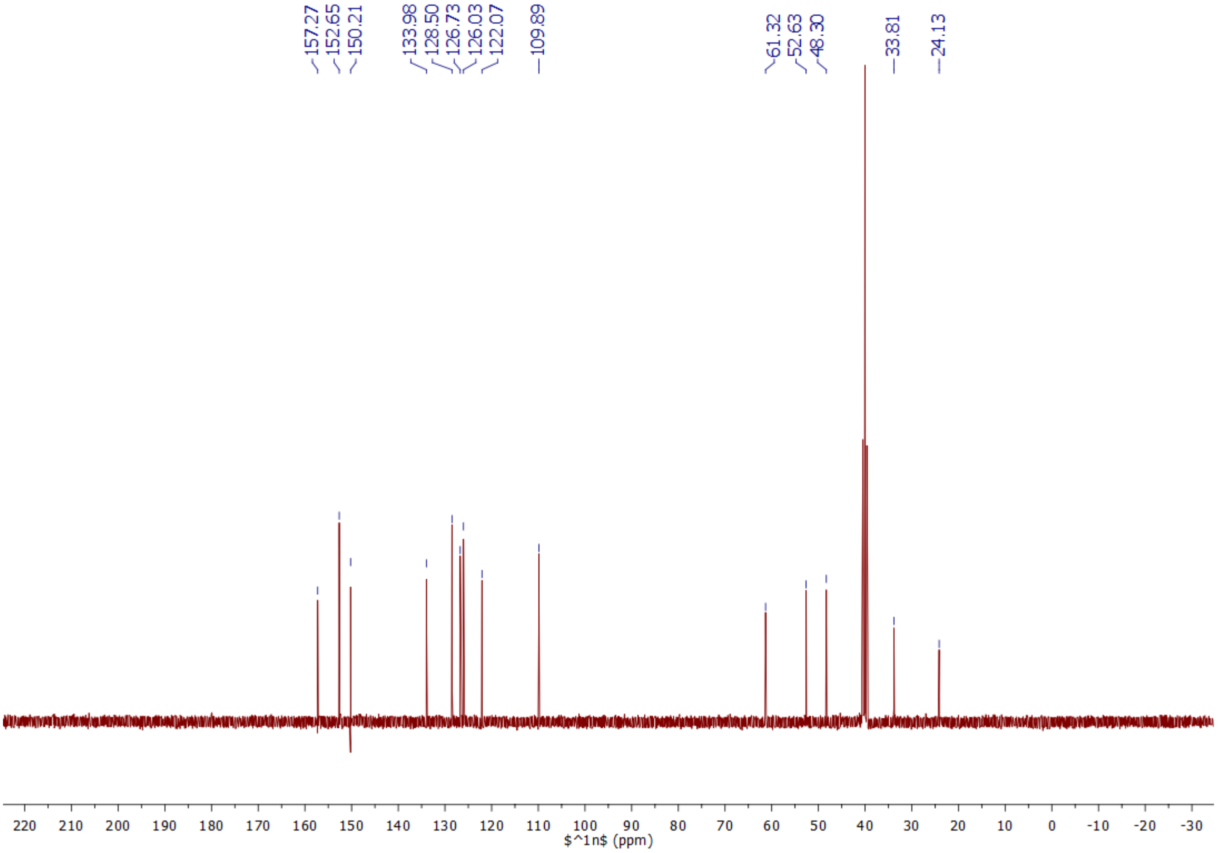

(3*R*)-1-(7-chloroquinolin-4-yl)piperidin-3-amine (6)

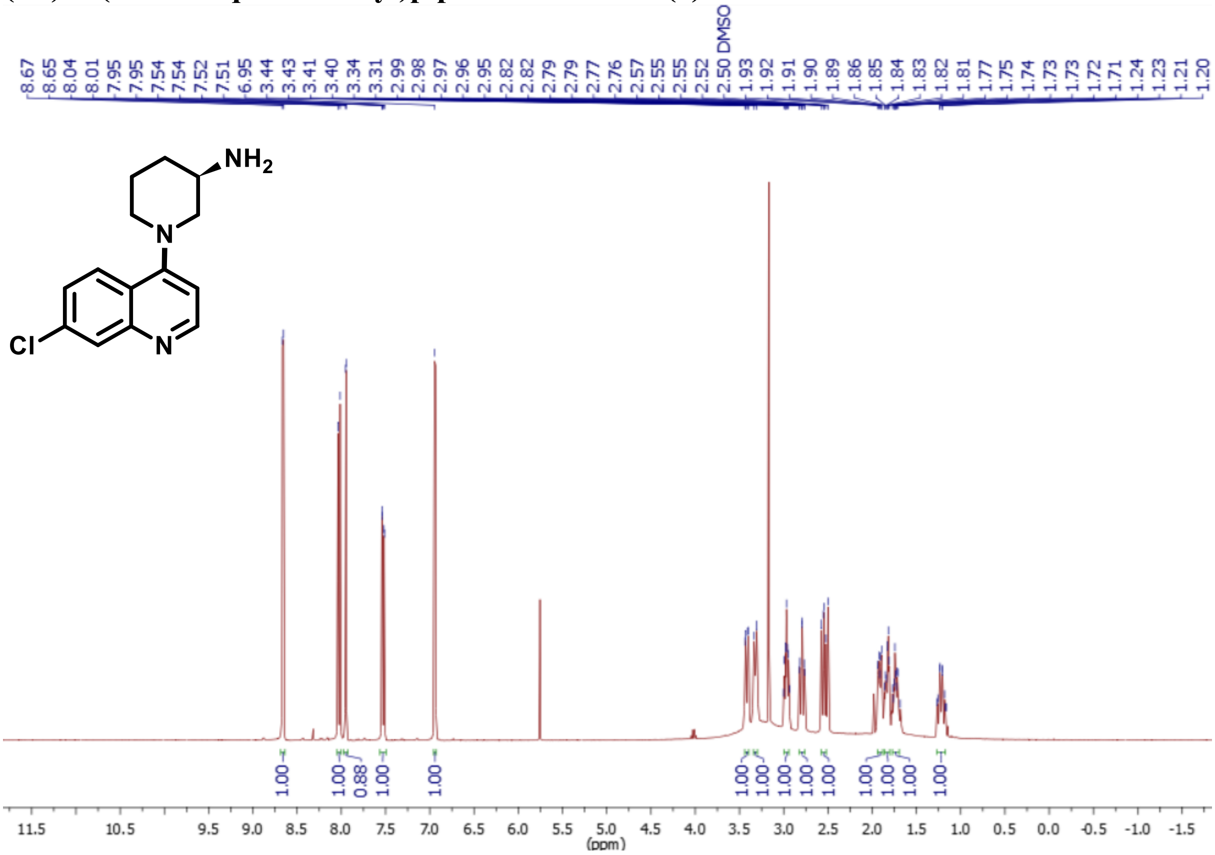

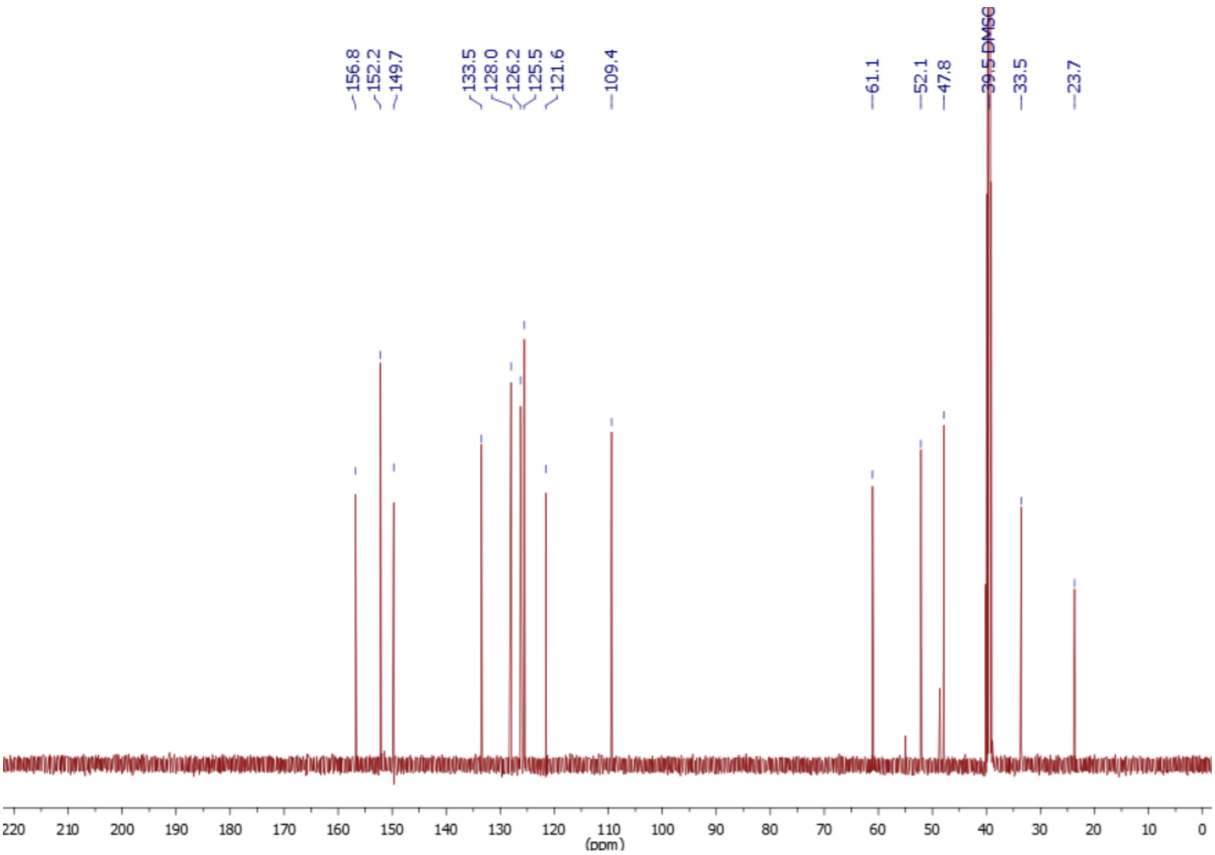

(*S*)-7-chloro-N-(piperidin-3-yl)quinolin-4-amine (7)

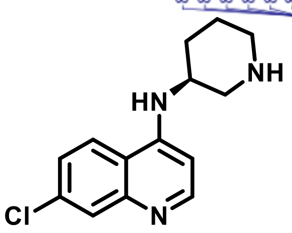

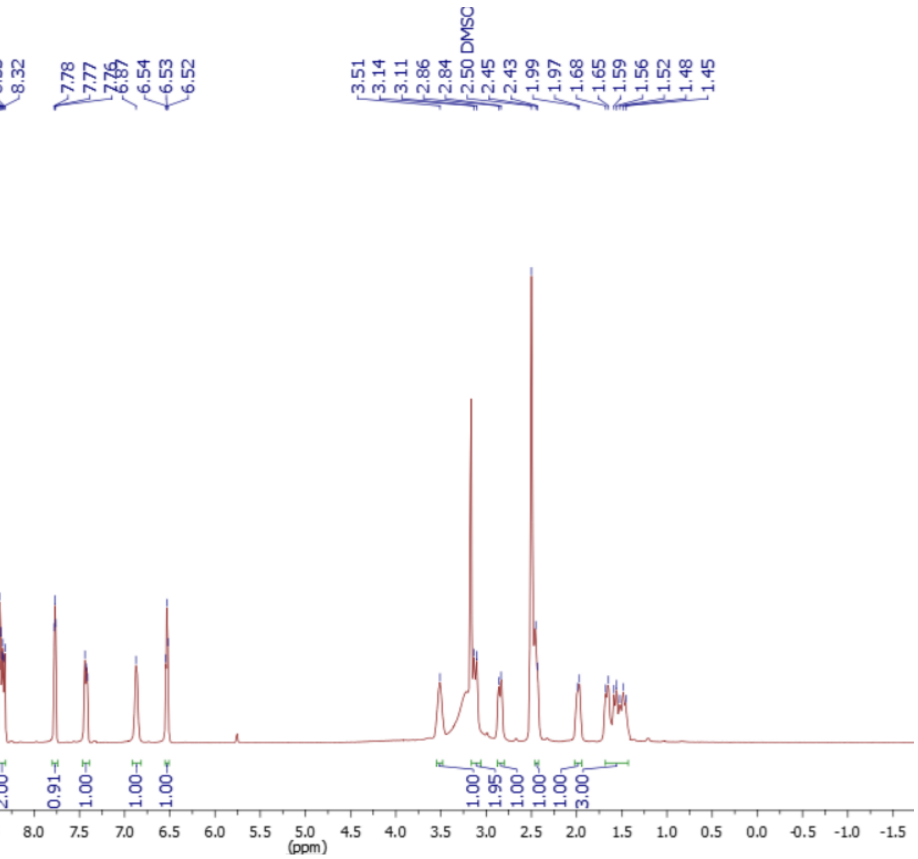

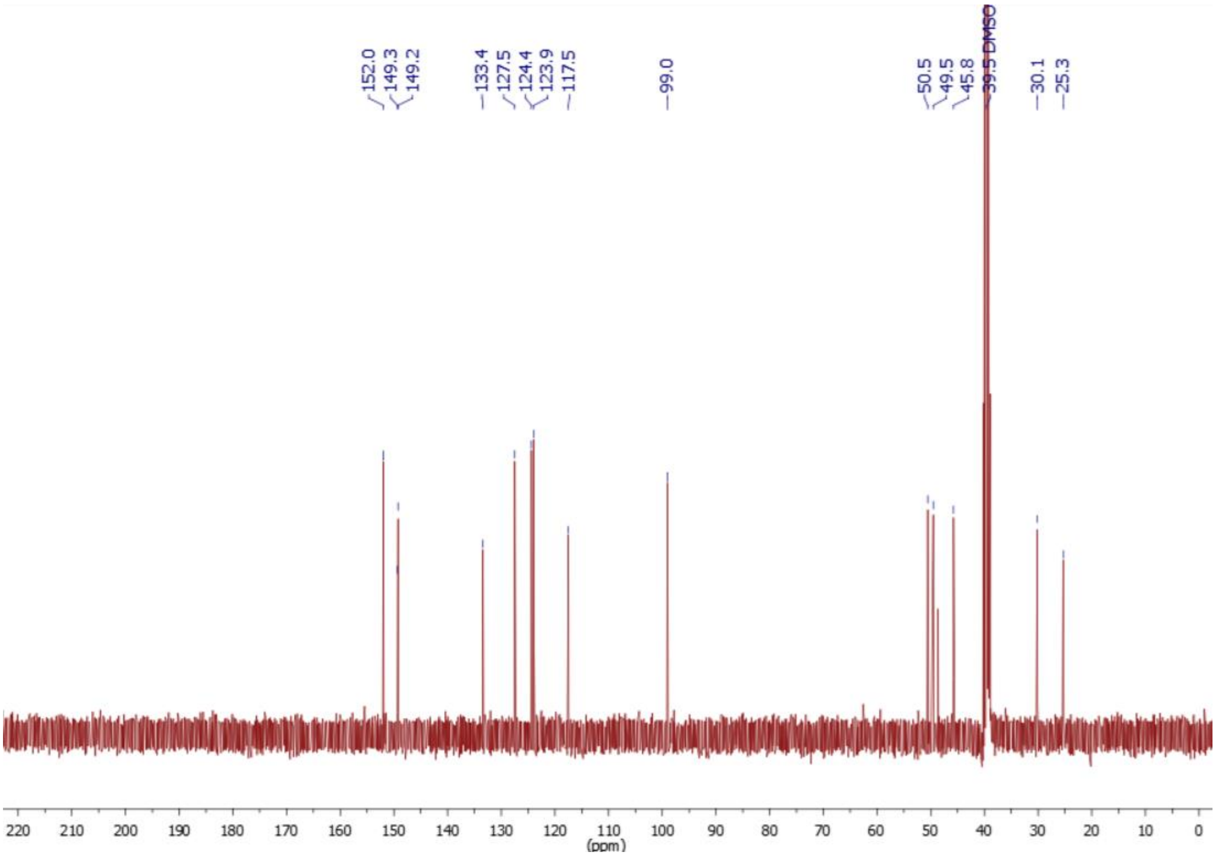

(*R*)-7-chloro-N-(piperidin-3-yl)quinolin-4-amine (8)

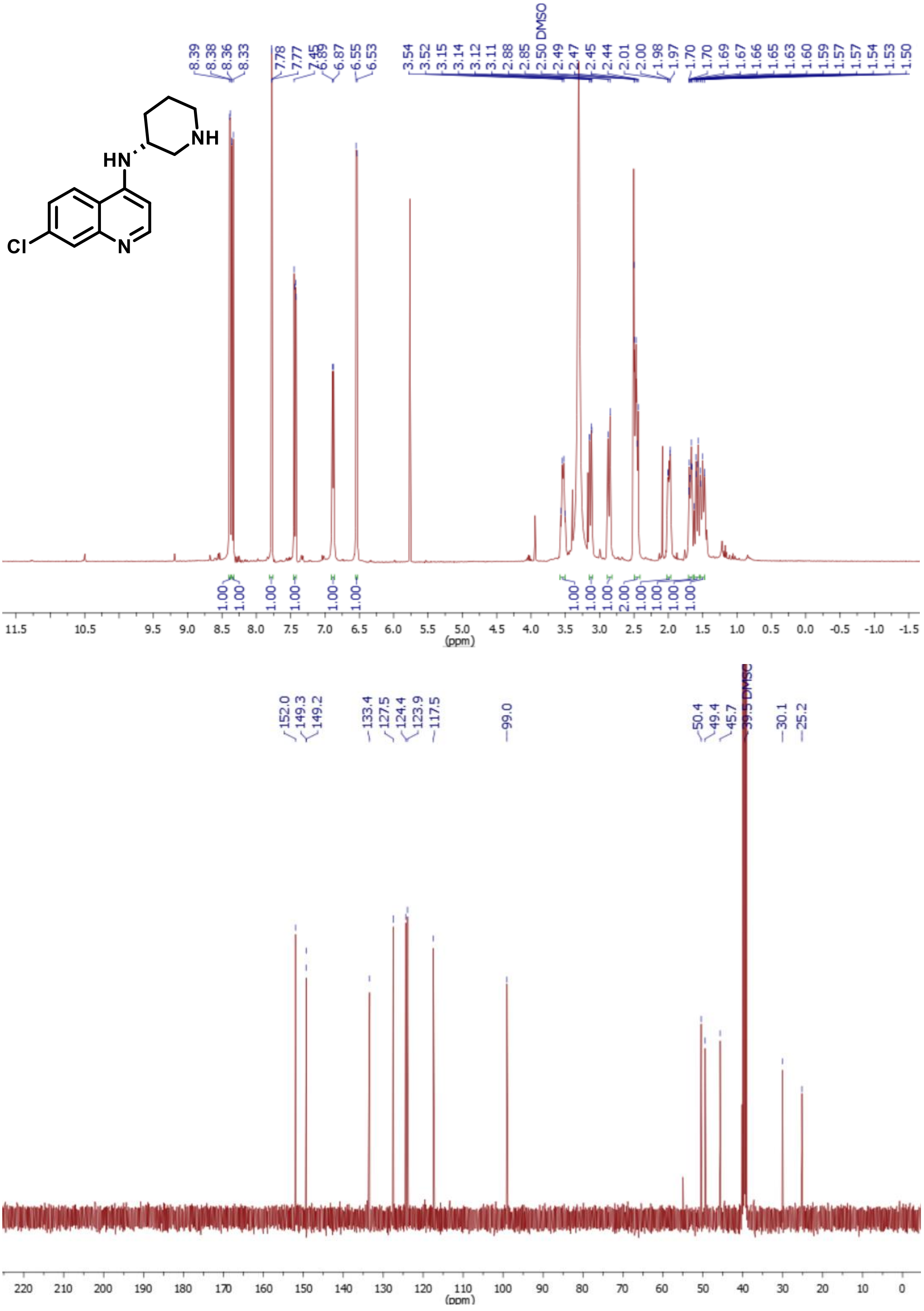

N^1-(7-(trifluoromethyl)quinolin-4-yl)ethane-1,2-diamine (9)

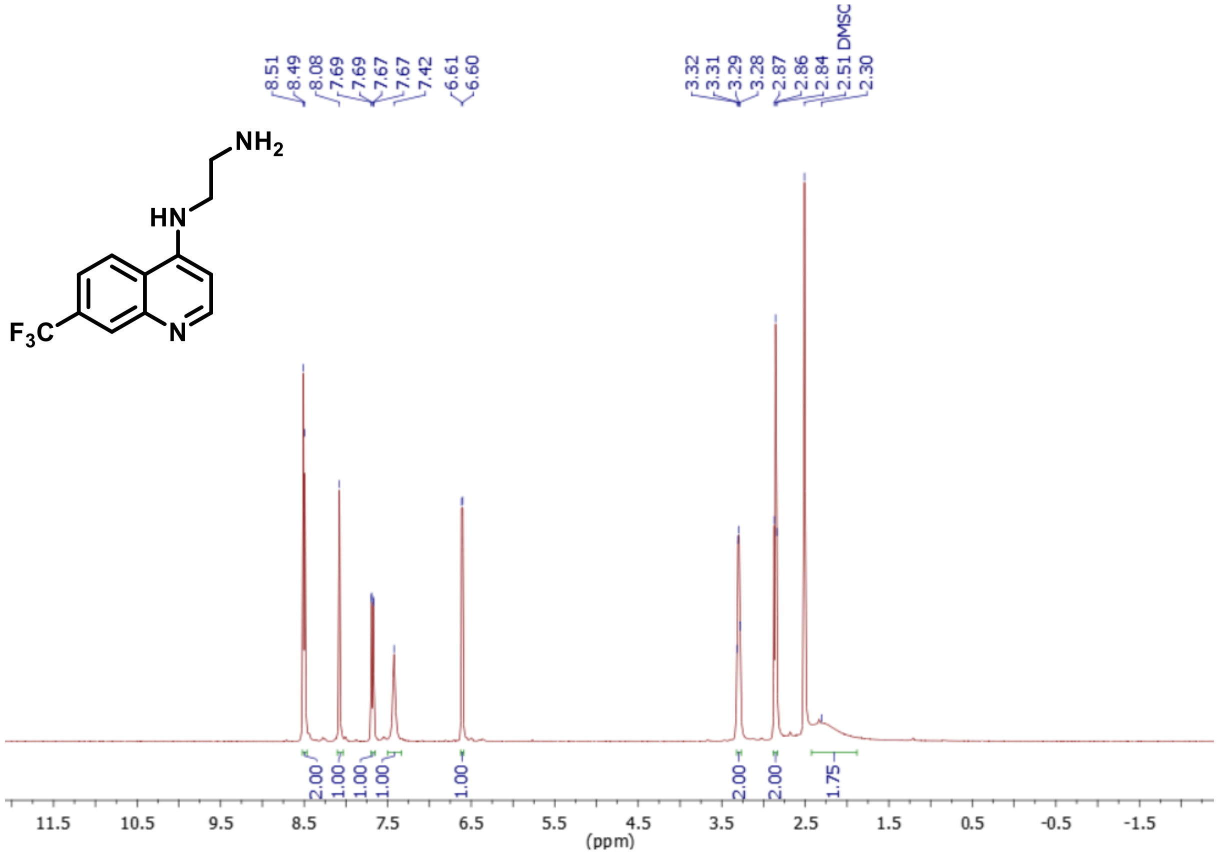

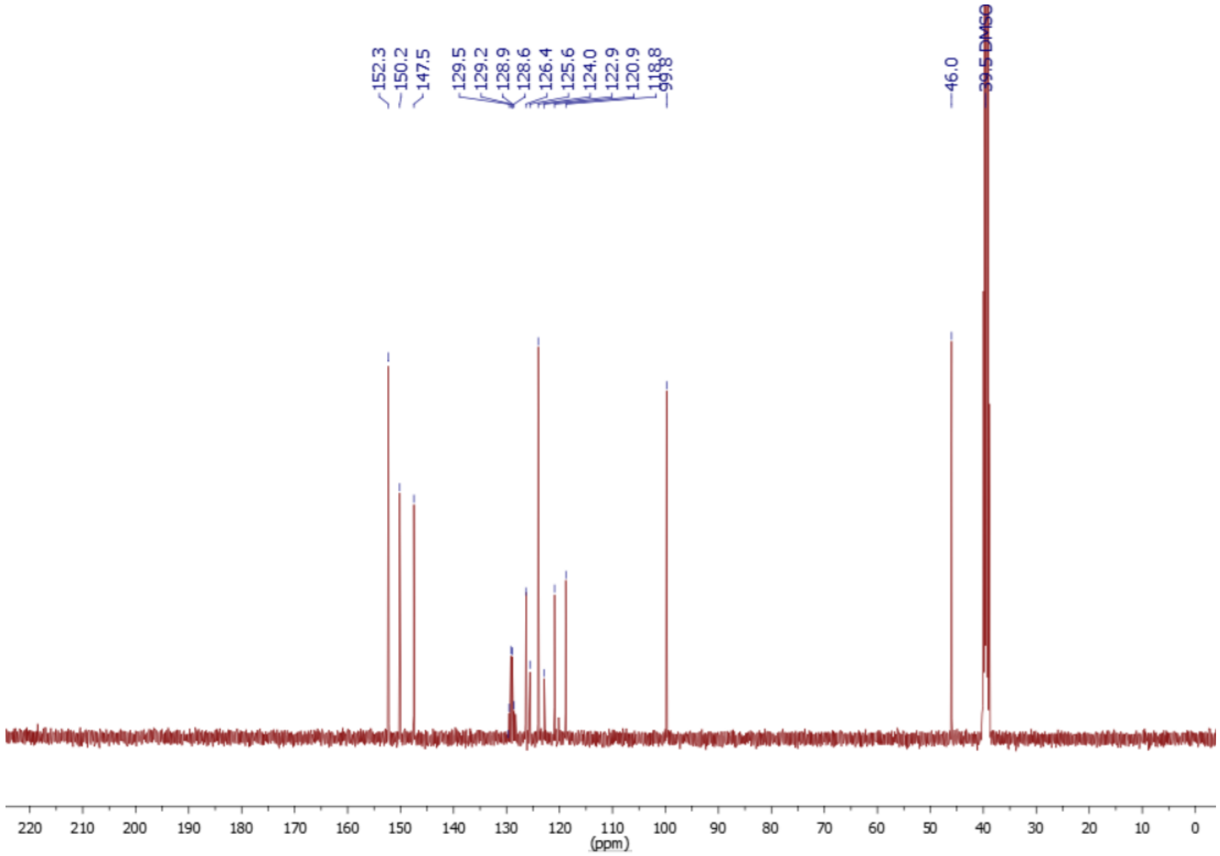

N^1-(7-methylquinolin-4-yl)ethane-1,2-diamine (10)

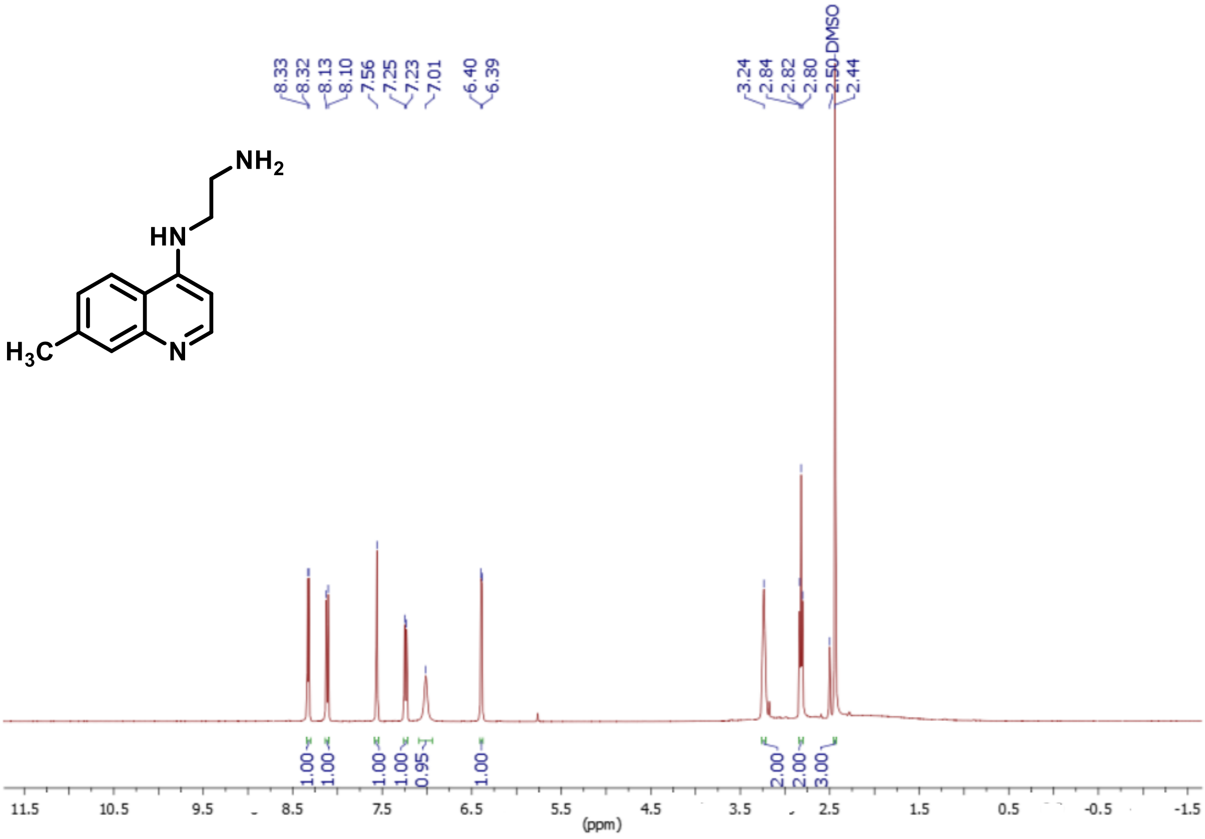

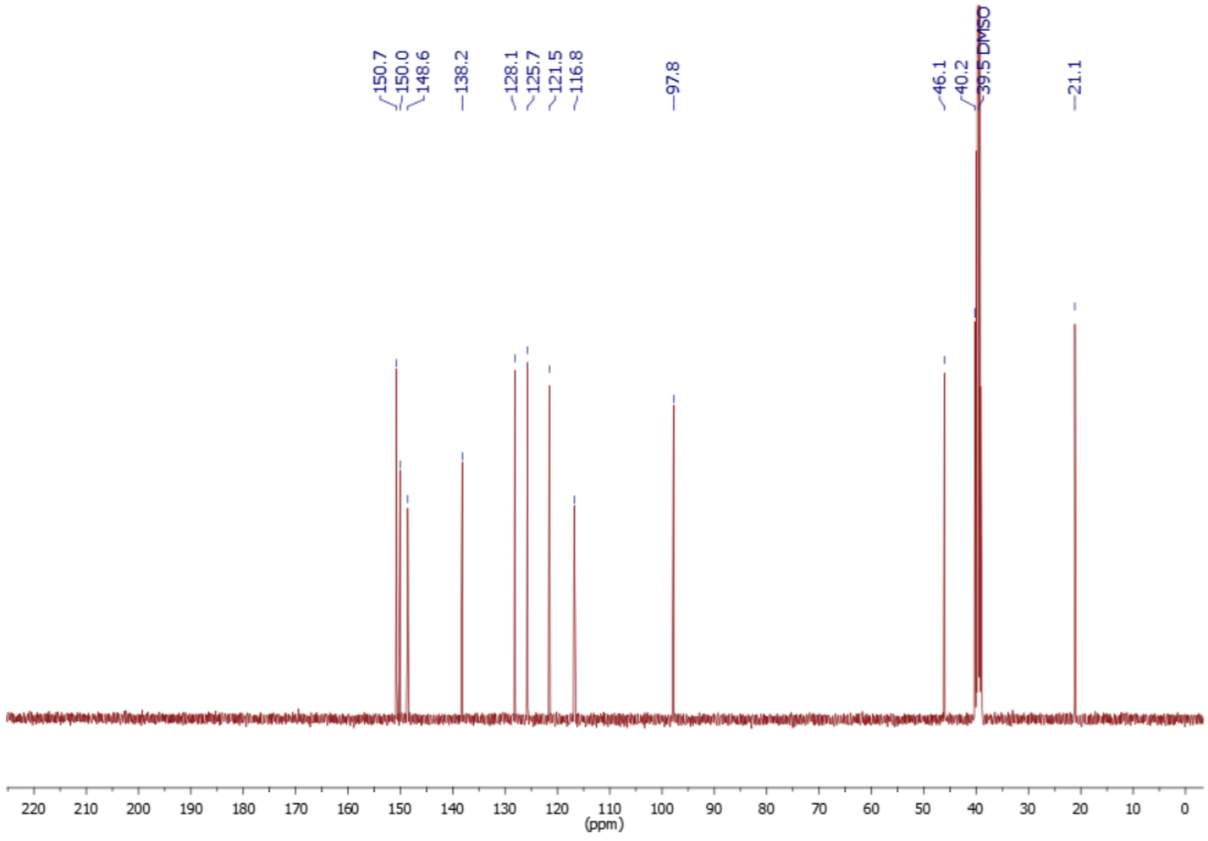

N^1-(7-bromoquinolin-4-yl)ethane-1,2-diamine (11)

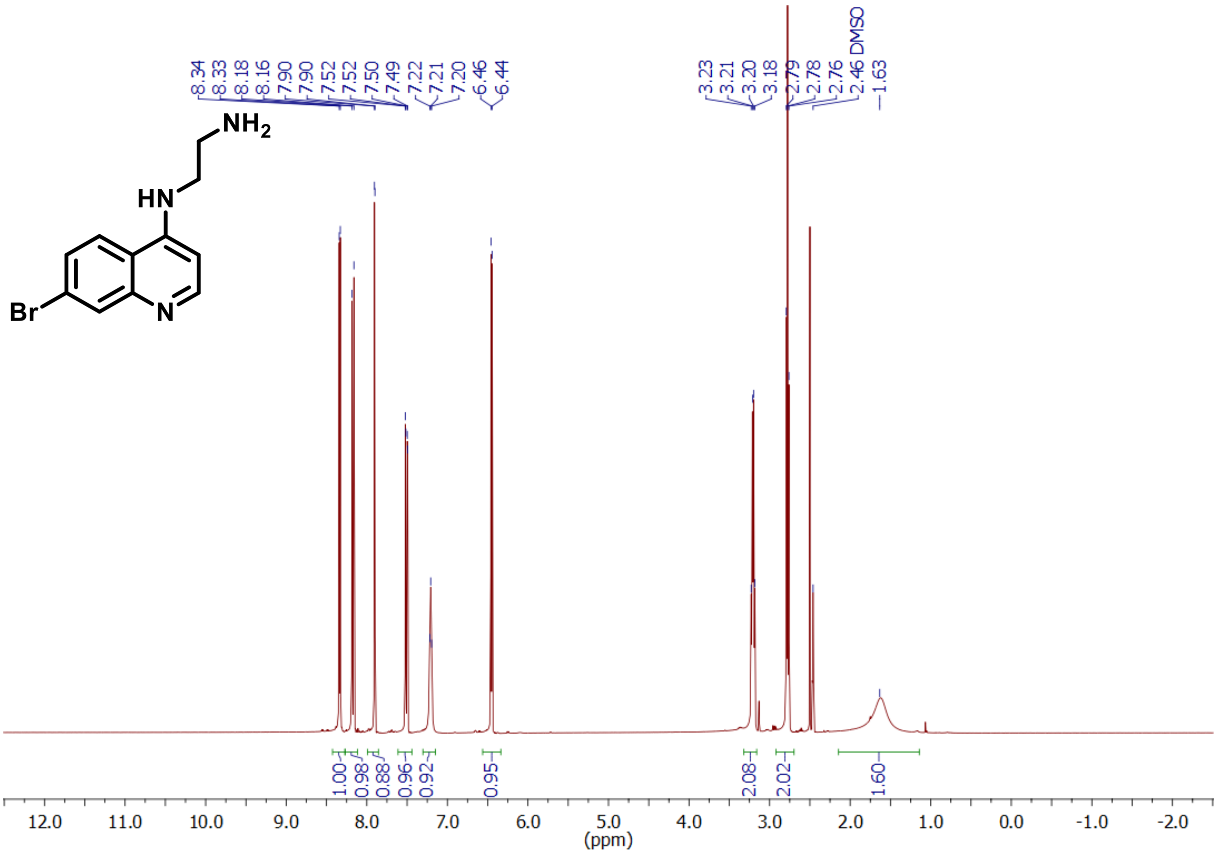

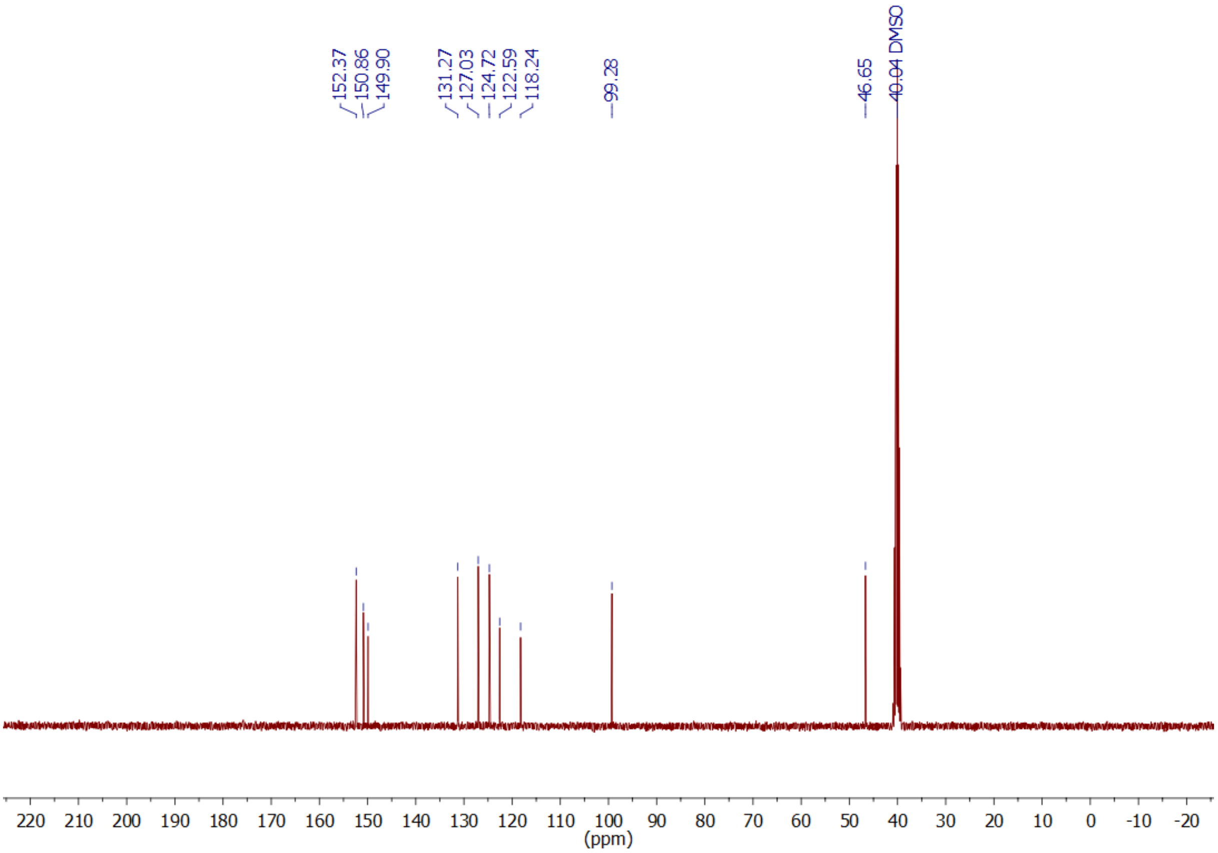

N^1-(quinolin-4-yl)ethane-1,2-diamine (12)

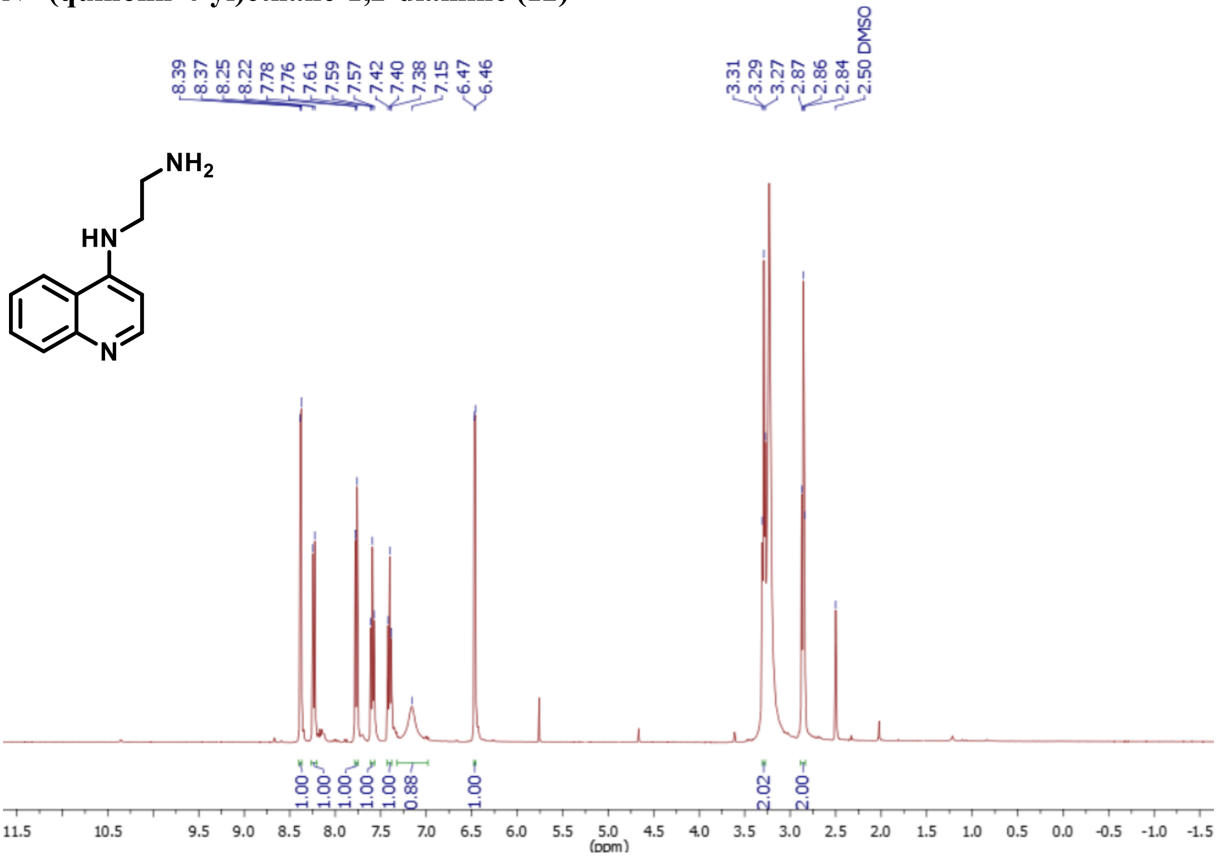

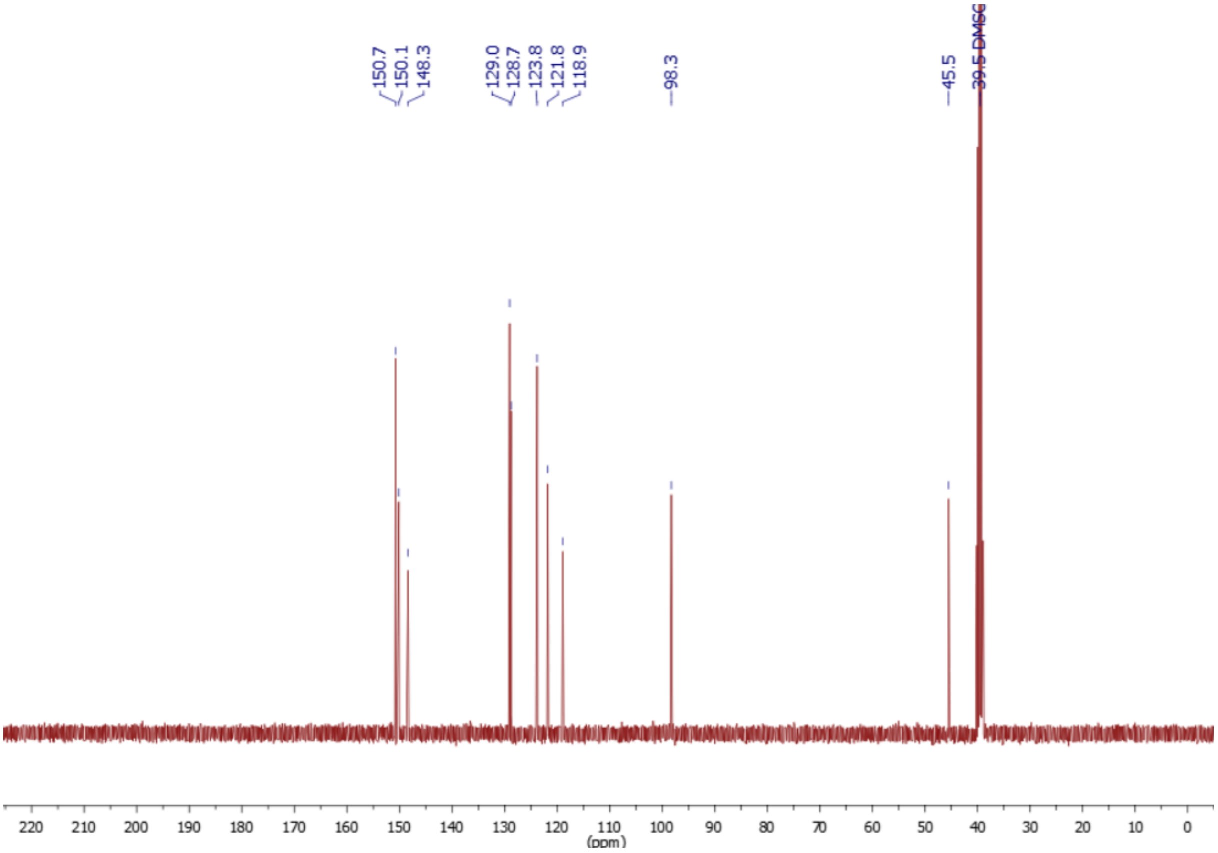

***N*[1]-(7-fluoroquinolin-4-yl)ethane-1,2-diamine (13)**

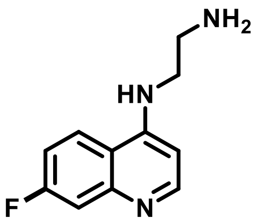

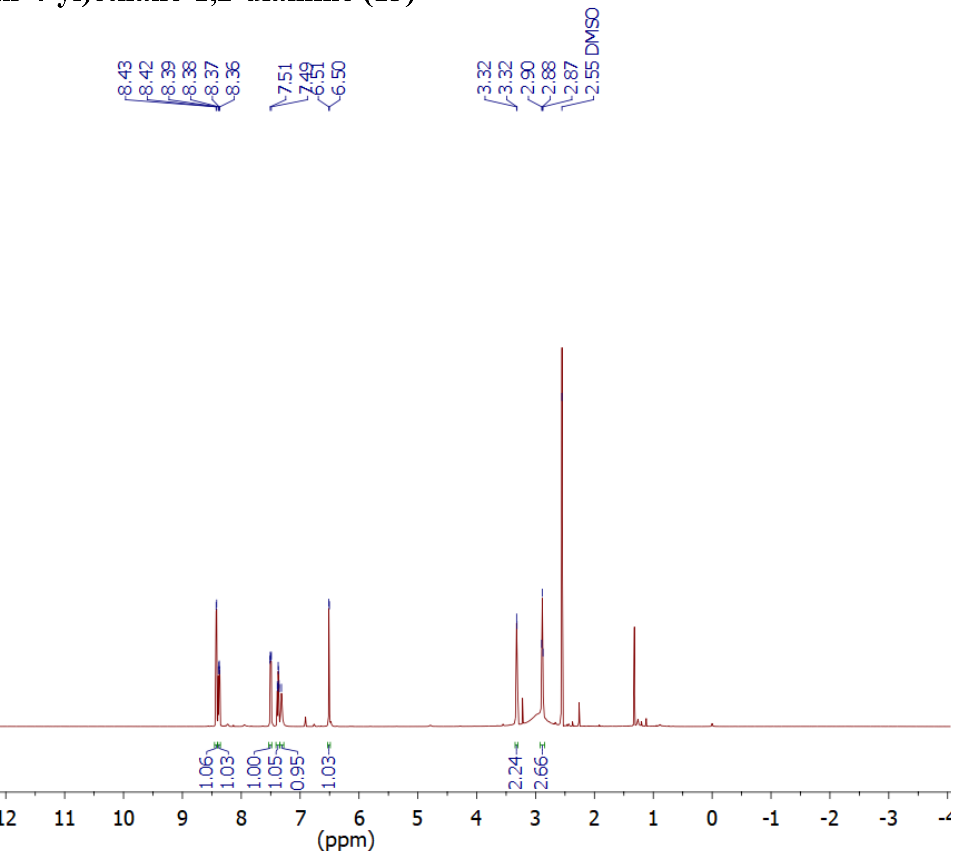

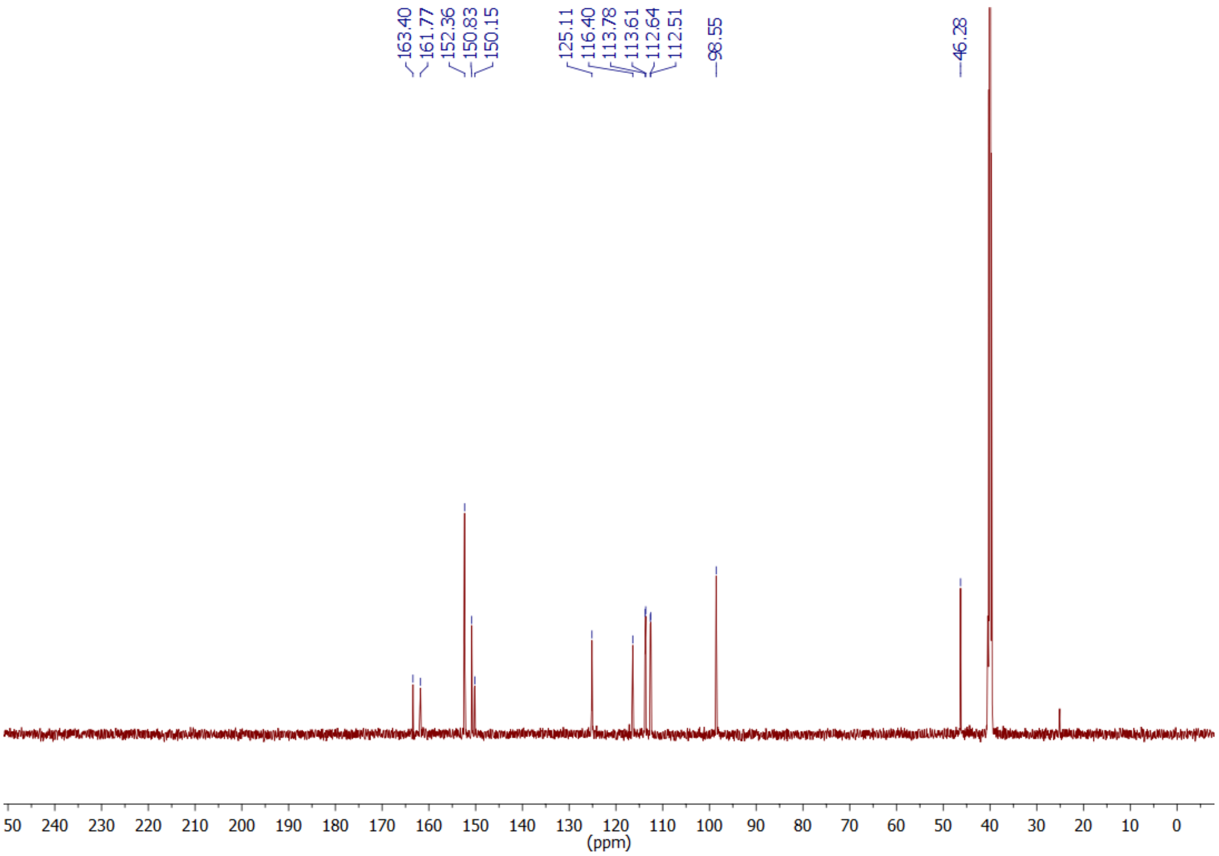

***N*[1]-(7-methoxyquinolin-4-yl)ethane-1,2-diamine (14)**

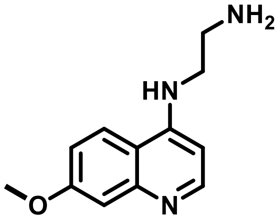

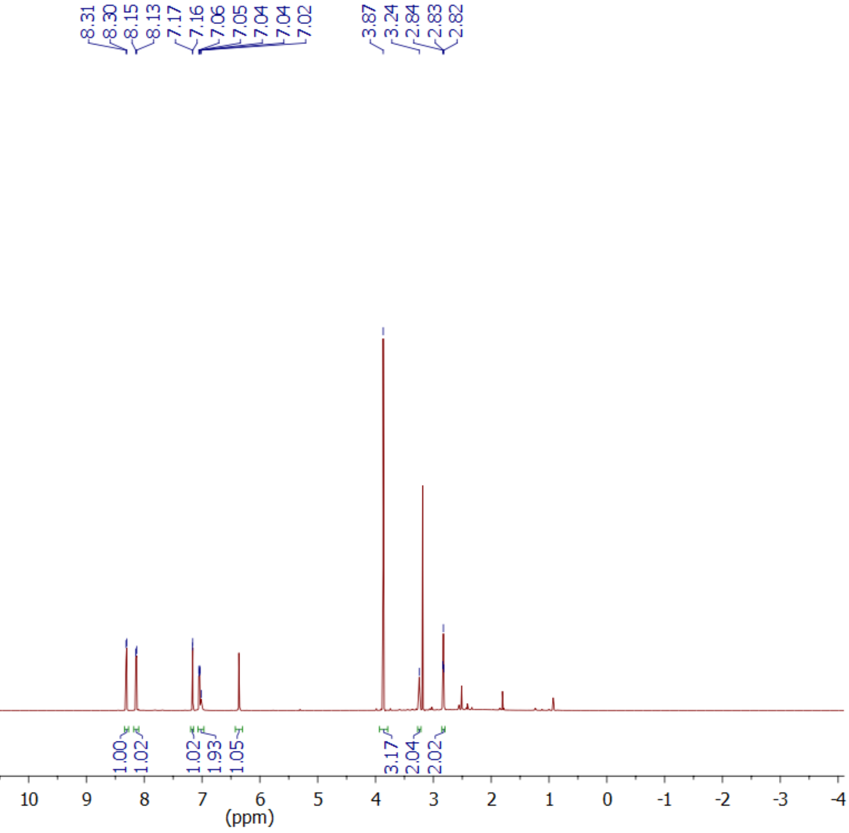

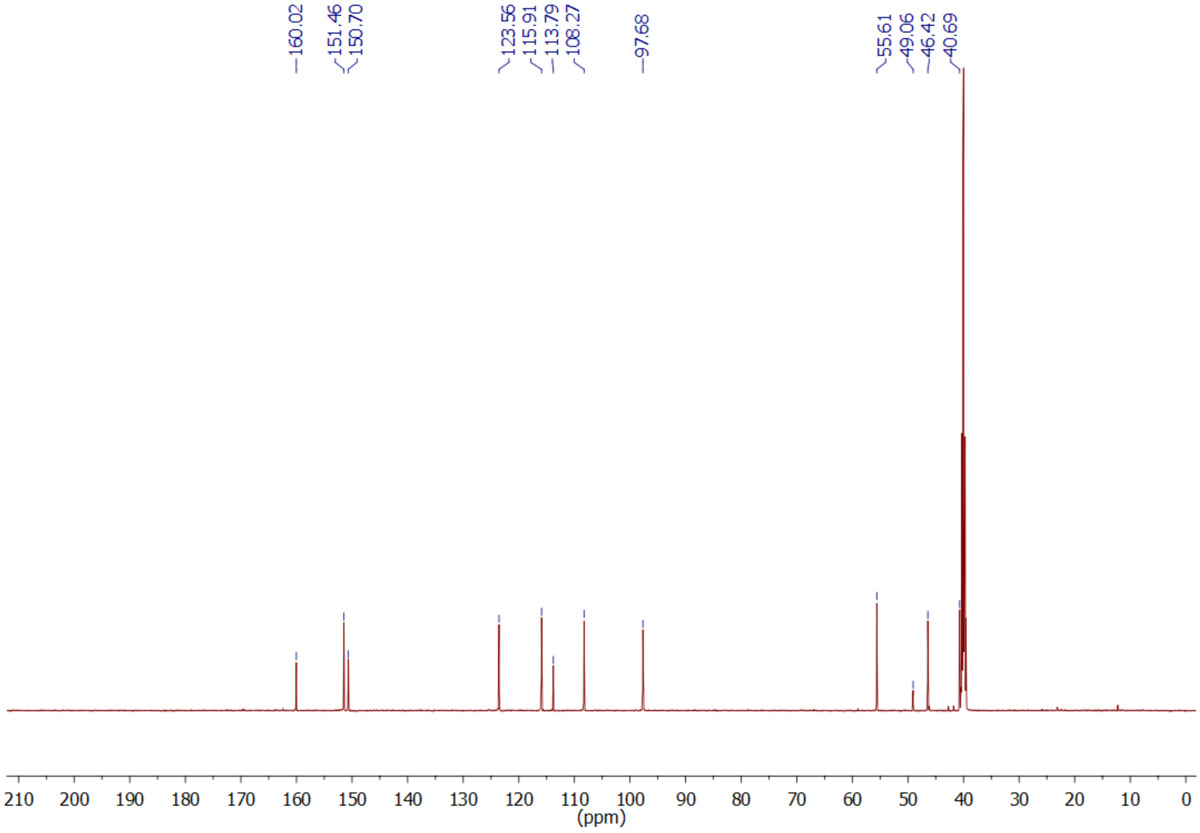

N^1-(7-phenoxyquinolin-4-yl)ethane-1,2-diamine (15)

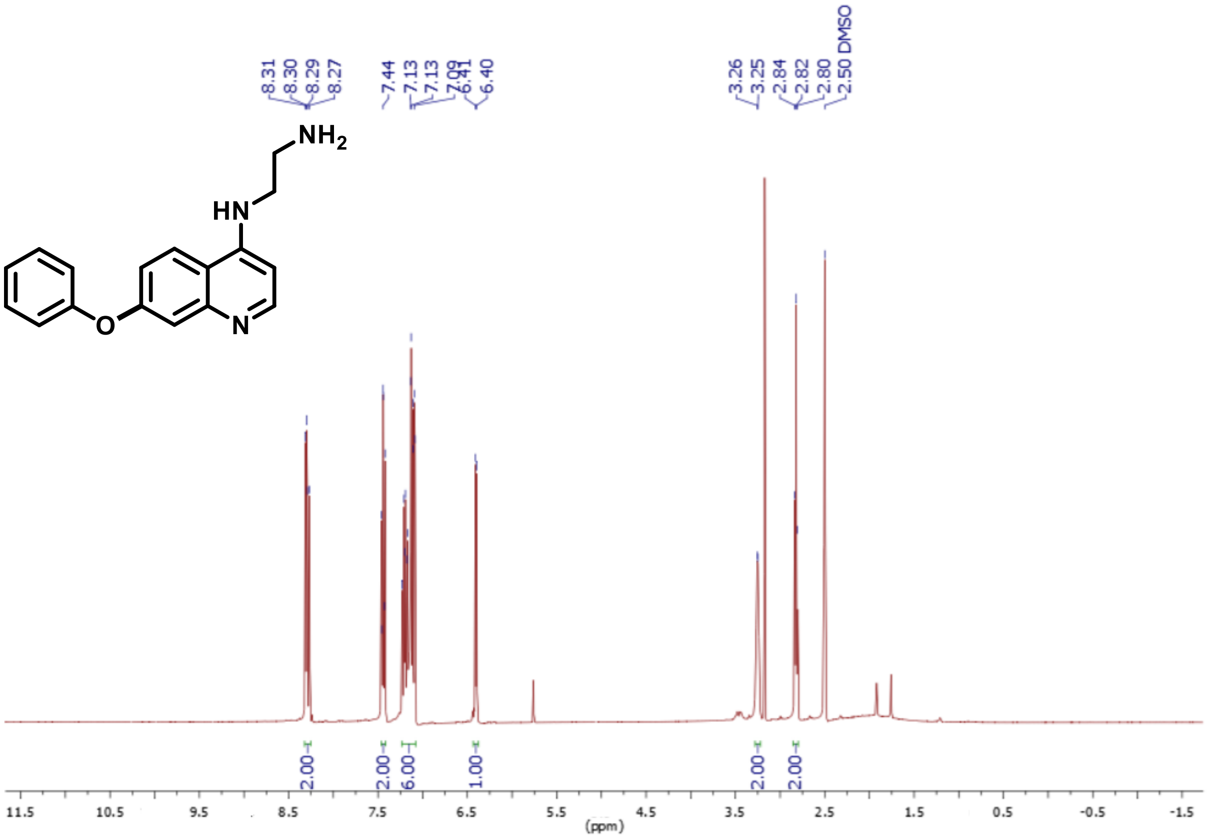

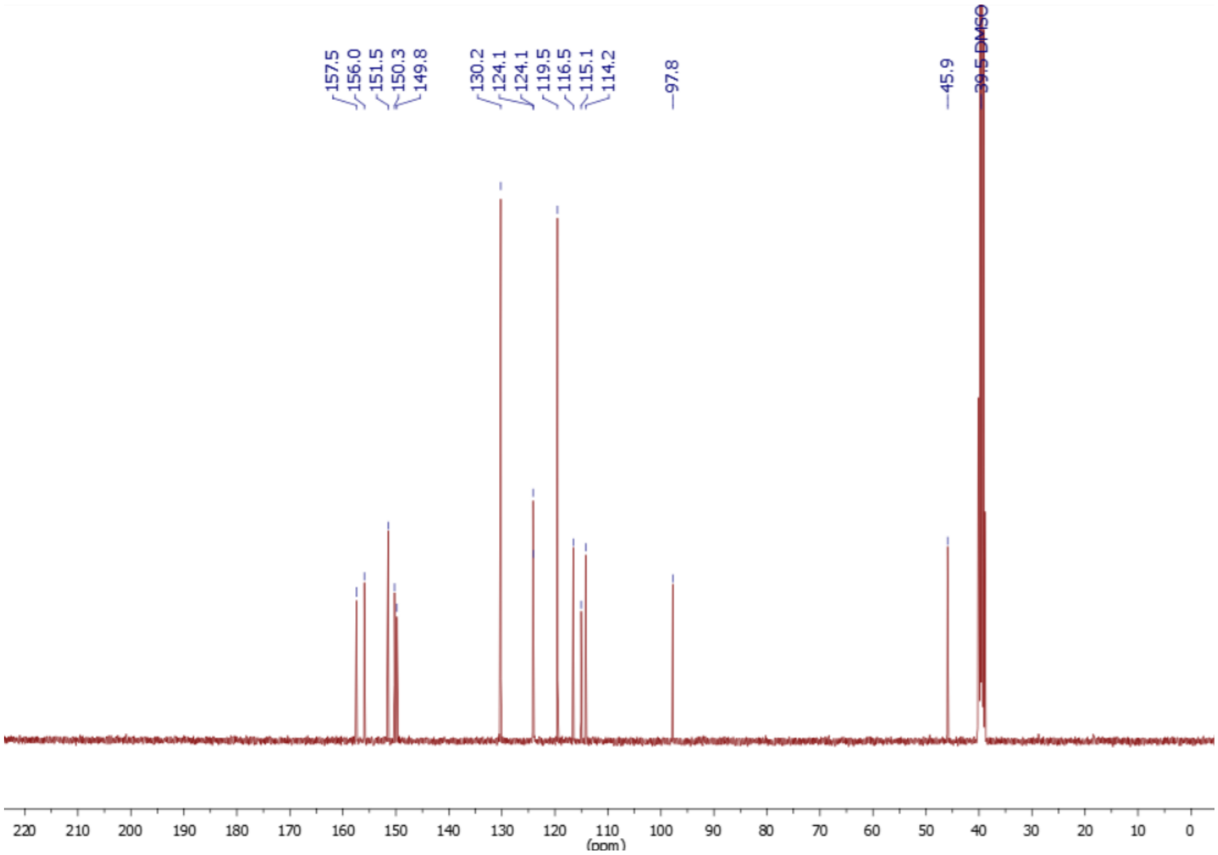

***N*[1]-[7-(benzyloxy)quinolin-4-yl]ethane-1,2-diamine (16)**

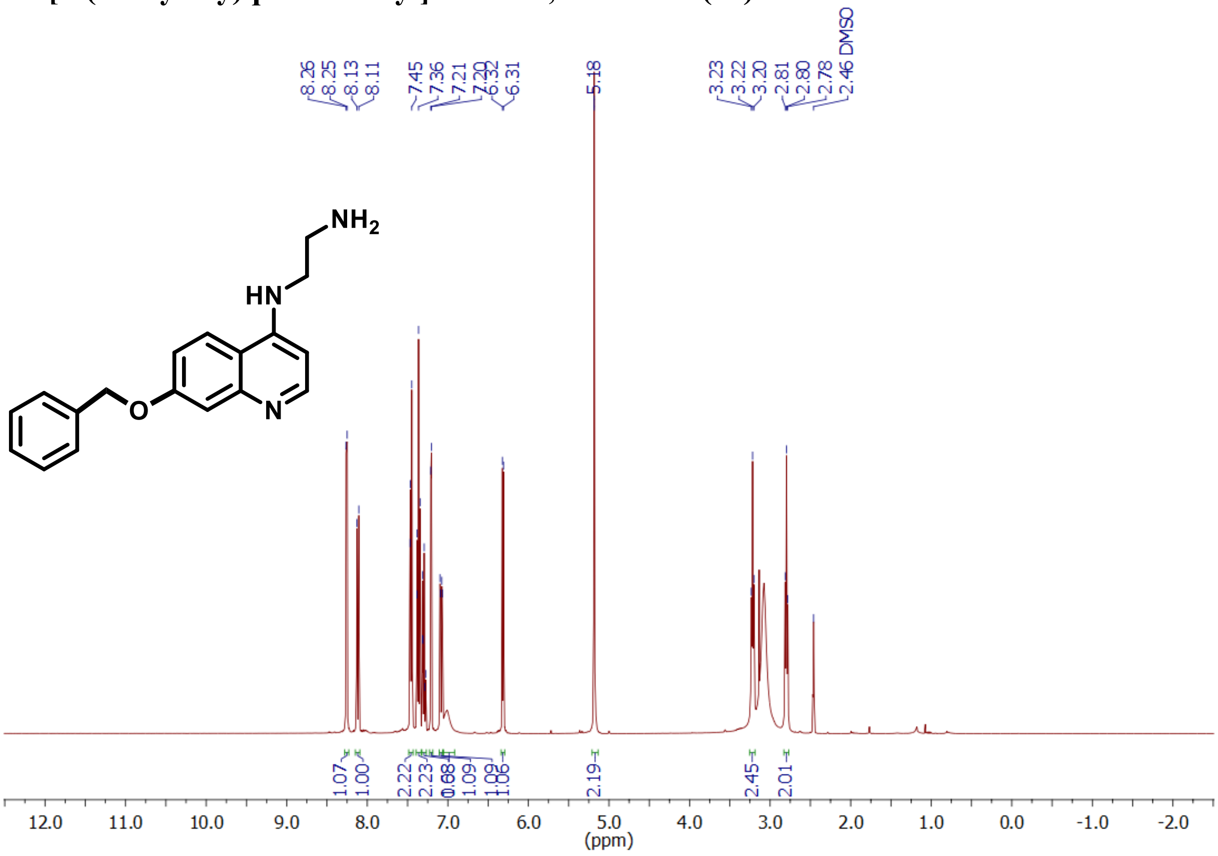

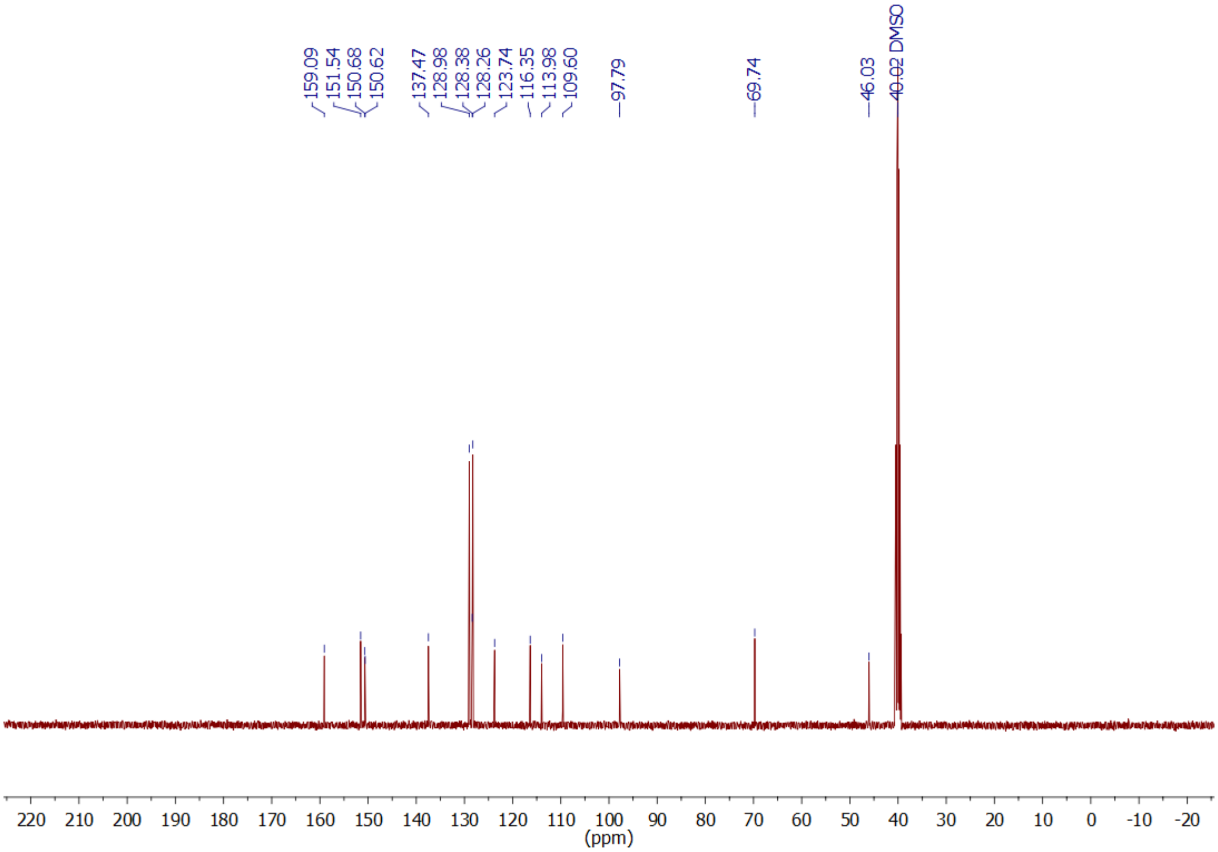

N[1]-(8-chloroquinolin-4-yl)ethane-1,2-diamine (17)

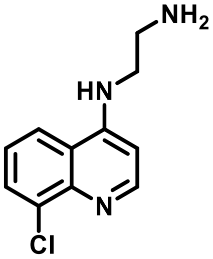

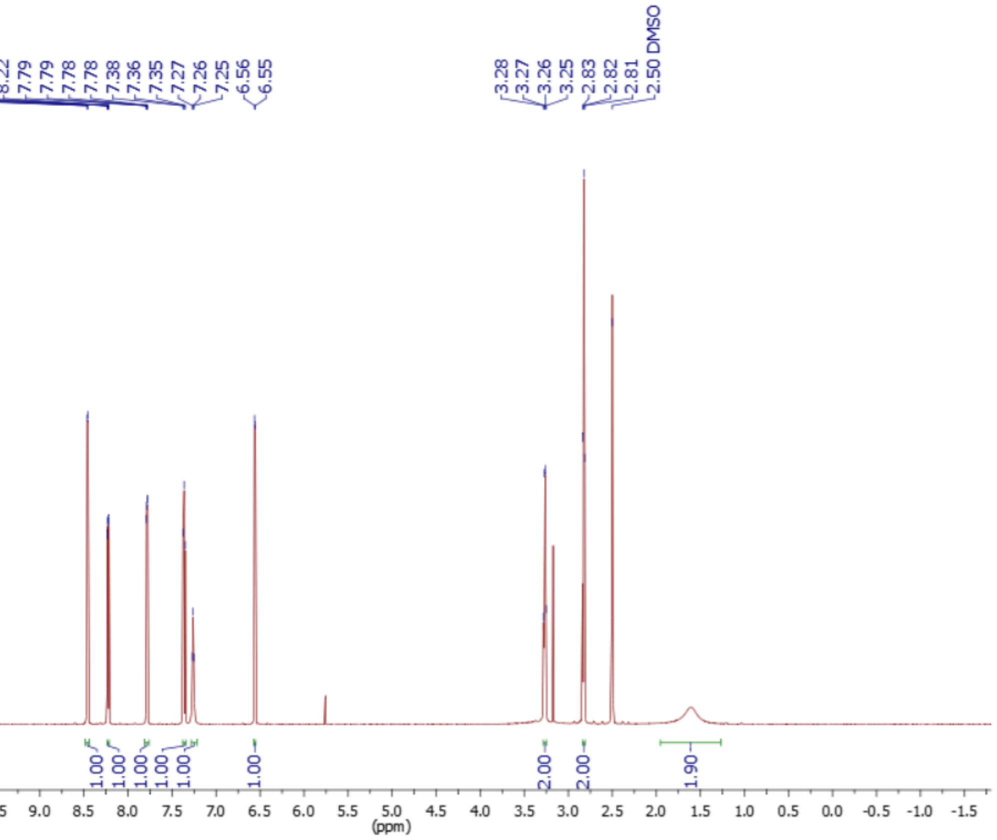

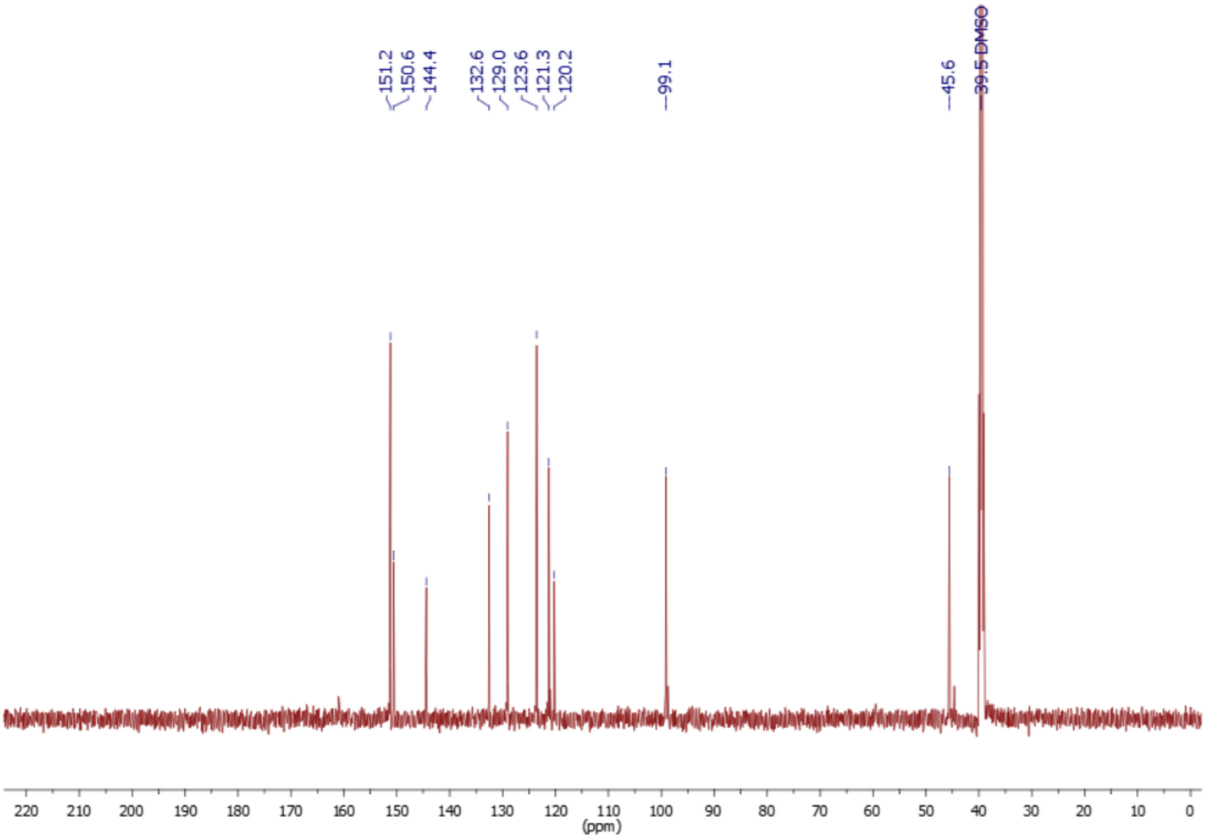

N^1-(8-fluoroquinolin-4-yl)ethane-1,2-diamine (18)

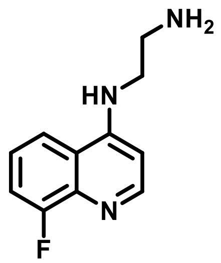

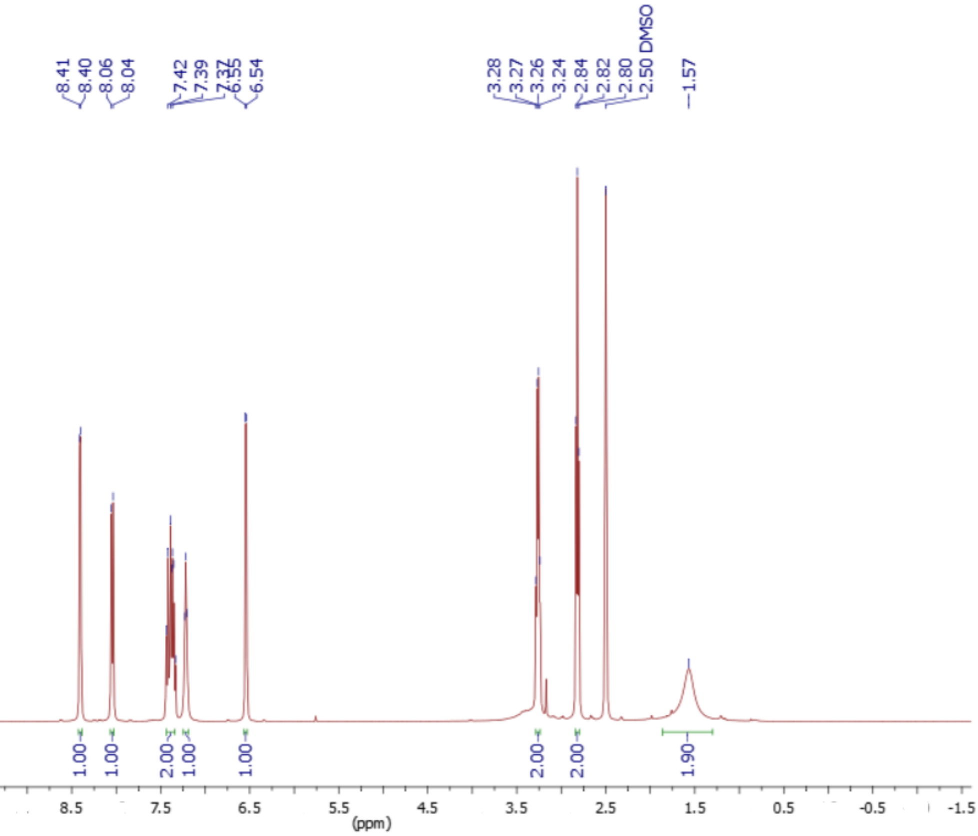

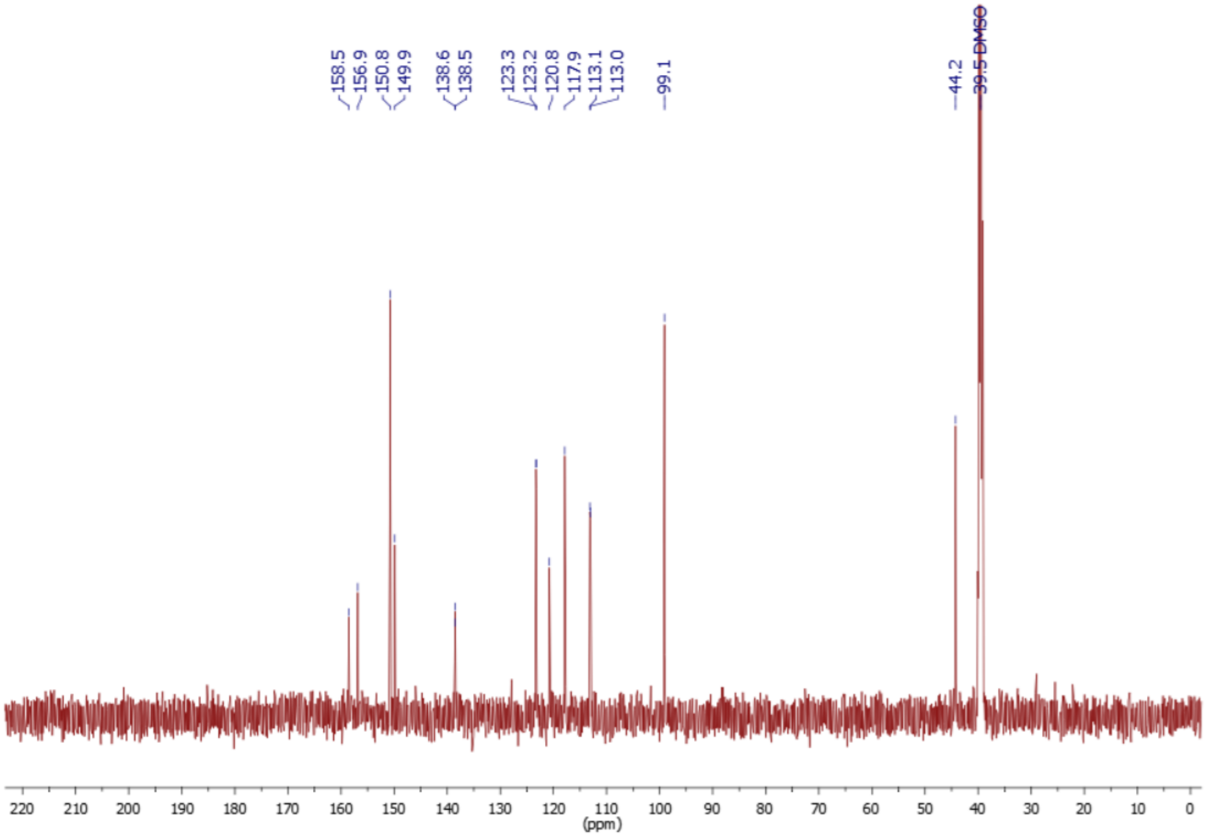

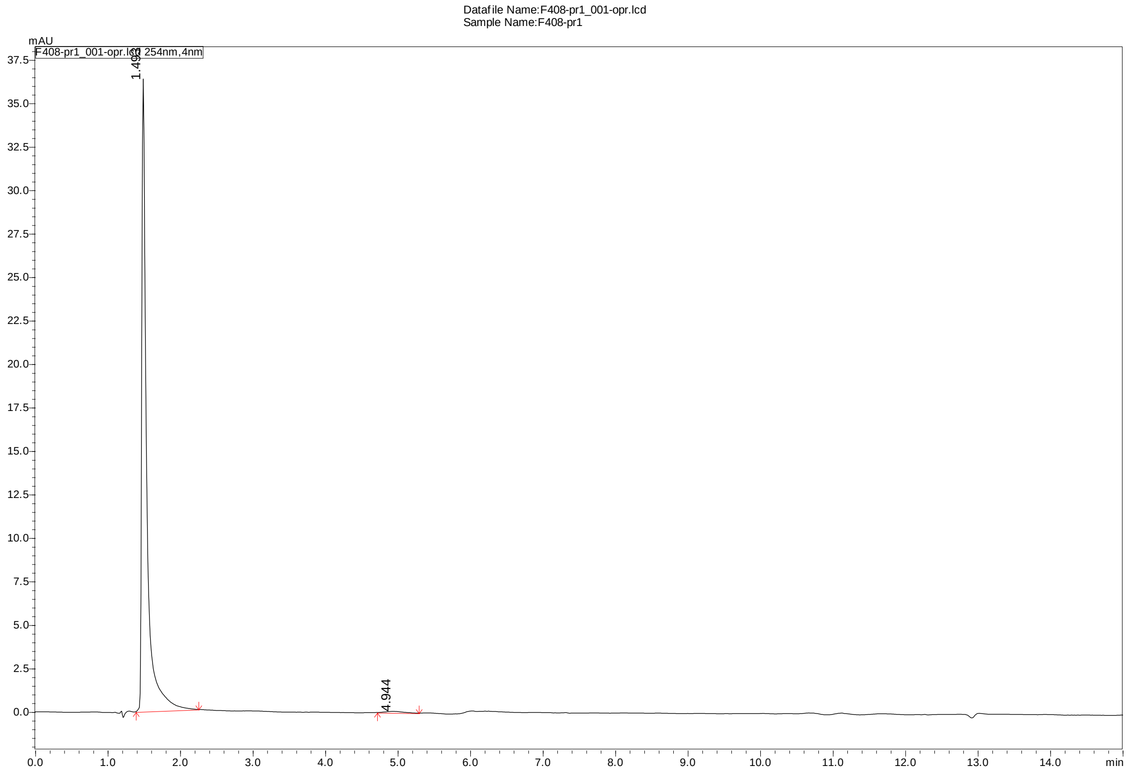

**Sample**	**F408**		222.25 m/z	
	Time	Height	Area	Area%
1.	1.493	36384	172911	99.256
2.	4.944	81	1296	0.744
Total		36465	174207	100.000

Mass Spectrum List Report

Analysis Info

Analysis Name	D:\Data\Zlecenia\2023-04-26\F408_000001.d	Acquisition Date	4/26/2023 11:56:15 AM
Method	tune_low.m	Operator	BDAL@DE
Sample Name		Instrument / Ser#	micrOTOF 213750.10372
Comment	MeOH		

Acquisition Parameter

Source Type	ESI	Ion Polarity	Positive	Set Nebulizer	0.4 Bar
Focus	Not active			Set Dry Heater	180 °C
Scan Begin	50 m/z	Set Capillary	4500 V	Set Dry Gas	4.0 l/min
Scan End	3000 m/z	Set End Plate Offset	-500 V	Set Divert Valve	Waste

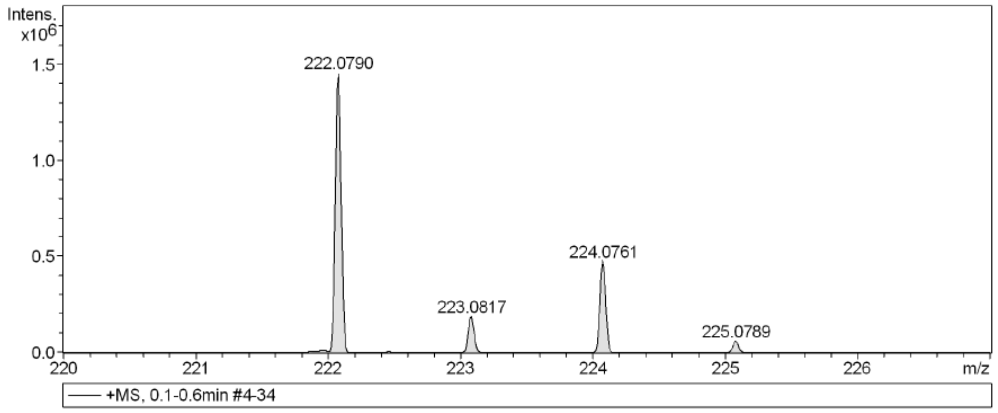

Datafile Name:SRK2_008-opr.lcd
Sample Name:SRK2

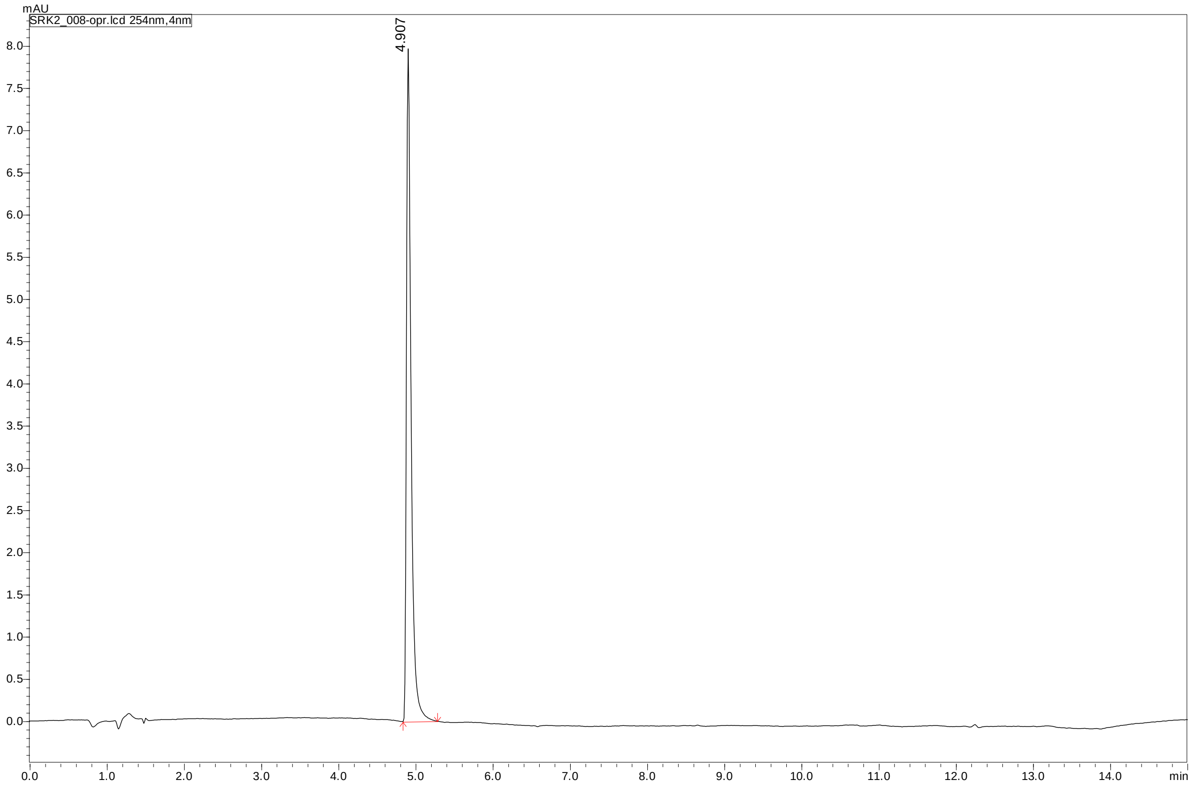

Sample	1		223.25 m/z	
	Time	Height	Area	Area%
1.	4.907	7969	33338	100.000
Total		7969	33338	100.000

Mass Spectrum List Report

Analysis Info

Analysis Name	D:\Data\Zlecenia\2023_03_22\SRK2_000001.d	Acquisition Date	3/22/2023 11:12:05 AM
Method	tune_low.m	Operator	BDAL@DE
Sample Name		Instrument / Ser#	micrOTOF 213750.10372
Comment	MeOH		

Acquisition Parameter

Source Type	ESI	Ion Polarity	Positive	Set Nebulizer	0.4 Bar
Focus	Not active			Set Dry Heater	180 °C
Scan Begin	50 m/z	Set Capillary	4500 V	Set Dry Gas	4.0 l/min
Scan End	3000 m/z	Set End Plate Offset	-500 V	Set Divert Valve	Waste

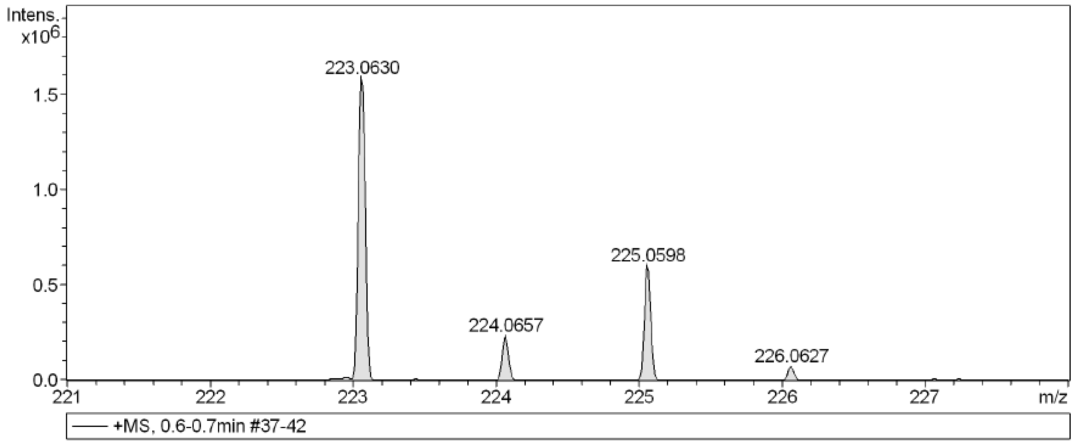

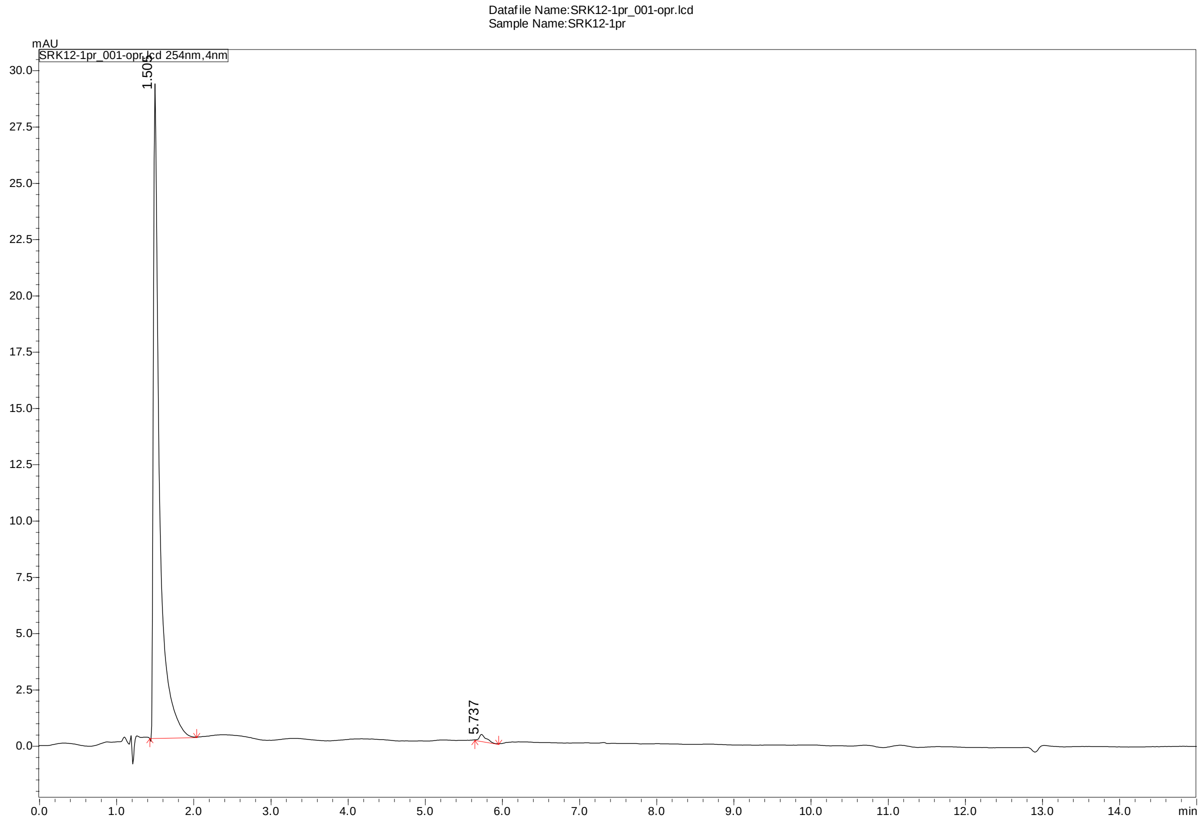

Sample	2		250.20 m/z	
	Time	Height	Area	Area%
1.	1.505	29047	159511	98.964
2.	5.737	297	1670	1.036
Total		29344	161181	100.000

Mass Spectrum List Report

Analysis Info

Analysis Name	D:\Data\Zlecenia\2023_03_22\SRK12_000001.d	Acquisition Date	3/22/2023 11:51:53 AM
Method	tune_low.m	Operator	BDAL@DE
Sample Name		Instrument / Ser#	micrOTOF 213750.10372
Comment	MeOH		

Acquisition Parameter

Source Type	ESI	Ion Polarity	Positive	Set Nebulizer	0.4 Bar
Focus	Not active			Set Dry Heater	180 °C
Scan Begin	50 m/z	Set Capillary	4500 V	Set Dry Gas	4.0 l/min
Scan End	3000 m/z	Set End Plate Offset	-500 V	Set Divert Valve	Waste

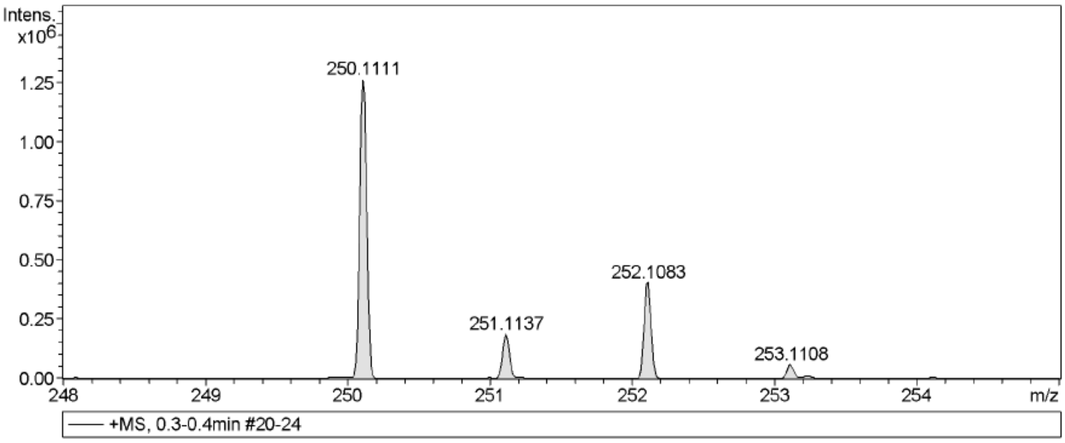

Datafile Name:SKR176-1pr_003-opr.lcd
Sample Name:SKR176-1pr

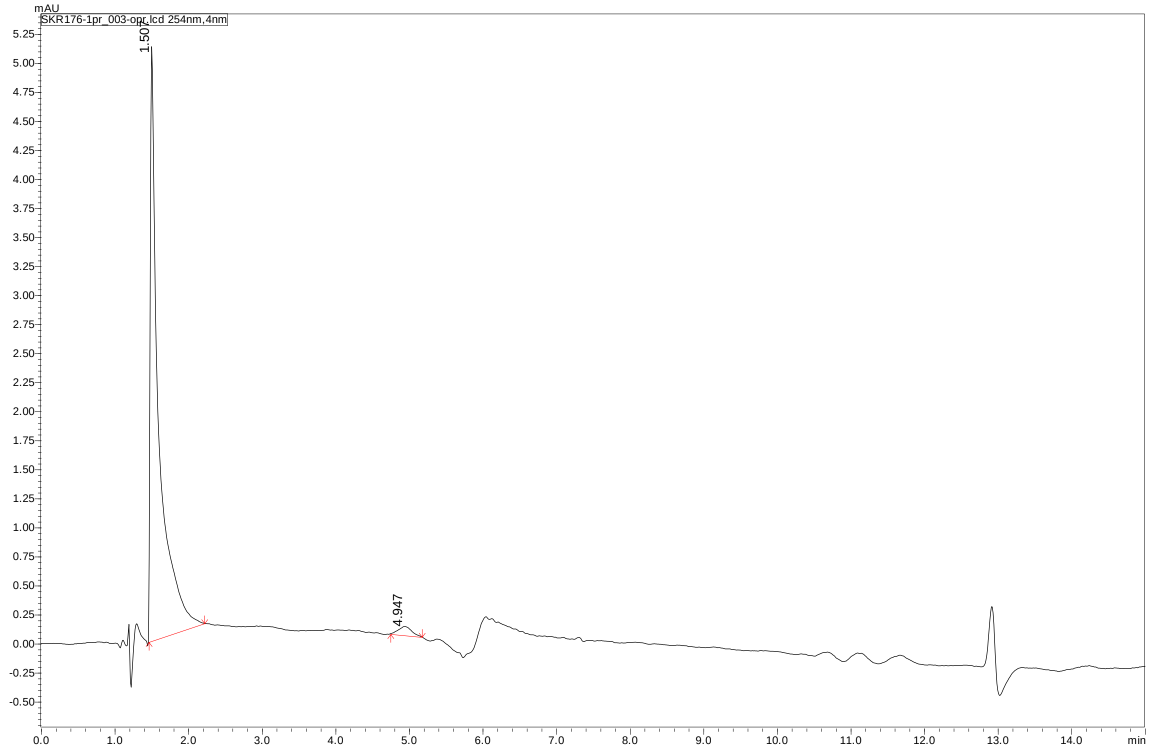

Sample	3		248.30 m/z	
	Time	Height	Area	Area%
1.	1.507	5117	41419	97.846
2.	4.947	75	912	2.154
Total		5192	42331	100.000

Mass Spectrum List Report

Analysis Info

Analysis Name	D:\Data\Zlecenia\2023_03_22\SRK176_000002.d	Acquisition Date	3/22/2023 12:59:23 PM
Method	tune_low.m	Operator	BDAL@DE
Sample Name		Instrument / Ser#	micrOTOF 213750.10372
Comment	MeOH		

Acquisition Parameter

Source Type	ESI	Ion Polarity	Positive	Set Nebulizer	0.4 Bar
Focus	Not active			Set Dry Heater	180 °C
Scan Begin	50 m/z	Set Capillary	4500 V	Set Dry Gas	4.0 l/min
Scan End	3000 m/z	Set End Plate Offset	-500 V	Set Divert Valve	Waste

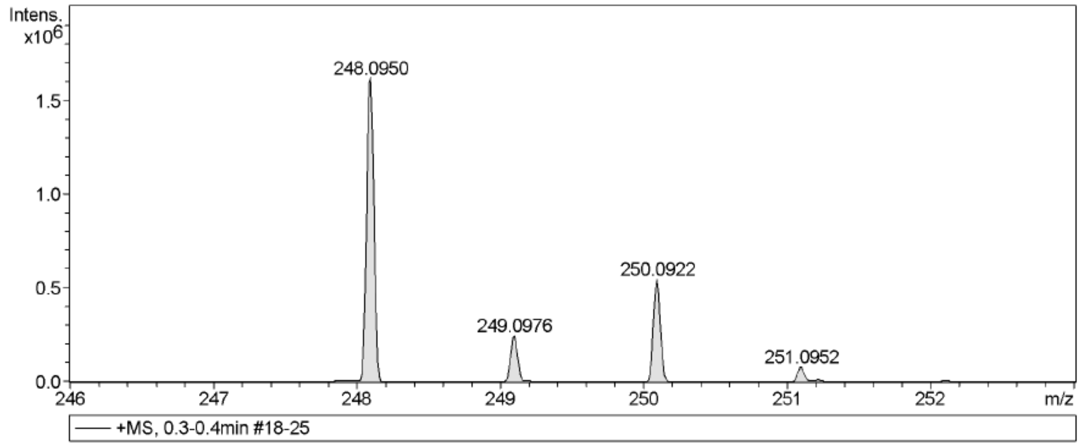

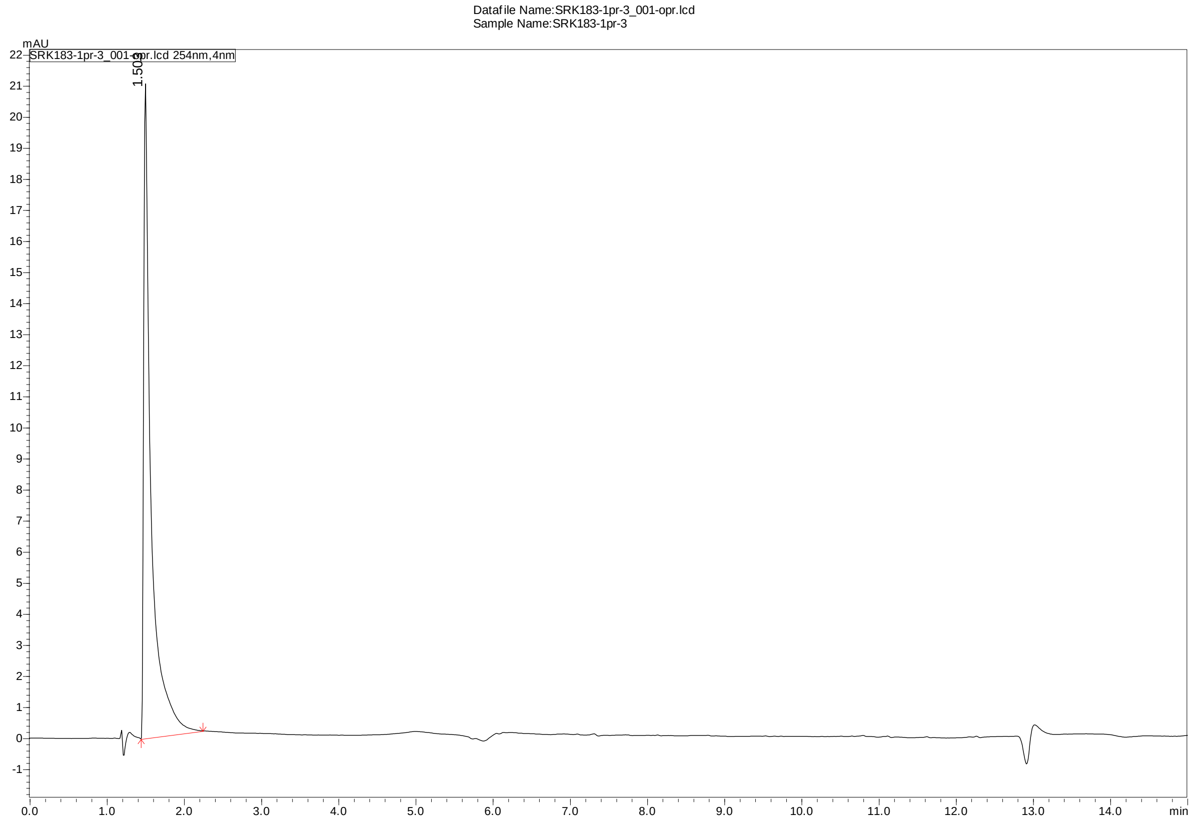

Sample	4		248.30 m/z	
	Time	Height	Area	Area%
1.	1.503	21065	139919	100.000
Total		21065	139919	100.000

Mass Spectrum List Report

Analysis Info

Analysis Name	D:\Data\Zlecenia\2023_03_22\SRK183_000001.d	Acquisition Date	3/22/2023 3:14:20 PM
Method	tune_low.m	Operator	BDAL@DE
Sample Name		Instrument / Ser#	micrOTOF 213750.10372
Comment	MeOH		

Acquisition Parameter

Source Type	ESI	Ion Polarity	Positive	Set Nebulizer	0.4 Bar
Focus	Not active			Set Dry Heater	180 °C
Scan Begin	50 m/z	Set Capillary	4500 V	Set Dry Gas	4.0 l/min
Scan End	3000 m/z	Set End Plate Offset	-500 V	Set Divert Valve	Waste

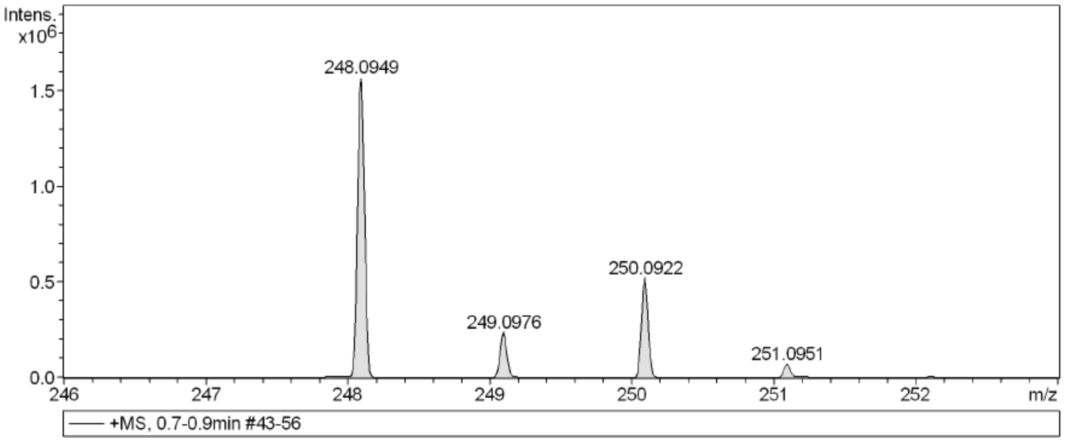

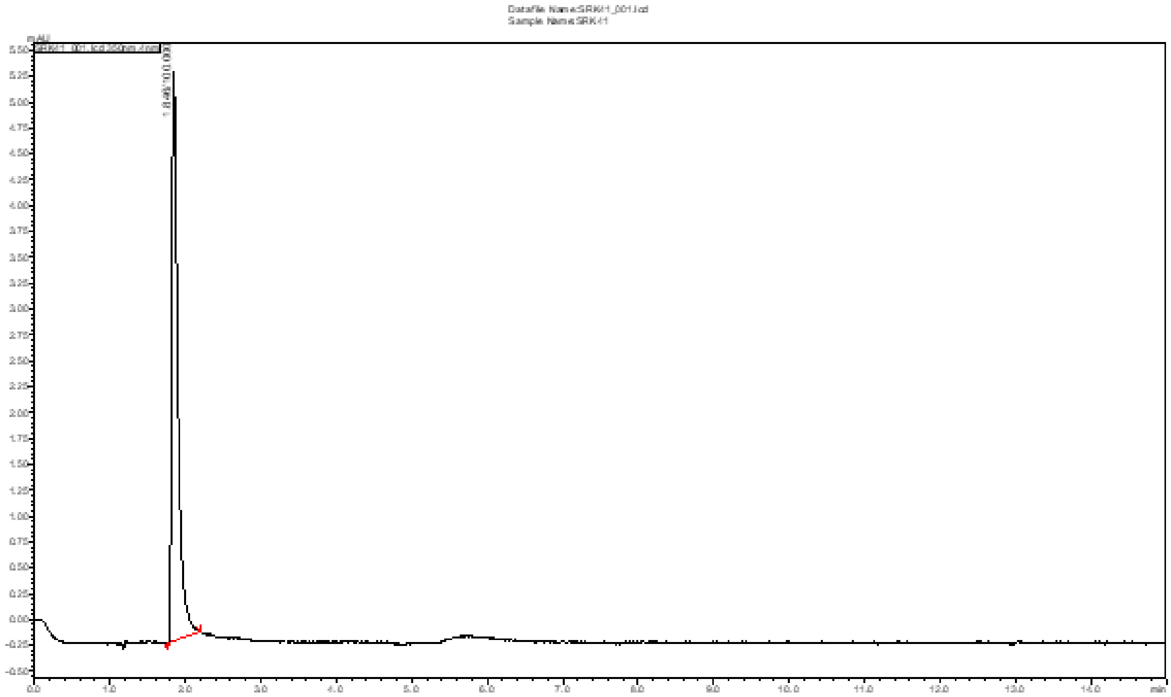

Sample	6		262.20 m/z	
	Time	Height	Area	Area%
1.	1.846	5486	30813	100.000
Total		5486	30813	100.000

Mass Spectrum List Report

Analysis Info

Analysis Name	D:\Data\Zlecenia\2023_07_31\SRK41_000001.d	Acquisition Date	7/31/2023 10:49:11 AM
Method	tune_low.m	Operator	BDAL@DE
Sample Name		Instrument / Ser#	micrOTOF 213750.10372
Comment	MeOH		

Acquisition Parameter

Source Type	ESI	Ion Polarity	Positive	Set Nebulizer	0.4 Bar
Focus	Not active			Set Dry Heater	180 °C
Scan Begin	50 m/z	Set Capillary	4500 V	Set Dry Gas	4.0 l/min
Scan End	3000 m/z	Set End Plate Offset	-500 V	Set Divert Valve	Waste

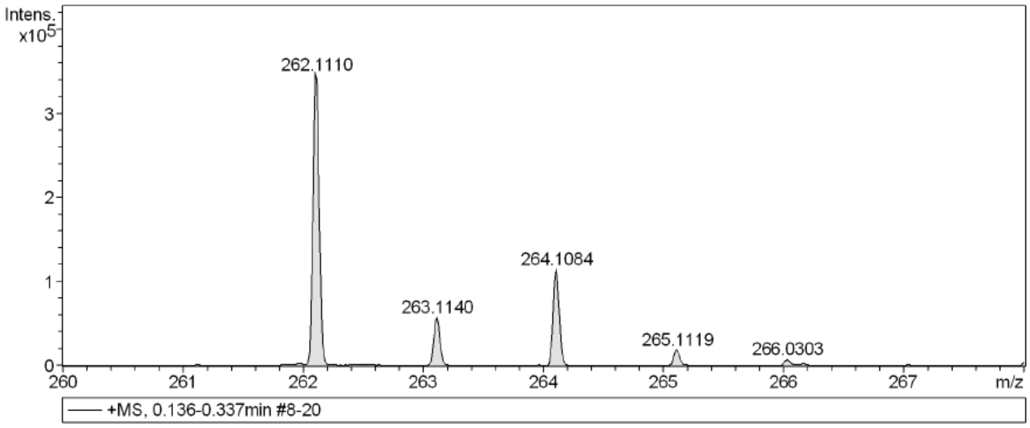

Datafile Name:SRK54_001-opr.lcd
Sample Name:SRK54

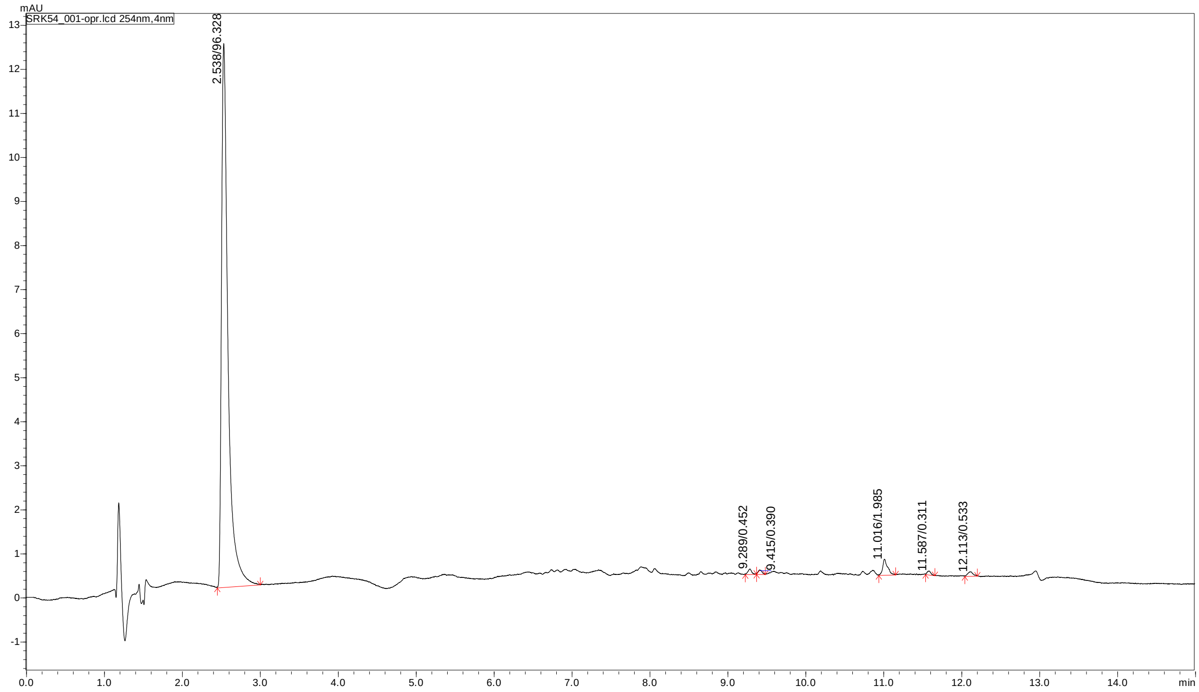

Sample	7		262.15 m/z	
	Time	Height	Area	Area%
1.	2.538	12270	71450	96.328
2.	9.289	109	335	0.452
3.	9.415	88	290	0.390
4.	11.016	347	1473	1.985
5.	11.587	79	231	0.311
6.	12.113	89	395	0.533
Total		12982	74173	100.000

Mass Spectrum List Report

Analysis Info

Analysis Name	D:\Data\Zlecenia\2023_05_10\SRK54_000001.d	Acquisition Date	5/10/2023 1:22:52 PM
Method	tune_low.m	Operator	BDAL@DE
Sample Name		Instrument / Ser#	micrOTOF 213750.10372
Comment	MeOH		

Acquisition Parameter

Source Type	ESI	Ion Polarity	Positive	Set Nebulizer	0.4 Bar
Focus	Not active			Set Dry Heater	180 °C
Scan Begin	50 m/z	Set Capillary	4500 V	Set Dry Gas	4.0 l/min
Scan End	3000 m/z	Set End Plate Offset	-500 V	Set Divert Valve	Waste

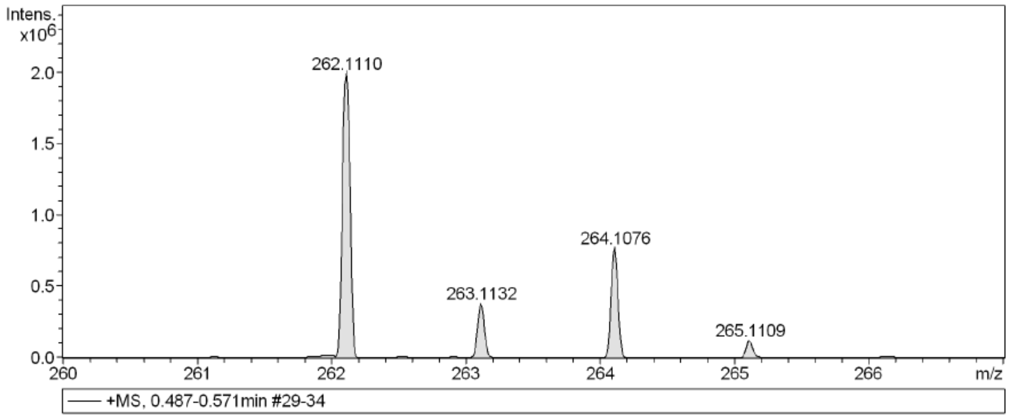

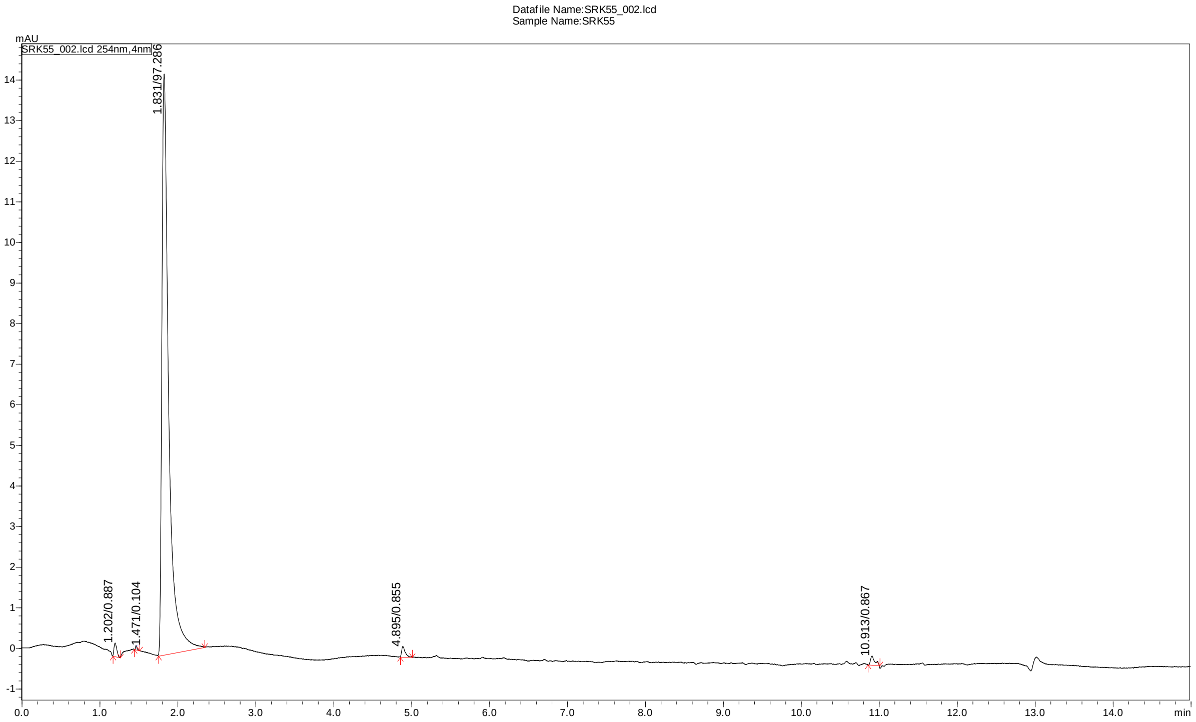

Sample	8		262.20 m/z	
	Time	Height	Area	Area%
1.	1.202	318	791	0.887
2.	1.471	92	93	0.104
3.	1.831	14238	86729	97.286
4.	4.895	256	762	0.855
5.	10.913	209	773	0.867
Total		15113	89148	100.000

Mass Spectrum List Report

Analysis Info

Analysis Name	D:\Data\Zlecenia\2023_07_31\SRK55_000001.d	Acquisition Date	7/31/2023 11:36:49 AM	
Method	tune_low.m	Operator	BDAL@DE	
Sample Name		Instrument / Ser#	micrOTOF	213750.10372
Comment	MeOH			

Acquisition Parameter

Source Type	ESI	Ion Polarity	Positive	Set Nebulizer	0.4 Bar
Focus	Not active			Set Dry Heater	180 °C
Scan Begin	50 m/z	Set Capillary	4500 V	Set Dry Gas	4.0 l/min
Scan End	3000 m/z	Set End Plate Offset	-500 V	Set Divert Valve	Waste

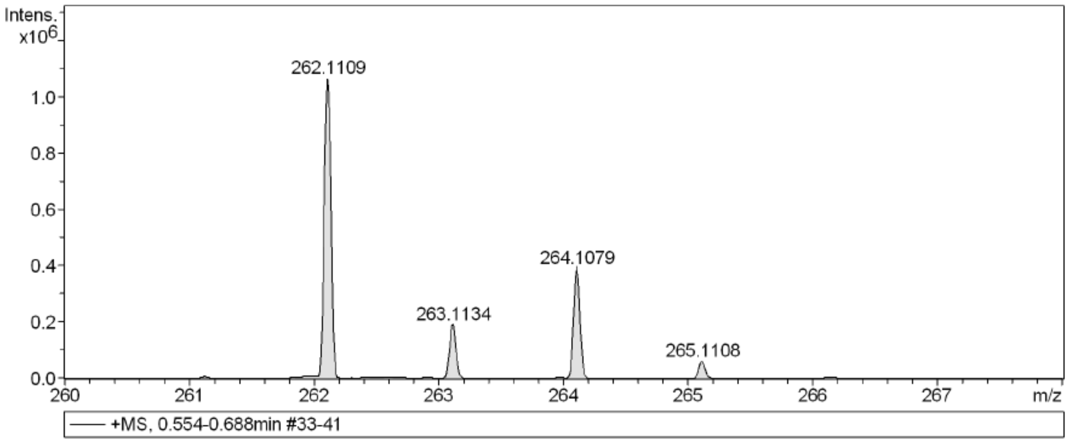

Datafile Name:SRK1-2_002.lcd
Sample Name:SRK1-2

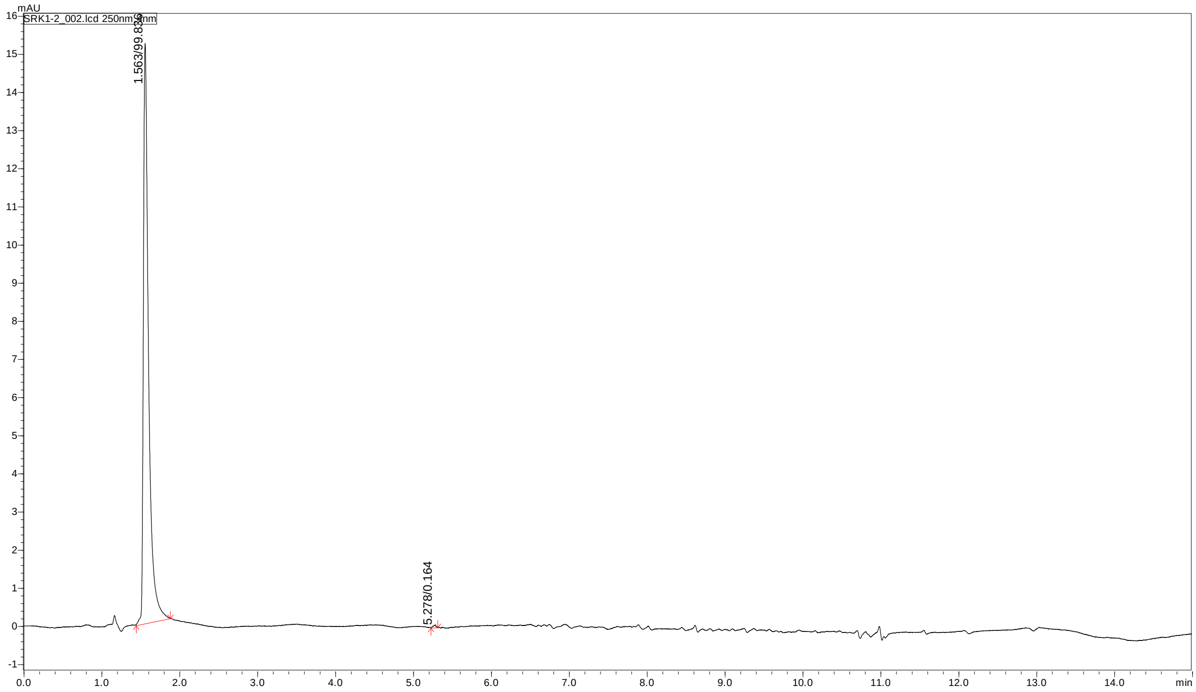

Sample	9		256.20 m/z	
	Time	Height	Area	Area%
1.	1.563	15077	70266	99.836
2.	5.278	55	116	0.164
Total		15132	70382	100.000

Mass Spectrum List Report

Analysis Info

Analysis Name	D:\Data\Zlecenia\2023_03_22\SRK1_000001.d	Acquisition Date	3/22/2023 10:58:49 AM
Method	tune_low.m	Operator	BDAL@DE
Sample Name		Instrument / Ser#	micrOTOF 213750.10372
Comment	MeOH		

Acquisition Parameter

Source Type	ESI	Ion Polarity	Positive	Set Nebulizer	0.4 Bar
Focus	Not active			Set Dry Heater	180 °C
Scan Begin	50 m/z	Set Capillary	4500 V	Set Dry Gas	4.0 l/min
Scan End	3000 m/z	Set End Plate Offset	-500 V	Set Divert Valve	Waste

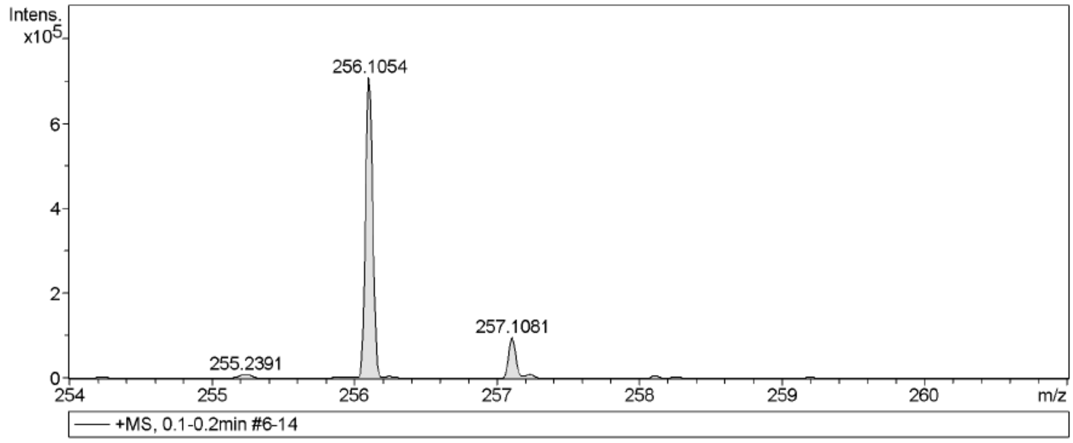

Datafile Name:SRK182-pr1_004-opr.lcd
Sample Name:SRK182-pr1

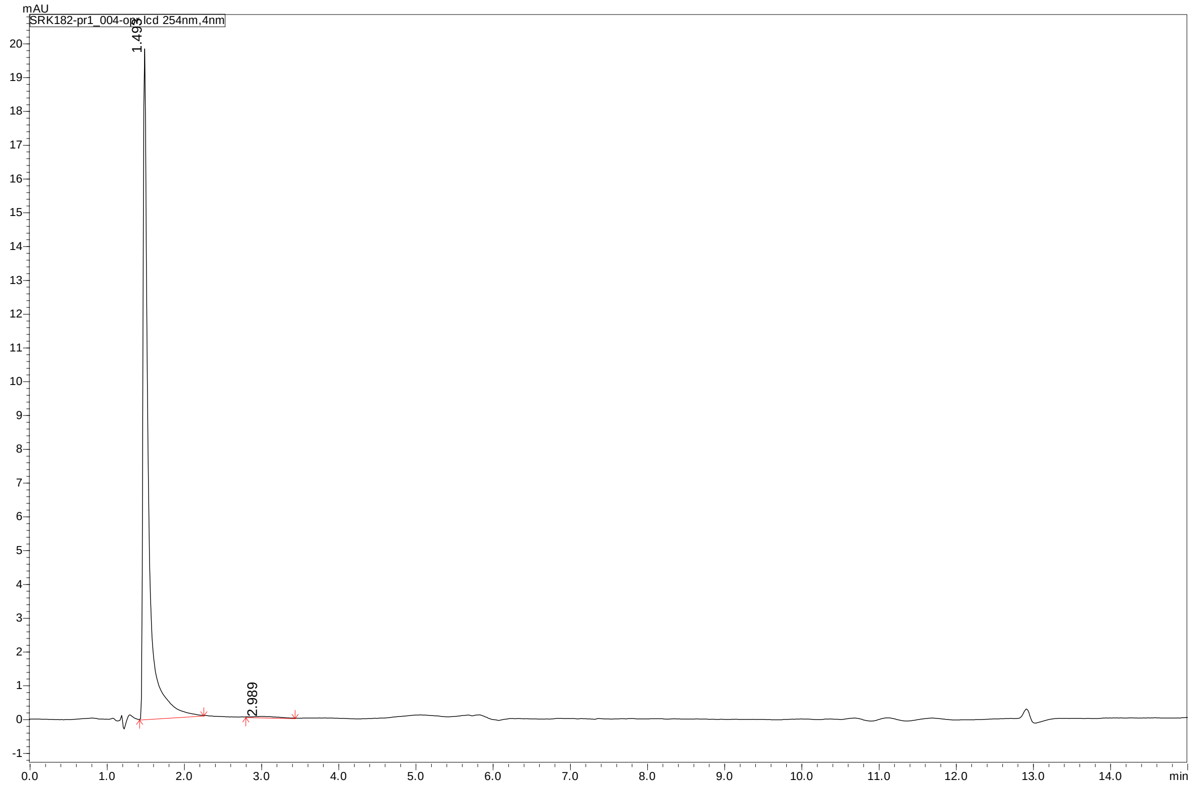

Sample	10		202.35 m/z	
	Time	Height	Area	Area%
1.	1.493	19839	95987	99.472
2.	2.989	26	509	0.528
Total		19865	96496	100.000

Mass Spectrum List Report

Analysis Info

Analysis Name	D:\Data\Zlecenia\2023_03_22\SRK182_000001.d	Acquisition Date	3/22/2023 3:09:38 PM
Method	tune_low.m	Operator	BDAL@DE
Sample Name		Instrument / Ser#	micrOTOF 213750.10372
Comment	MeOH		

Acquisition Parameter

Source Type	ESI	Ion Polarity	Positive	Set Nebulizer	0.4 Bar
Focus	Not active			Set Dry Heater	180 °C
Scan Begin	50 m/z	Set Capillary	4500 V	Set Dry Gas	4.0 l/min
Scan End	3000 m/z	Set End Plate Offset	-500 V	Set Divert Valve	Waste

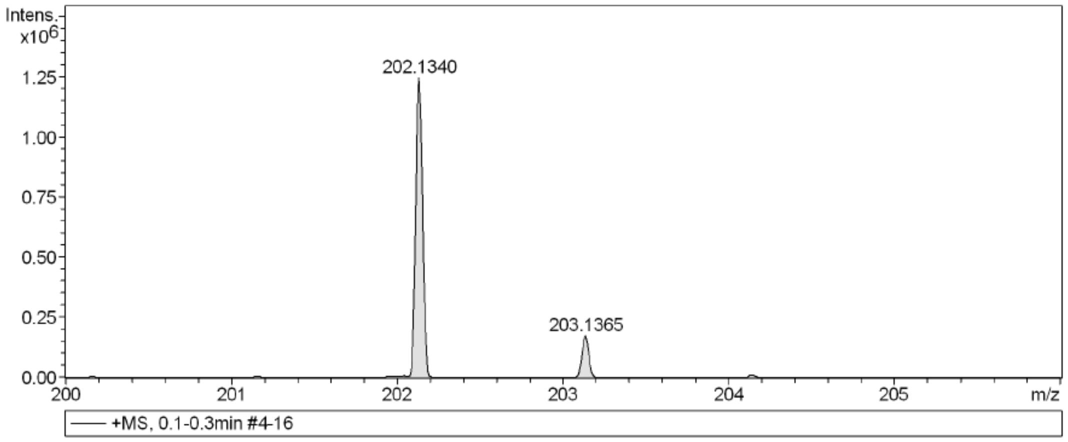

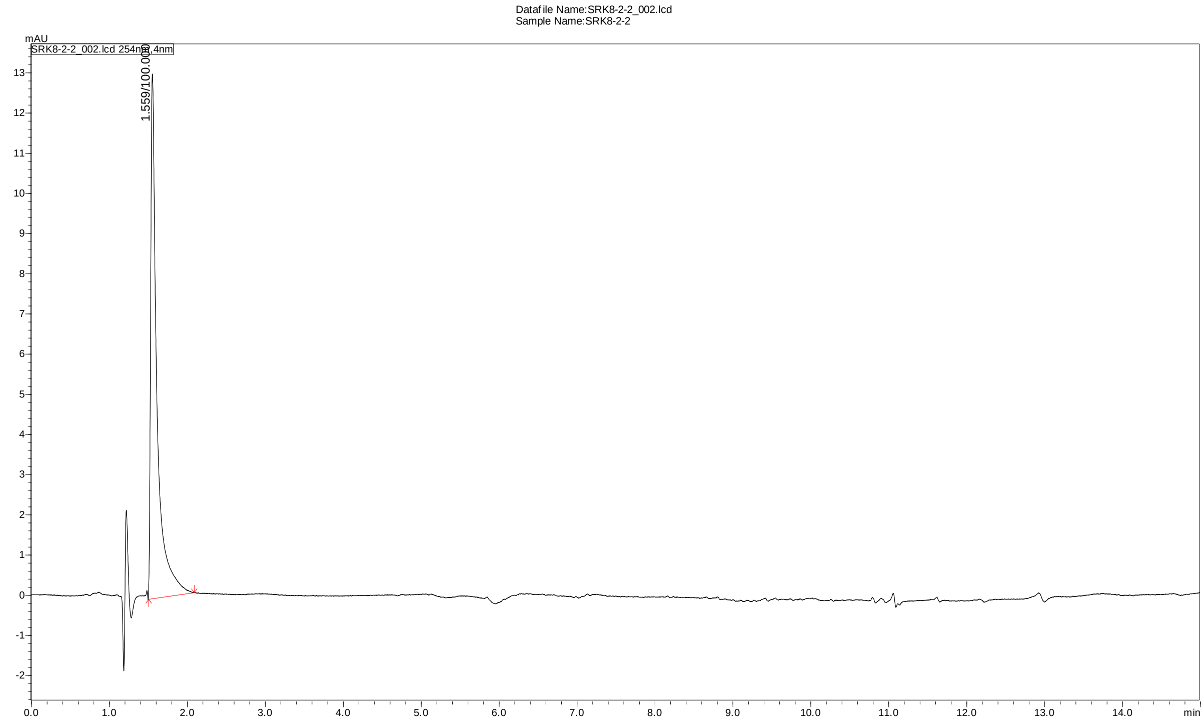

Sample	11		266.10 m/z	
	Time	Height	Area	Area%
1.	1.559	12973	73196	100.000
Total		12973	73196	100.000

Mass Spectrum List Report

Analysis Info

Analysis Name	D:\Data\Zlecenia\2023_07_31\SRK8_2_000001.d	Acquisition Date	7/31/2023 10:19:58 AM	
Method	tune_low.m	Operator	BDAL@DE	
Sample Name		Instrument / Ser#	micrOTOF	213750.10372
Comment	MeOH			

Acquisition Parameter

Source Type	ESI	Ion Polarity	Positive	Set Nebulizer	0.4 Bar
Focus	Not active			Set Dry Heater	180 °C
Scan Begin	50 m/z	Set Capillary	4500 V	Set Dry Gas	4.0 l/min
Scan End	3000 m/z	Set End Plate Offset	-500 V	Set Divert Valve	Waste

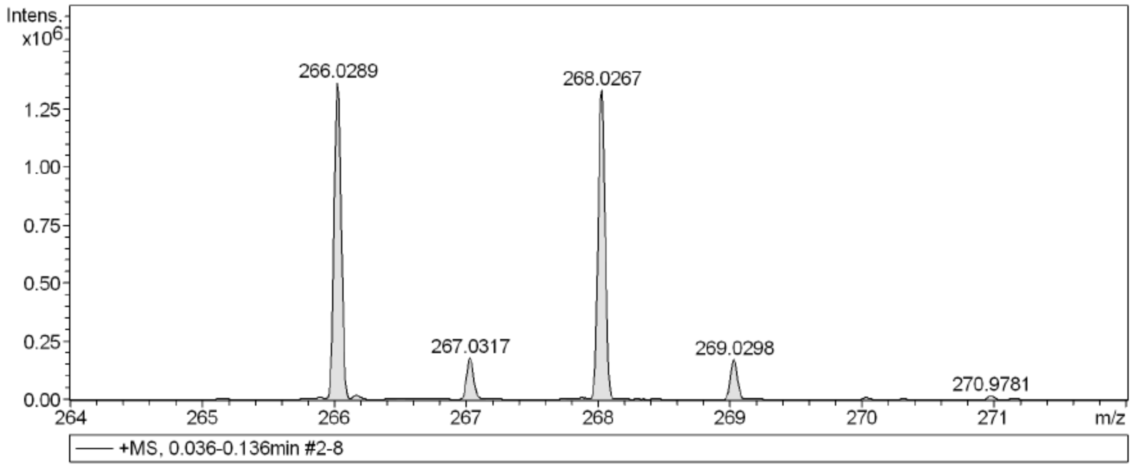

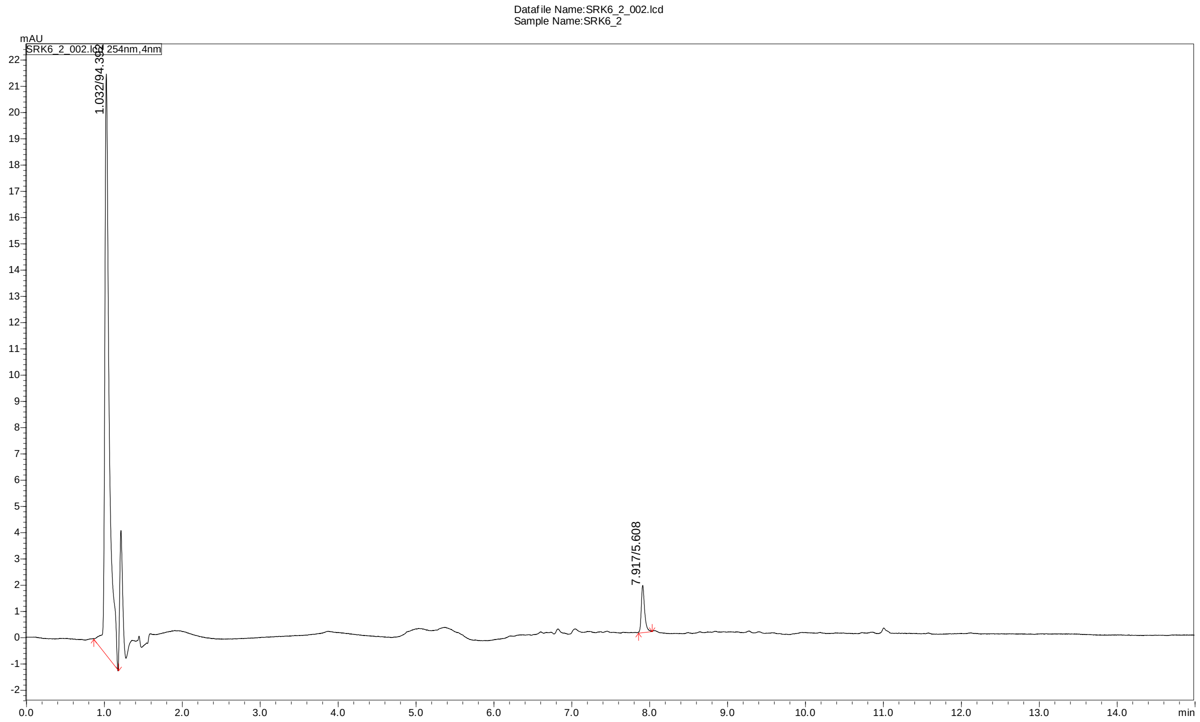

Sample	12		188.20 m/z	
	Time	Height	Area	Area%
1.	1.032	21714	86920	94.392
2.	7.917	1764	5164	5.608
Total		23478	92084	100.000

Mass Spectrum List Report

Analysis Info

Analysis Name	D:\Data\Zlecenia\2023_07_31\SRK6_2_000001.d	Acquisition Date	7/31/2023 9:59:20 AM
Method	tune_low.m	Operator	BDAL@DE
Sample Name		Instrument / Ser#	micrOTOF 213750.10372
Comment	MeOH		

Acquisition Parameter

Source Type	ESI	Ion Polarity	Positive	Set Nebulizer	0.4 Bar
Focus	Not active			Set Dry Heater	180 °C
Scan Begin	50 m/z	Set Capillary	4500 V	Set Dry Gas	4.0 l/min
Scan End	3000 m/z	Set End Plate Offset	-500 V	Set Divert Valve	Waste

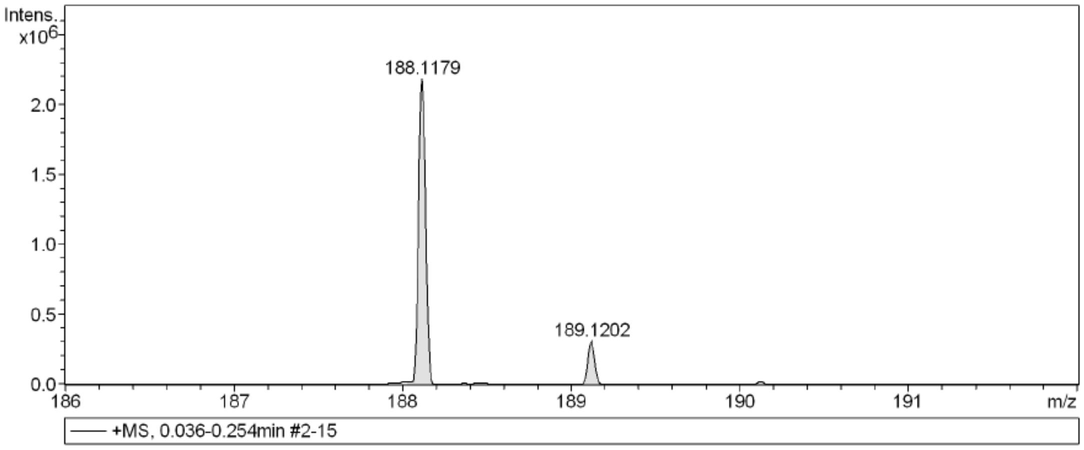

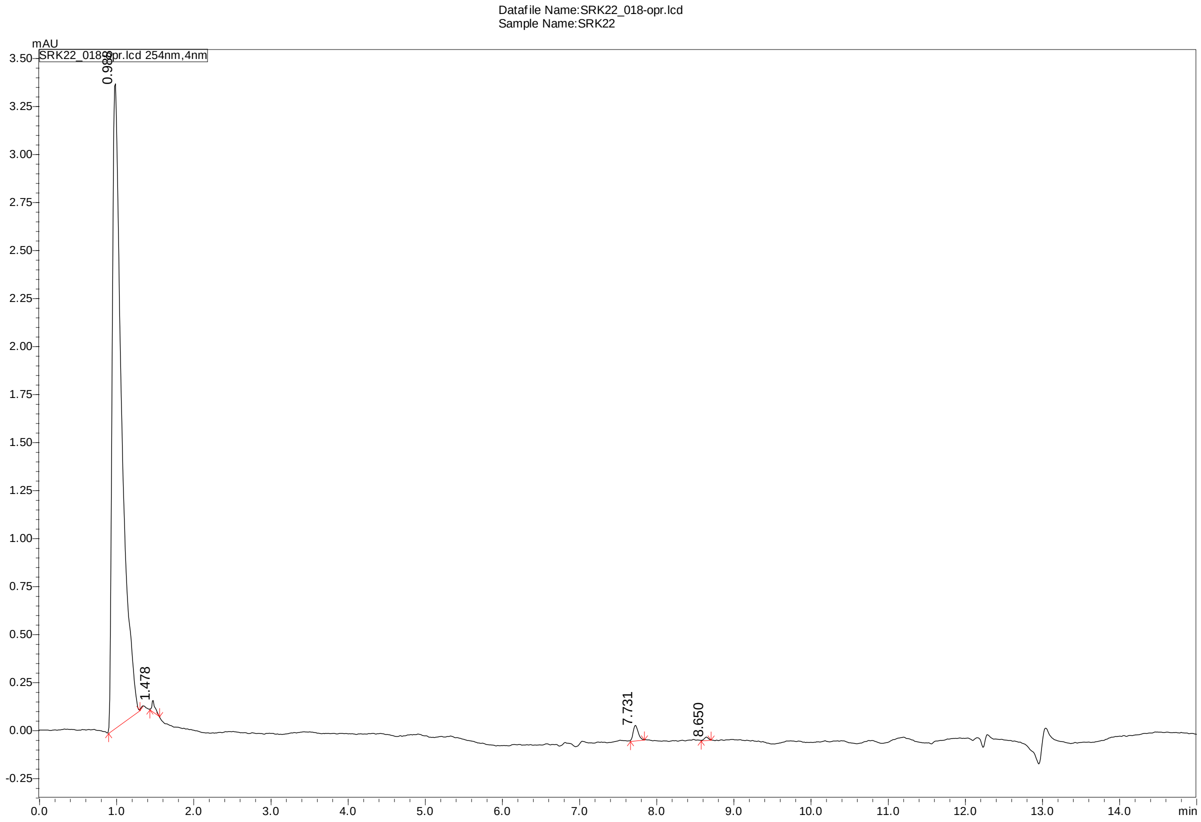

Sample	13		206.30 m/z	
	Time	Height	Area	Area%
1.	0.988	3353	28602	98.099
2.	1.478	59	165	0.566
3.	7.731	78	342	1.171
4.	8.650	15	48	0.164
Total		3505	29157	100.000

Mass Spectrum List Report

Analysis Info

Analysis Name	D:\Data\Zlecenia\2023_03_22\SRK22_000001.d	Acquisition Date	3/22/2023 12:09:55 PM
Method	tune_low.m	Operator	BDAL@DE
Sample Name		Instrument / Ser#	micrOTOF 213750.10372
Comment	MeOH		

Acquisition Parameter

Source Type	ESI	Ion Polarity	Positive	Set Nebulizer	0.4 Bar
Focus	Not active			Set Dry Heater	180 °C
Scan Begin	50 m/z	Set Capillary	4500 V	Set Dry Gas	4.0 l/min
Scan End	3000 m/z	Set End Plate Offset	-500 V	Set Divert Valve	Waste

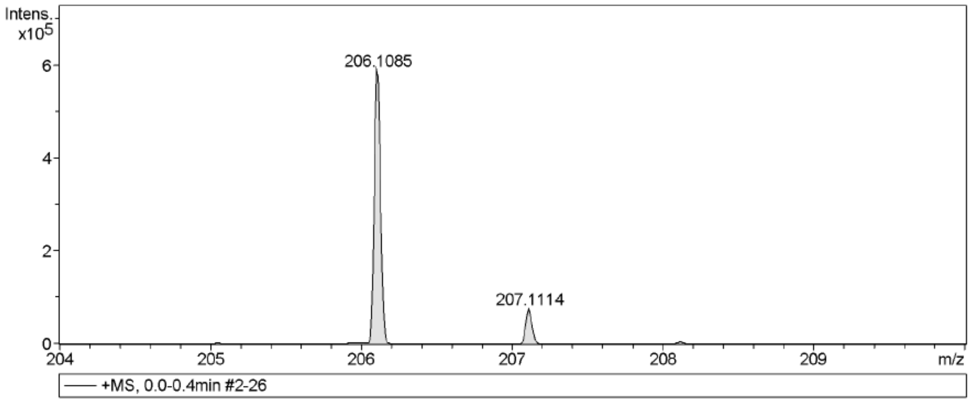

Datafile Name:SRK104-1pr_002-opr.lcd
Sample Name:SRK104-1pr

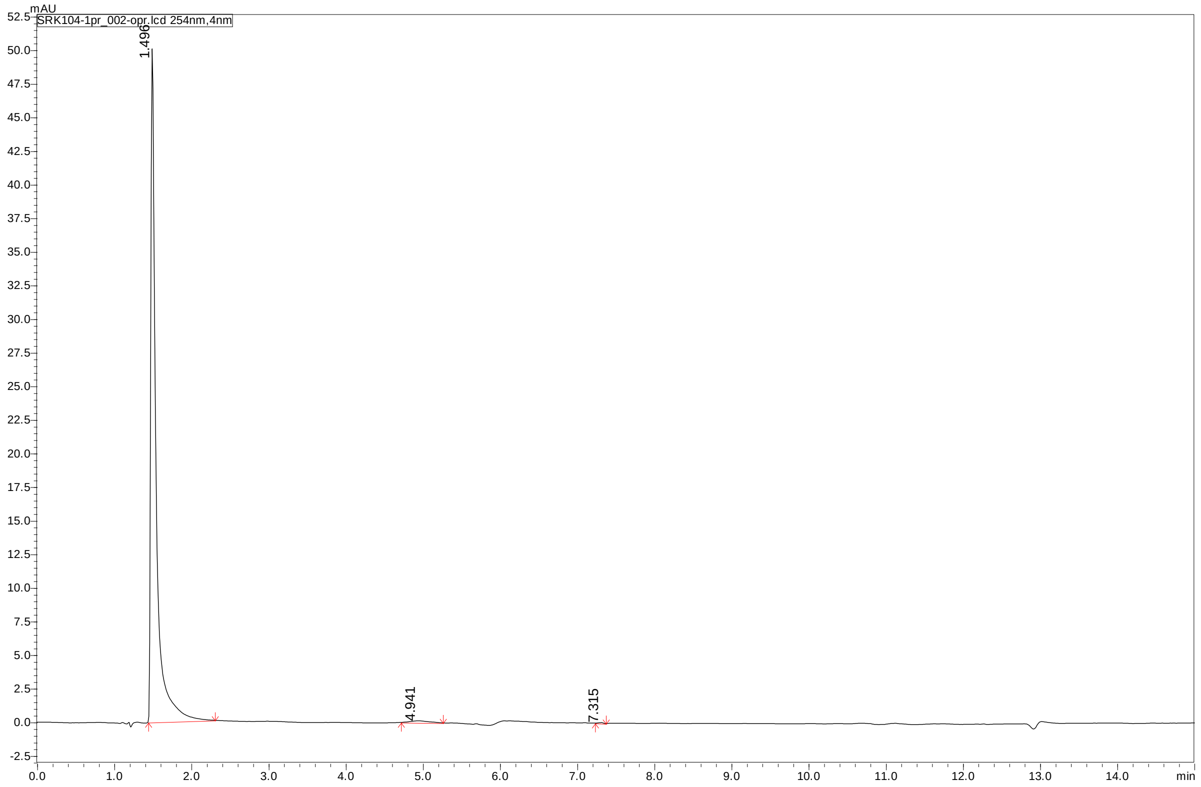

Sample	14		218.30 m/z	
	Time	Height	Area	Area%
1.	1.496	50104	239120	98.999
2.	4.941	135	2205	0.913
3.	7.315	55	212	0.088
Total		50294	241537	100.000

Mass Spectrum List Report

Analysis Info

Analysis Name	D:\Data\Zlecenia\2023_03_22\SRK104_000001.d	Acquisition Date	3/22/2023 12:15:18 PM
Method	tune_low.m	Operator	BDAL@DE
Sample Name		Instrument / Ser#	micrOTOF 213750.10372
Comment	MeOH		

Acquisition Parameter

Source Type	ESI	Ion Polarity	Positive	Set Nebulizer	0.4 Bar
Focus	Not active			Set Dry Heater	180 °C
Scan Begin	50 m/z	Set Capillary	4500 V	Set Dry Gas	4.0 l/min
Scan End	3000 m/z	Set End Plate Offset	-500 V	Set Divert Valve	Waste

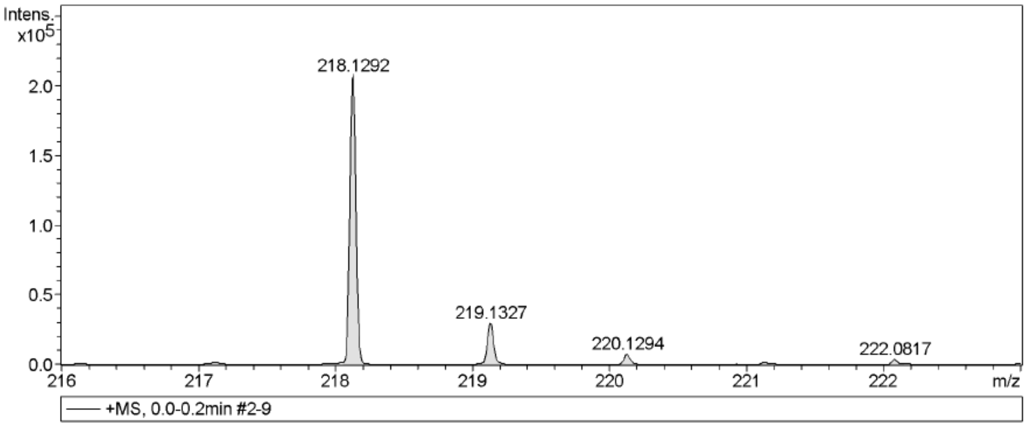

Datafile Name:SRK199-1pr_002.lcd
Sample Name:SRK199-1pr

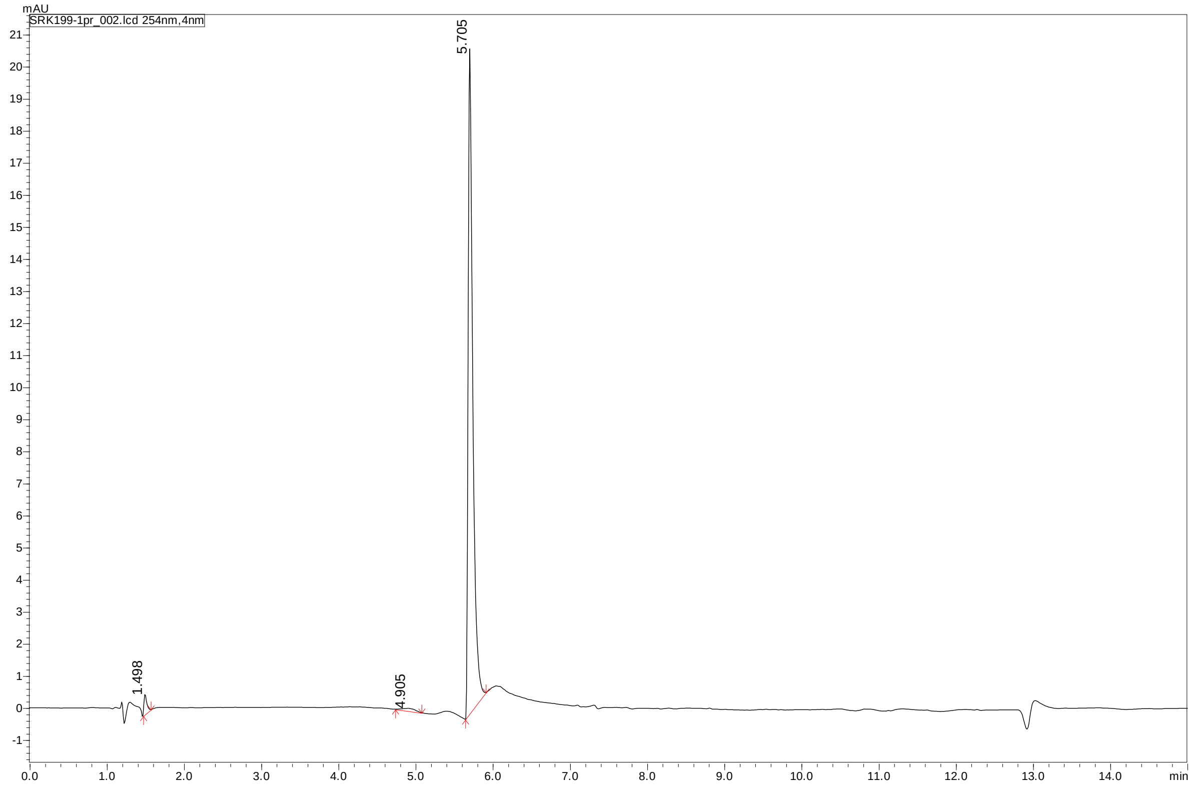

Sample	SRK15		280.35 m/z	
	Time	Height	Area	Area%
1.	1.498	609	1599	1.789
2.	4.905	80	892	0.998
3.	5.705	20712	86929	97.214
Total		21401	89420	100.000

Mass Spectrum List Report

Analysis Info

Analysis Name	D:\Data\Zlecenia\2023_05_10\SRK199_000001.d	Acquisition Date	5/10/2023 1:31:07 PM
Method	tune_low.m	Operator	BDAL@DE
Sample Name		Instrument / Ser#	micrOTOF 213750.10372
Comment	MeOH		

Acquisition Parameter

Source Type	ESI	Ion Polarity	Positive	Set Nebulizer	0.4 Bar
Focus	Not active			Set Dry Heater	180 °C
Scan Begin	50 m/z	Set Capillary	4500 V	Set Dry Gas	4.0 l/min
Scan End	3000 m/z	Set End Plate Offset	-500 V	Set Divert Valve	Waste

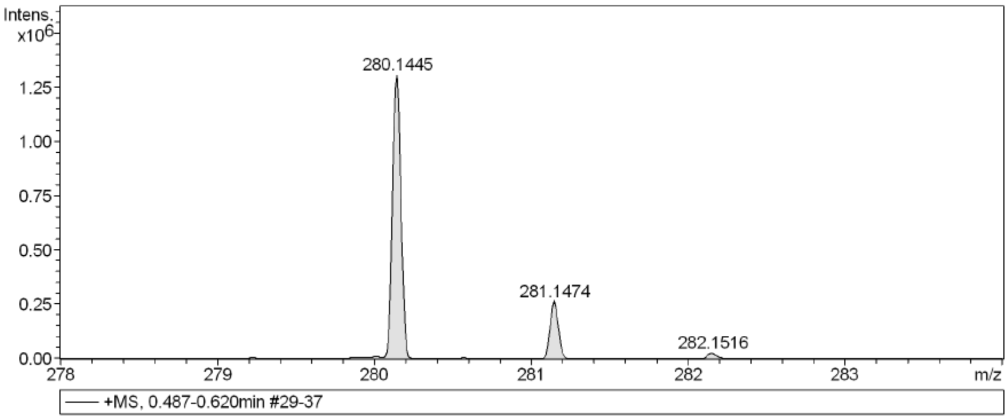

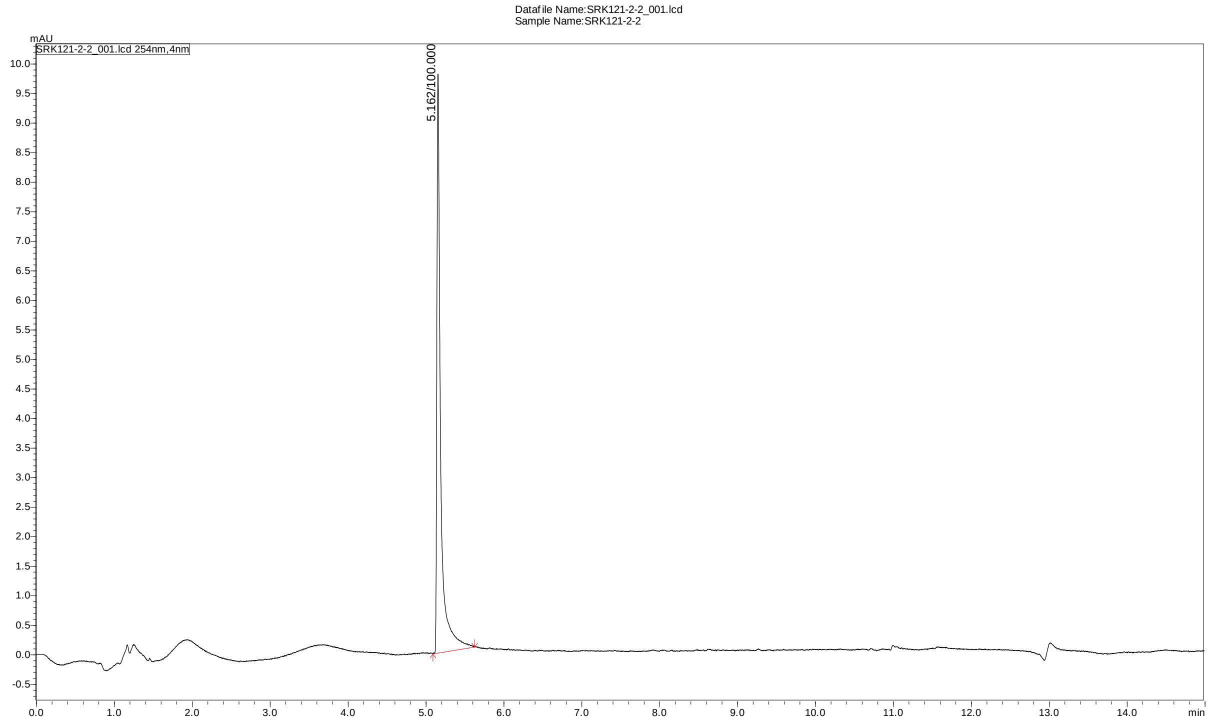

Sample	16		294.30 m/z	
	Time	Height	Area	Area%
1.	5.162	9513	32922	100.000
Total		9513	32922	100.000

Mass Spectrum List Report

Analysis Info

Analysis Name	D:\Data\Zlecenia\2023_07_31\SRK121_2_000001.d	Acquisition Date	7/31/2023 10:33:29 AM	
Method	tune_low.m	Operator	BDAL@DE	
Sample Name		Instrument / Ser#	micrOTOF	213750.10372
Comment	MeOH			

Acquisition Parameter

Source Type	ESI	Ion Polarity	Positive	Set Nebulizer	0.4 Bar
Focus	Not active			Set Dry Heater	180 °C
Scan Begin	50 m/z	Set Capillary	4500 V	Set Dry Gas	4.0 l/min
Scan End	3000 m/z	Set End Plate Offset	-500 V	Set Divert Valve	Waste

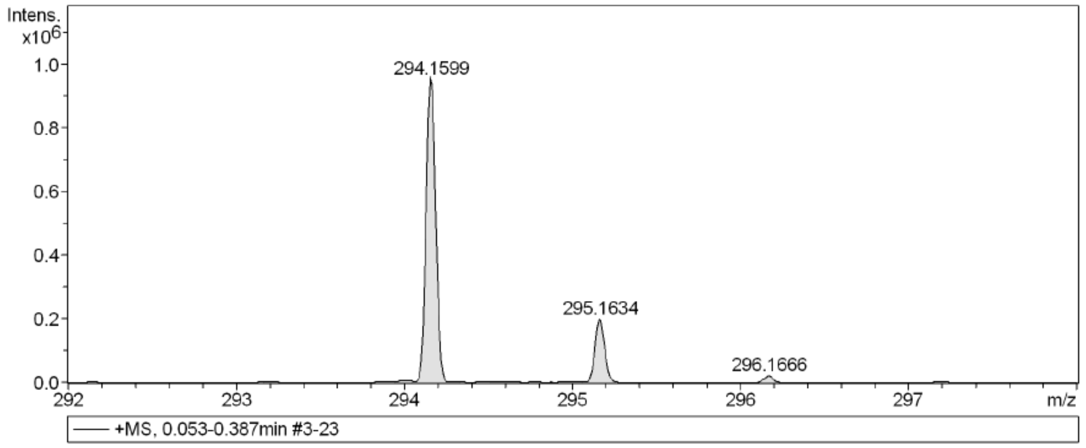

Datafile Name:SRK3_002.lcd
Sample Name:SRK3

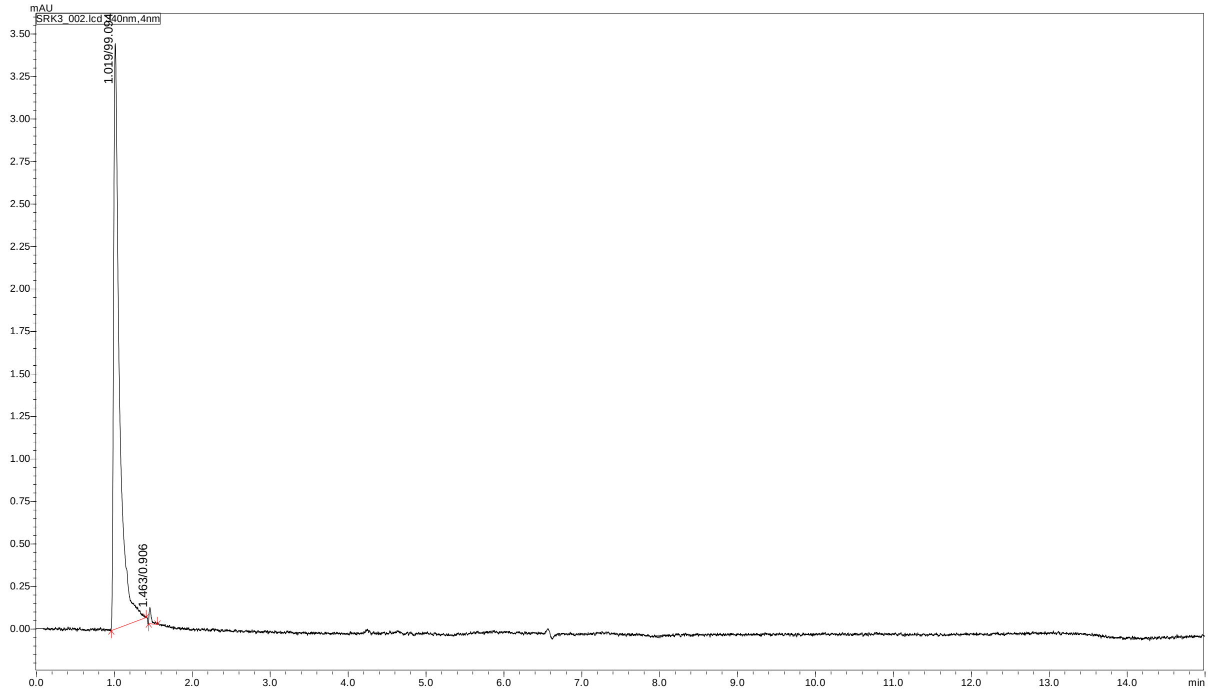

Sample	17		222.15 m/z	
	Time	Height	Area	Area%
1.	1.019	3417	17858	99.094
2.	1.463	86	163	0.906
Total		3502	18022	100.000

Mass Spectrum List Report

Analysis Info

Analysis Name	D:\Data\Zlecenia\2023-04-26\SRK3_2_000001.d	Acquisition Date	4/26/2023 11:30:08 AM
Method	tune_low.m	Operator	BDAL@DE
Sample Name		Instrument / Ser#	micrOTOF 213750.10372
Comment	MeOH		

Acquisition Parameter

Source Type	ESI	Ion Polarity	Positive	Set Nebulizer	0.4 Bar
Focus	Not active			Set Dry Heater	180 °C
Scan Begin	50 m/z	Set Capillary	4500 V	Set Dry Gas	4.0 l/min
Scan End	3000 m/z	Set End Plate Offset	-500 V	Set Divert Valve	Waste

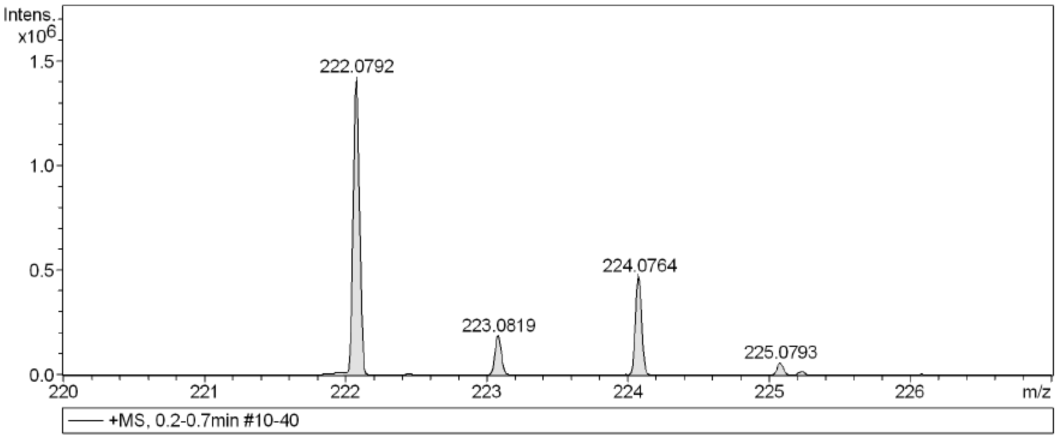

Supplement: Supplementary file 1 — jm3c00877_si_001.pdf [file jm3c00877_si_001.pdf]
